# Using Large Language Models to Generate Dietary Feedback Similar to Human Experts in Weight Management: Experiments on Real-World Scenario Data

**DOI:** 10.3390/bioengineering13040468

**Published:** 2026-04-16

**Authors:** Ruixin Dai, Liping Cui, Kun Hu, Jiye An, Ning Deng

**Affiliations:** 1 Ministry of Education Key Laboratory of Biomedical Engineering, College of Biomedical Engineering and Instrument Science, Zhejiang University, Hangzhou 310027, China; agreewithu@zju.edu.cn (R.D.);; 2 General Hospital of Ningxia Medical University, Yinchuan 750004, China

**Keywords:** large language model, weight management, dietary feedback, real-world scenario data, topic modeling

## Abstract

Providing dietary feedback is important for promoting healthy behaviors in weight management, but the rapid development of obesity and the shortage of medical nutrition human resources have limited this health service. The rise of large language models (LLMs) offers the possibility of using artificial intelligence (AI) to simulate the behavior of human dietitians. However, existing studies have only explored LLM performance when generating answers to common nutrition-related questions; the use of LLMs to generate situation-adapted dietary feedback in practical weight management scenarios still needs further research. In this study, we collected dietary records and dietary feedback from primary dietitians through an mHealth weight management application. We conducted topic modeling to generalize how dietitians deliver nutrition guidance in real-world dietary feedback scenarios. Combining the in-context learning capability of LLMs with real-world data, we proposed a synthetic data generation approach (HDI-SDG) and trained an LLM for dietary feedback with the synthetic data (LLMDF-EXP). Experiments on automatic and manual evaluation of LLMDF-EXP and an LLM trained directly with the real-world data as well as generalized LLMs illustrated that LLMDF-EXP performed most similarly to human experts. Notably, there were no significant differences from human experts in terms of professionalism (*p*-value = 0.510) and usefulness (*p*-value = 0.498). The study highlights that integrating LLMs with real-world data in health management processes can enhance the situational adaptability of LLMs in practical health management environment applications.

## 1. Introduction

Overweight and obesity are significant risk factors associated with numerous chronic diseases, including diabetes and cardiovascular conditions, and have emerged as critical global health challenges affecting billions of individuals and placing substantial burdens on public health systems [1,2]. According to the World Health Organization, approximately 43% of adults worldwide were overweight and 16% were obese in 2022, representing a nearly two-fold increase since 1990. Providing personalized dietary feedback based on individuals’ food intake is an important way to improve eating behaviors and effectively manage weight in overweight populations [3]. Although the proliferation of mobile health (mHealth) weight management applications has enhanced user convenience in tracking daily dietary intake and access to dietitians for tailored dietary guidance [4], the scarcity of human resources for medical nutrition and the need for extensive dietary feedback advice limit the accessibility of such health services [5]. Thus, there is a growing need to develop artificial intelligence (AI) technologies to automatically generate dietary feedback, thereby alleviating dietitian workloads and increasing access to personalized nutrition guidance for weight management participants.

Automated dietary feedback AI systems utilizing knowledge graphs [6] or expert knowledge-based decision trees [7] can analyze user-provided dietary intake information and trigger designed feedback templates by matching the nutrition requirements with established dietary guidelines and best practices. Despite the efficiency and accuracy of these systems, they are unable to respond to inputs outside their pre-constructed knowledge bases, and the restricted templates cannot provide dietary feedback to clients in the same flexible and expressive manner as human experts. Furthermore, the broader landscape of digital health has been significantly advanced by the progress of robust artificial intelligence techniques, ranging from specific neural network architectures for particular clinical tasks [8] to utilizing powerful foundation models to handle increasingly complex healthcare tasks [9]. Building upon this trajectory, large language models (LLMs), particularly conversational LLMs with strong instruction-following capabilities such as GPT-3.5, GPT-4, and ChatGLM, have recently gained significant attention in healthcare [10]. Trained on extensive textual datasets, LLMs acquire rich knowledge and are able to respond with flexible expressions to a wide range of inputs [11,12]. Several studies have demonstrated promising applications of LLMs in response to patients’ messages [13,14,15,16,17]. For instance, a recent study used role-play prompts to find that LLMs performed better than actual primary care physicians in responding to patients’ messages in terms of empathy, responsiveness, accuracy, and usefulness, and the LLM fine-tuned with local data exhibited behavior similar to the actual primary care physicians [17]. Preliminary studies have also begun exploring the potential of LLMs in providing dietary advice [18,19,20]. Although these investigations indicate that LLMs can successfully pass professional nutrition exams and provide higher response quality than dietitians to common nutrition-related questions, making LLMs act like human experts and deliver customized situation-adapted responses in specific dietary feedback practical scenarios still needs further research.

In this study, we collected dietary records submitted by weight management participants and corresponding dietary feedback messages provided by primary dietitians through an mHealth application. Using latent Dirichlet allocation (LDA)—a widely applied unsupervised machine learning technique for topic modeling and text mining [21]—we identified and summarized key themes addressed by dietitians during real-world dietary feedback sessions. To enable LLMs to generate informative responses while incorporating human dietitians’ expressions in real-world dietary feedback scenarios, we proposed a human data induced synthetic data generation (HDI-SDG) method. This method leveraged the generalization capability and in-context learning feature of the LLM to reconstruct dietary feedback messages composed of similar topical content. Then, we fine-tuned an open-source LLM, ChatGLM3-6B, to get two specialized LLMs: the LLM for dietary feedback (LLMDF, https://huggingface.co/Rx-ZJU/LLMDF, accessed on 9 April 2026) and an extended version (LLMDF-EXP, https://huggingface.co/Rx-ZJU/LLMDF-EXP, accessed on 9 April 2026) using the real-world and synthetic data, respectively. Through automatic and manual evaluations, we demonstrated that LLMDF-EXP outperformed both the LLMDF and generalized LLMs, providing feedback most closely aligned with expert dietitians in realistic weight management scenarios.

## 2. Materials and Methods

### 2.1. mHealth Weight Management

**Figure 1:**
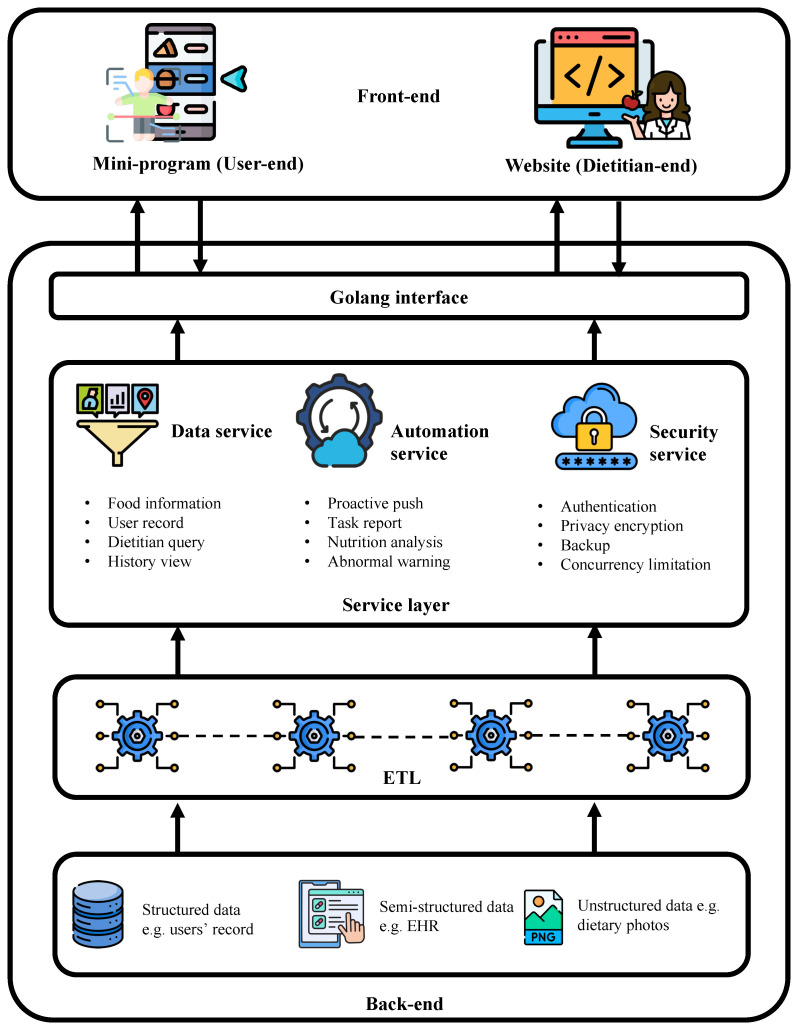
Architecture of the mHealth weight management application. The mHealth weight management application back-end provided a unified RESTful style API to the WeChat mini-program user-end and the website dietitian-end. A user-friendly GUI was designed for the participants and dietitians to efficiently communicate with each other. We extracted daily management data from the application.

We developed an mHealth weight management application, which consists of a WeChat mini-program for users and a website for dietitians. Primary dietitians used this application to provide lifestyle guidance to participants in their communities, aiming to help them to manage their weight and establish healthy habits [22]. The implementation of this program was approved by the ethics committee of Zhejiang University’s School of Public Health (approved number ZGL202112-2) and was registered at https://www.chictr.org.cn, accessed on 9 April 2026 (registration number is ChiCTR2200055548). Upon registration, dietitians assisted participants in entering basic information and trained them on how to use the application. Participants were informed and consented to the use of their data for scientific research during registration. Daily tasks included recording dietary intake, physical activities, and body metrics such as weight, waist circumference, blood pressure, and glucose levels were set and the participants were encouraged to complete these tasks using the mini-program. Dietitians reviewed the submitted dietary records and body metrics, assigned management plans, and provided feedback via the website. Interaction messages were delivered through a backend message queue service prompting participants to receive notifications within their WeChat applications to view the feedback in the mini-program. The application’s back-end and front-end were developed using the Go programming language and Vue framework, respectively. The architecture of the application is illustrated in Figure 1.

### 2.2. Data Collection and Preprocessing

All data was stored on and extracted from the mHealth weight management application server, which contained demographic data, user-submitted records, dietary plans, feedback and communication from dietitians. Our dataset consisted of information from 1103 users and was collected between 2 May 2022and 24 March 2024. Specifically, we extracted 8338 body metrics entries, 25,825 dietary records, and 7747 feedback messages from 20 primary dietitians. Demographic data encompassed user identification, gender, birth date, labor intensity (classified as light, medium, or high), and height. Body metrics records contained user identification, submission timestamps (including year, month, day, hour, minute, second), weight, and waist measurement. Dietary records contained user identification, submission timestamps, meal type (breakfast, lunch, dinner), and food intake details. Feedback messages were linked with specific dietary records and included timestamps as well as the textual content provided by the dietitians.

We integrated dietary records with demographic data and body metrics, aligning them by user identification and matching the closest time points. After de-duplicating and de-identifying the data, we obtained 23,539 dietary records containing health information, with 7305 of these records having corresponding dietary feedback.

### 2.3. LDA Topic Model

To gain knowledge of how dietitians provided dietary feedback to participants in the weight management programs, we applied the LDA topic model to analyze the feedback messages. First, we processed food entries from the 23539 dietary records to develop a food domain dictionary, which was then used with the Chinese text segmentation tool “jieba” [23]. Subsequently, 7305 dietary feedback messages were segmented into words, with punctuation marks and particles removed prior to applying the LDA topic model. We evaluated the results using the $C_{v}$ coherence metric [24] and LDAvis visualization [25] (Supplementary Material Figure S1). Collaborating with medical nutrition experts from the School of Public Health at Zhejiang University, we identified and agreed upon five key topics, as presented in Table 1.

### 2.4. Data Augmentation by HDI-SDG

**Figure 2:**
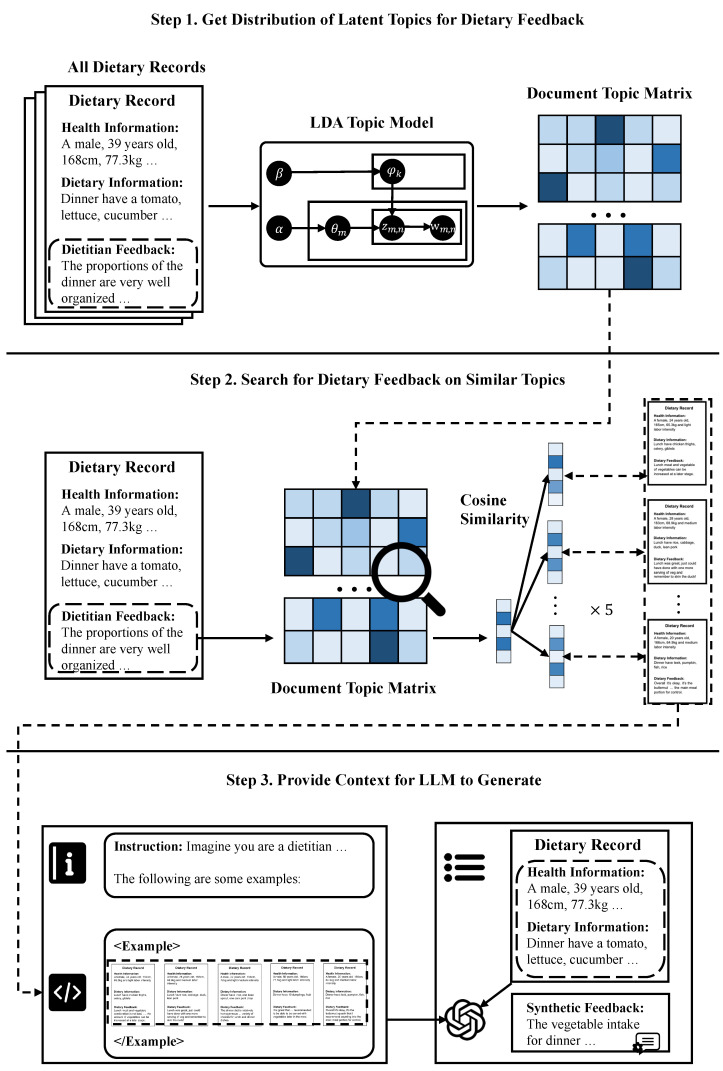
Step-by-step process of the HDI-SDG method. First, we used the LDA topic model to obtain the latent topic distribution of dietitian dietary feedback. Next, for the dietary feedback corresponding to each dietary record we looked for the five dietary feedback messages with the most similar topic compositions as well as their corresponding dietary records. Following this, the five dietary feedback messages and the corresponding dietary records were sent to GPT-3.5 as in-context examples to get the LLM synthesized dietary feedback for the dietary record. As detailed in Section 3.2, we found that our approach allowed GPT-3.5 to synthesize dietary feedback that was informative and situation-adapted.

Figure 2 outlines the HDI-SDG methodology for bootstrapping GPT-3.5 to reconstruct dietary feedback based on real-world data from dietitians. In step 1, we employed LDA topic modeling to identify the distribution of the five topics within dietary feedback. In step 2, for each dietary record and its associated feedback message, we used cosine similarity to search for the five dietary feedback messages with the most similar topic compositions (except itself) and their related dietary records on the dietary feedback topic distribution. In step 3, these five dietary records and feedback messages were presented to GPT-3.5 as in-context examples [12], prompting the model to reconstruct the dietary feedback for the given dietary record. The full prompt can be found in Supplementary Material Prompt S1. This approach yielded 7305 dietary records, each accompanied by dietary feedback synthesized by GPT-3.5 guided under human data.

### 2.5. Model Development

In this study, we developed two models for dietary feedback based on ChatGLM3-6B, which features an appropriate parameter size and demonstrates good performance on both Chinese and English tasks [26]. The first model, LLMDF, was fine-tuned exclusively using the dataset of 7305 dietary records paired with their original real-world feedback from dietitians (100% real data). The second model, LLMDF-EXP, was fine-tuned exclusively using the 7305 records paired with the synthetically generated feedback produced by GPT-3.5 via the HDI-SDG method (100% synthetic data). In preparation for the fine-tuning process, the training data were formatted in the Stanford Alpaca [27] template. As detailed in Supplementary Material Prompt S2, the prompt instructed the language model to simulate a dietitian, quoting feedback strategies within the instruction section. Dietary and health information was provided in XML format in the input section, while the corresponding XML dietary feedback was defined in the output section [28]. Leveraging low-rank adaptation [29], we then performed supervised fine-tuning using the arranged data. We conducted the fine-tuning process with LLaMA-Factory framework [30] and the hyperparameters used for the fine-tuning were set as follows: Optimizer: AdamW, target module: QKV, batch size: 8, learning rate: 5 $\times 10^{-5}$, epochs: 4, lora r: 8, lora alpha: 16, and lora dropout: 0. The hyperparameters were initially selected based on the recommended default settings for instruction fine-tuning in the LLaMA-Factory framework and further refined through empirical testing. To prevent overfitting, we randomly held out 20% of the training data as a validation set and employed an early stopping mechanism, evaluating the validation loss and saving checkpoints every 200 steps during the training process.

### 2.6. Evaluation Dataset

To evaluate the models, we selected 20 real-world dietary scenarios covering different demographic characteristics, varying degrees of overweight status, and various meal types from the remaining 16,234 dietary records (the 23,539 dietary records excluding the 7305 dietary records with dietitian feedback, as these 7305 dietary records had already been used to train the models). Two medical nutrition experts reviewed these scenarios and crafted corresponding dietary feedback, which was subsequently verified by an additional expert. We employed the LLMDF and LLMDF-EXP to generate dietary feedback for the selected dietary scenarios using the prompt detailed in Supplementary Material Prompt S3. For comparison, we also used ChatGLM3-6B, GPT-3.5 and GPT-4 to generate dietary feedback under the same prompt conditions. The evaluation dataset can be found in Supplementary Material Table S6 and its statistical overview is presented in Supplementary Material Figure S2.

### 2.7. Automatic Evaluation

We used the dietary feedback crafted by human experts as a benchmark to automatically evaluate the outcomes generated by LLMDF, LLMDF-EXP, ChatGLM3-6B, GPT-3.5, and GPT-4 on the evaluation dataset. For measuring textual similarity, we employed well-established metrics based on word overlap: BLEU [31], ROUGE-L [32], and METEOR [33], all of which are commonly used in the text generation domain. Additionally, to evaluate semantic similarity, we computed precision ($P_{BERT}$), recall ($R_{BERT}$), and F1 scores ($F_{BERT}$) using BERTScore [34]. This combination of metrics allows us to automatically measure how similar the outputs are to those of human experts. Meanwhile, it is important to note that dietary feedback generation is a creative task wherein the same nutritional advice can be expressed in various ways. Therefore, n-gram based metrics (BLEU, ROUGE-L, METEOR) were strictly used to analyze lexical similarity, structural comparability, and phrasing alignment with the reference texts, rather than to measure the clinical quality or factual correctness of the advice.

### 2.8. Human Evaluation

For each dietary scenario in the evaluation dataset, we randomized the sequence of dietary feedback produced by human experts and the LLMs. We engaged four clinical dietitians from external organizations, all with experience in weight management, to assess each feedback using a 5-point Likert scale (1 = strongly disagree, 5 = strongly agree) across four dimensions: (1) Empathy: The dietary feedback reflects an understanding of the physical and psychological challenges encountered by individuals engaged in weight management. (2) Completeness: The feedback provides comprehensive and sufficient information. (3) Professionalism: The feedback is nutritionally accurate, tailored and soundly responses to the given dietary scenario. (4) Usefulness: I can use it as a template to write my feedback to this dietary scenario. Each dietitian read a total of 30,656 words of feedback and completed 480 rating decisions during the evaluation; the process is shown in Supplementary Material Figure S3. The dietitians were also permitted to include additional comments on each piece of feedback. They were blinded to the origin of the feedback, and their evaluations were conducted independently to maintain impartiality and integrity of the process.

### 2.9. Statistical Analysis

For the human evaluation, we analyzed the expert ratings and applied the Kruskal–Wallis test to compare dietary feedback generated via the various methods. The statistical analyses were conducted using Python 3.9.

## 3. Results

### 3.1. Dietary Feedback Data

**Figure 3:**
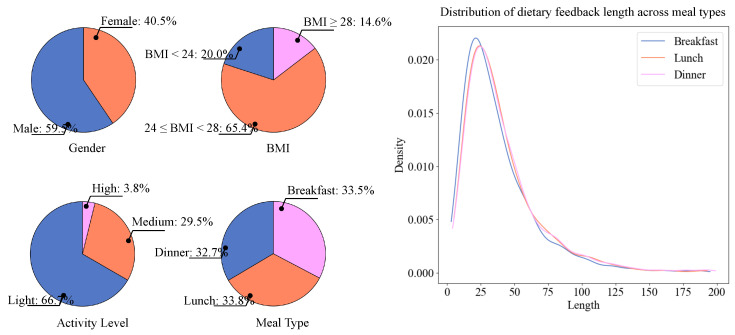
Gender, BMI, activity level distribution of the weight management participants and distribution of meal types and dietary feedback lengths in the dietary records.

We developed an mHealth weight management application for communities in urban areas of southeastern China and collected 25,825 dietary records from 1103 users involved in weight management and lifestyle interventions. After processing and de-identification, a total of 23,539 dietary records, among which 7305 had corresponding primary dietitian feedback, were included in our study. The statistical information of the dietary records is shown in Figure 3.

### 3.2. LDA Topic Model and Data Augmentation by HDI-SDG

**Figure 4:**
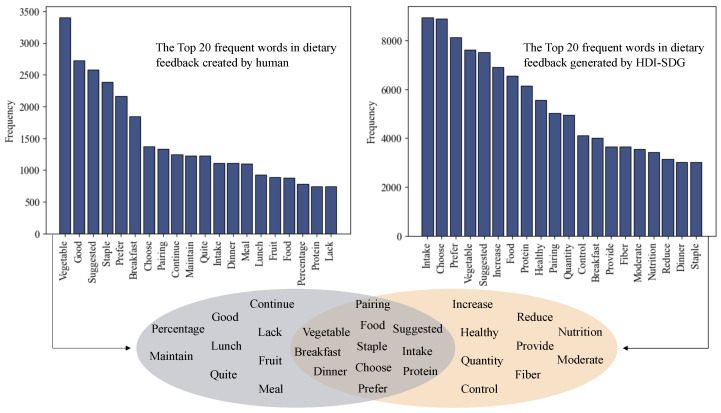
Word preference in human-created and human data induced LLM generated dietary feedback.

**Figure 5:**
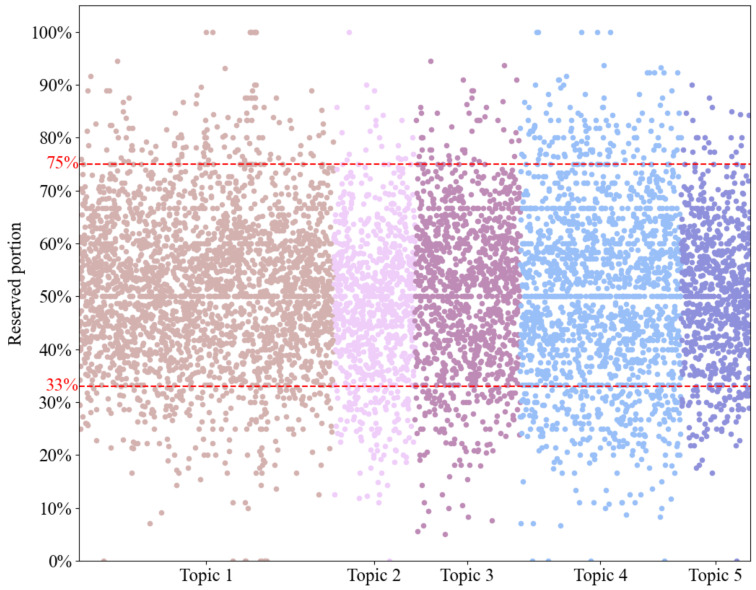
Reserved percentage in the reconstructed dietary feedback among samples. Topic numbers correspond to Table 1.

To gain knowledge of how human dietitians provided dietary feedback to weight management participants, we applied an LDA topic model to the 7305 dietary feedback messages. This analysis revealed five latent topics: (1) optimizing meat and vegetable combinations, (2) analyzing food properties, (3) feedback on breakfast pairings, (4) encouragement to maintain healthy eating habits, and (5) increasing intake of coarse grains (Table 1). Using these topics, we created prompts (Supplementary Material Prompt S1) and submitted the dietary records corresponding to the 7305 feedback messages to GPT-3.5. For each record, we provided GPT-3.5 with examples of similar topic compositions as context, prompting it to generate synthetic dietary feedback data. The mean character count (SD) of human data induced GPT-3.5 generated feedback was 124.67 (61.06), compared to 39.85 (28.94) for feedback created by primary dietitians, indicating that the synthetic dietary feedback was more informative. We also calculated word frequencies of them separately. We found that 11 of the 20 most frequent words were common to both datasets (Figure 4), and the percentage of reserved words ranged from approximately 33% to 75% across the samples (Figure 5). This suggests that the human data induced GPT-3.5 generated dietary feedback successfully retained the linguistic conventions of human-created dietary feedback in real-world scenarios. We used the dietary feedback created by the primary dietitians and the human data induced GPT-3.5 generated dietary feedback to fine-tune ChatGLM3-6B to obtain LLMDF and LLMDF-EXP, respectively, and further investigated their performance.

### 3.3. Automatic Evaluation

**Figure 6:**
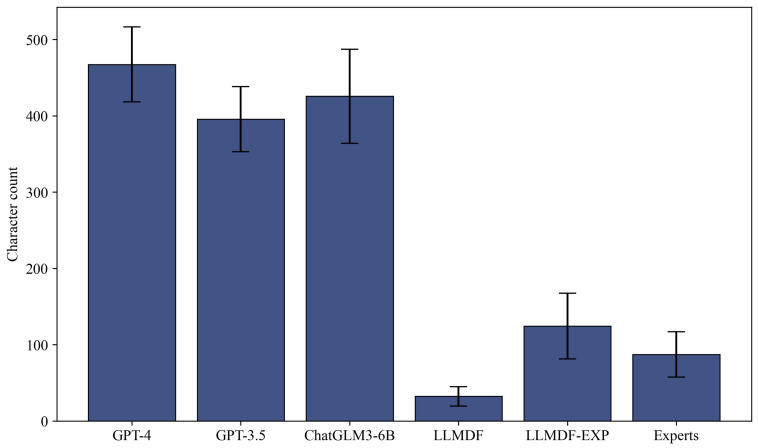
Length of dietary feedback generated in different ways. The length of dietary feedback generated by GPT-4, GPT-3.5, ChatGLM3-6B, LLMDF, LLMDF-EXP and human experts on the evaluation dataset, with error bars showing standard deviation.

For evaluation, we constructed a benchmark by selecting 20 real-world dietary scenarios from the mHealth weight management and obtaining corresponding dietary feedback from three medical nutrition experts. We computed automatic evaluation metrics, including BLEU [31], ROUGE-L [32], METEOR [33] and BERTScore [34] for LLMDF-EXP, LLMDF, and generalized LLMs (GPT-4, GPT-3.5 and ChatGLM3-6B) taking the expert-crafted feedback as reference text. As shown in Table 2, LLMDF and LLMDF-EXP achieved higher ROUGE-L and $P_{BERT}$ scores compared to the generalized LLMs, indicating that the fine-tuned models generated dietary feedback whose expression more closely resembled that of human experts. However, when considering the length of the generated feedback (Figure 6), we noted that LLMDF had lower BLEU, METEOR, and $R_{BERT}$ scores than the generalized LLMs. This suggests that directly fine-tuning with primary dietitian-created data may lead to omissions in the generated feedback. LLMDF-EXP demonstrated optimal or near-optimal performance across all evaluation metrics, indicating that its generated feedback was not only expressively similar to expert feedback but also retained comprehensive information.

### 3.4. Human Evaluation

To gain a more comprehensive understanding of the models’ capabilities in generating dietary feedback, we enlisted four clinical dietitians with extensive experience in weight management to evaluate the feedback produced by the LLMs and human experts. The dietitians assessed the feedback across four dimensions: empathy, completeness, professionalism, and usefulness, using a scale ranging from 1 (strongly disagree) to 5 (strongly agree). The evaluators had an average of 9.75 years of clinical practice, with all holding intermediate-level titles or higher.

**Figure 7:**
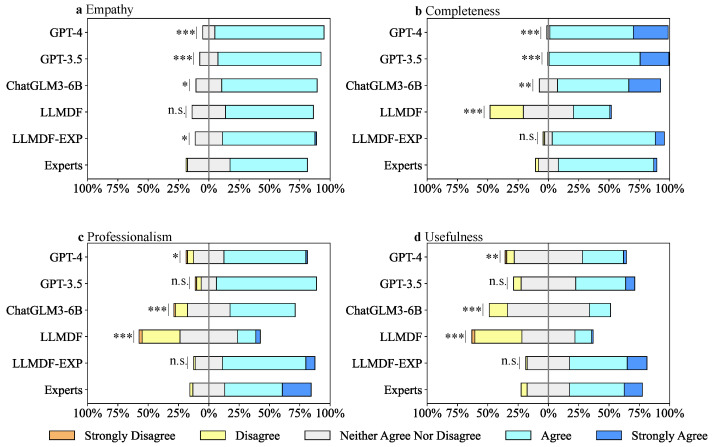
Performance of LLMs in human evaluation. (**a**) Empathy, (**b**) completeness, (**c**) professionalism and (**d**) usefulness. Significant difference between models and human experts, n.s. = not significant, * *p*-value ≤ 0.05, ** *p*-value ≤ 0.01, *** *p*-value ≤ 0.001. The details of Dunn’s test for multiple comparisons are presented in Supplementary Material Tables S2–S5.

Empathy refers to whether the dietary feedback reflects an understanding of the physical and psychological challenges faced by the weight management participant. As illustrated in Figure 7a, the raters provided predominantly positive ratings for the empathy of feedback generated by LLMs, with none rated as disagree or strongly disagree. Notably, feedback from GPT-4, GPT-3.5, ChatGLM3-6B, and LLMDF-EXP significantly surpassed that of human experts in empathy, with *p*-values of 0.001, <0.001, 0.021, and 0.023, respectively. Among these models, GPT-4 achieved the highest empathy scores. No significant difference was observed between LLMDF and human experts.

Completeness refers to whether the dietary feedback provides sufficiently comprehensive information about the given dietary scenario. Figure 7b indicates that the percentages of feedback achieving a “strongly agree” score for completeness were 28.75%, 23.75%, and 26.25% for GPT-4, GPT-3.5, and ChatGLM3-6B, respectively, which was significantly higher than that of human experts. LLMDF performed notably weaker in completeness compared to human experts (*p*-value < 0.001), whereas the augmented fine-tuned model, LLMDF-EXP, performed comparably to human experts (*p*-value = 0.084). We noted that different models tended to generate dietary feedback of varying lengths. As depicted in Figure 6, GPT-4, GPT-3.5, and ChatGLM3-6B tended to produce longer feedback, which may advantage them on completeness scores. In contrast, LLMDF-EXP and human experts generated dietary feedback of moderate length while still achieving positive completeness ratings.

Professionalism refers to whether the dietary feedback is nutritionally accurate, tailored and soundly responds to the given dietary scenario. Figure 7c shows that LLMDF-EXP performed closest to human experts in professionalism, with only 1 case of disagree (LLMDF-EXP) and 2 cases of disagree (human experts), significantly outperforming LLMDF and ChatGLM3-6B with *p*-values both <0.001. We summarized the raters’ comments on dietary feedback generated in different ways (Supplementary Material Table S6). Notably, the dietary feedback generated by LLMDF was perceived as overly simplistic and lacking targeted advice. The distribution of hallucination issues is detailed in Supplementary Material Table S7, with examples of each type provided in Supplementary Material Table S8; we found 15% of comments on ChatGLM3-6B highlighted hallucination issues, including knowledge errors (6 cases), inconsistencies (1 case) and misunderstandings (5 cases). GPT-3.5 and GPT-4 exhibited fewer hallucination issues than ChatGLM3-6B, with 3.75% and 5% of comments noting knowledge errors (1 case for GPT-3.5 and 2 cases for GPT-4) and inconsistencies (1 case for each), respectively. Both models received positive professionalism ratings, with GPT-3.5 not significantly differing from human experts.

Usefulness measures the dietitians’ willingness to adopt the dietary feedback as a template for writing a response to the corresponding dietary scenario. Figure 7d demonstrates that LLMDF-EXP matched human experts’ performance (*p*-value = 0.498), significantly outperforming GPT-4 (*p*-value < 0.001), GPT-3.5 (*p*-value = 0.029), ChatGLM3-6B (*p*-value < 0.001), and LLMDF (*p*-value < 0.001). Based on our interpretation of the qualitative comments provided by the raters, the dietary feedback generated by GPT-4, GPT-3.5 and ChatGLM3-6B was frequently annotated as verbose. In contrast, LLMDF-EXP struck an effective balance between providing sufficient information and remaining concise. In terms of linguistic expression, the dietary feedback generated by GPT-4, GPT-3.5 and ChatGLM3-6B exhibited characteristics of machine generation, being labeled as having overly formalized linguistic organization and potentially repeating some information in the scene prompts. Conversely, the expressions of LLMDF-EXP and human experts showed familiarity and flexibility, which allowed their feedback to be adopted as templates with a notably higher willingness than other models. It should be noted that the high usefulness score of LLMDF-EXP may indicate its success in imitating the existing local practice style that raters are accustomed to, rather than necessarily providing clinically superior advice to GPT-4.

## 4. Discussion

### 4.1. Principal Findings

To address the persistent challenge of automating dietary guidance in real-world weight management and to alleviate the workload of dietitians amidst shortages in medical nutrition human resources, our study developed a novel pipeline that translates expert dietitian behaviors into an effective LLM-based system. Specifically, we distinguished five topics from primary dietitians’ dietary feedback using the LDA topic modeling method, and these clinical insights directly informed our HDI-SDG pipeline. By retrieving contextually similar real-world scenarios, HDI-SDG effectively guided the foundation model to generate high-quality, situation-adapted training pairs. We then fine-tuned ChatGLM3-6B to create two distinct models: LLMDF and LLMDF-EXP, utilizing data from real-world scenarios and synthesized data, respectively. We mixed generated dietary feedback from LLMDF and LLMDF-EXP with dietary feedback crafted by human medical nutrition experts as well as feedback from generalized LLMs—GPT-3.5, GPT-4 and ChatGLM3-6B. Qualified dietitians evaluated these responses in terms of empathy, completeness, professionalism and usefulness. The results demonstrated that LLMDF-EXP received positive ratings across all evaluation metrics and closely resembled human expert feedback. Compared to LLMDF, LLMDF-EXP provided significantly more complete feedback, and compared to the generalized LLMs, LLMDF-EXP’s feedback was more concise and aligned more closely with human expert expressions, exhibiting significantly enhanced usefulness.

First, little empirical research exists to guide the crafting of dietary feedback messages for weight management [3], which poses a challenge when using LLMs to complete this practical task. Previous studies in healthcare have illustrated that LDA topic modeling can effectively identify underlying themes in health-related information shared by users on social media, and this approach has been leveraged to design targeted interventions and develop patient support systems [35,36]. However, to the best of our knowledge, no existing dataset combines weight management participants’ dietary records with corresponding feedback from dietitians. In light of this, we developed an mHealth weight management application to collect data for analyzing how dietitians practice nutrition guidance in real-world dietary scenarios. Our analysis identified five dietary feedback topics that reflect three key strategies employed by primary dietitians to promote healthy eating behaviors among weight management participants: 1. Providing practical dietary suggestions: Topics 1, 3, and 5 (see Table 1) highlight common dietary issues among participants, such as “inadequate proportion of vegetables”, “high carbohydrate content in breakfast” and “insufficient intake of whole or coarse grains”. Dietitians address these issues by offering operable advice consistent with weight management dietary guidelines and best practices. 2. Enhancing nutritional literacy: By analyzing food intake properties, dietitians can improve participants’ understanding of nutrition, as reflected in dietary feedback topic 2 (Table 1). 3. Offering social support: Dietitians provide emotional support as positive reinforcement to encourage participants to maintain healthy eating habits, as reflected in dietary feedback topic 4 (Table 1). These insights can inform the development of AI-driven weight management expert systems, potentially enhancing their effectiveness in supporting healthy eating behaviors.

Having obtained the real-world dietary feedback data, one natural idea is to directly use it to fine-tune an LLM, which resulted in LLMDF. The model demonstrated its ability to generate dietary feedback that closely resembled the tone and style of local primary dietitians, as evidenced by its highest $P_{BERT}$ score in the automatic evaluation and the absence of “robot-like” annotations in the manual evaluation. However, the quality of the local data limited LLMDF’s performance, leading to omissions of important information and reduced completeness and professionalism. In contrast, generalized LLMs, when prompted with instructions combining role-playing and dietary feedback delivery strategies, performed well in terms of completeness and professionalism. Despite their comprehensiveness, the feedback generated by generalized LLMs often exhibited machine-generated characteristics, such as overly verbose and formalized expressions, which could reduce user acceptance in practical applications [37,38]. To address these limitations, the HDI-SDG approach achieved data augmentation that preserved situational adaptation by providing real-world dietary feedback scenario data under similar topics as context for the appropriate LLM. The resulting model, LLMDF-EXP, trained on this augmented dataset, received consistently positive ratings for empathy, completeness, professionalism, and usefulness (Figure 7). Notably, LLMDF-EXP significantly outperformed generalized LLMs in usefulness, with none of its generated feedback being perceived as robot-like in syntax or tone. However, it should be acknowledged that the usefulness metric inherently reflects the subjective preferences of the evaluating dietitians. The strong preference for LLMDF-EXP over models like GPT-4 likely indicates that it successfully replicates the familiar local practice style and workflow to which raters are accustomed, rather than proving that its nutritional advice is clinically superior to the more comprehensive outputs of generalized LLMs. Overall, the comparison between LLMDF-EXP, LLMDF, and generalized LLMs highlights the potential of combining local data with LLMs to address their respective limitations, thereby enhancing the adaptability and practicality of LLMs in real-world dietary feedback scenarios.

Through a qualitative analysis of annotators’ comments, we identified three types of hallucination issues when using LLMs to generate feedback for real-world dietary scenarios: 1. Knowledge errors, where the LLM-generated feedback contains factual nutritional mistakes. 2. Inconsistencies, where LLMs produce contradictory information when handling complex dietary situations. 3. Misunderstandings, where LLMs misinterpret scenario details provided in the prompts. In our evaluation of professionalism, we found that the open-source LLM, ChatGLM3-6B, was more prone to these hallucination issues compared to OpenAI’s GPT-3.5 and GPT-4 when generating dietary feedback. Specifically, GPT-3.5 and GPT-4 exhibited fewer knowledge errors and inconsistencies, and no misunderstandings were reported during the evaluation (Supplementary Material Table S7). For the generalized models, knowledge errors often involved factual nutritional mistakes (e.g., ChatGLM3-6B incorrectly identifying millet pumpkin porridge as high-calorie, or GPT-4 mistakenly treating corn as a refined grain rather than a whole grain). In contrast, the fine-tuned LLMDF model’s knowledge errors primarily manifested as omissions of necessary dietary guidance for weight loss (e.g., failing to point out the excessive fat content when a user consumed egg fried rice for breakfast). We also observed that generalized models tend to structure responses using bullet points, which occasionally contained conflicting advice (inconsistencies, such as GPT-4 advising an increase in calorie intake in one bullet point while advising a reduction in another). Furthermore, ChatGLM3-6B sometimes could not accurately interpret scenario details (misunderstandings), which may be due to limitations in the model’s capabilities and a lack of specific training in real-world nutritional practice. All the examples mentioned above can be found in Supplementary Material Table S8. Notably, LLMDF-EXP exhibited zero hallucination issues during the evaluation. This demonstrates that our proposed HDI-SDG method successfully mitigates the scenario-related hallucinations (inconsistencies and misunderstandings) common in generalized models by grounding them in a real-world data context. Simultaneously, it avoids the knowledge errors associated with the oversimplification and omission of critical guidance that were observed when fine-tuning directly with real-world data (LLMDF). However, more nuanced design and evaluation measures remain essential when practically applying LLMDF-EXP, given the challenge of completely avoiding knowledge hallucinations [39] and the need to adhere to the complex ethical requirements of healthcare services [40].

### 4.2. Further Implications

We observed that only 30% of submitted dietary records received feedback from dietitians, indicating a limitation in processing capabilities despite the availability of an mHealth environment. In mHealth weight management contexts, deploying LLMs for dietitians to draft dietary feedback could potentially alleviate their workload and enhance the accessibility of dietary feedback services. While generalized LLMs such as GPT-4 have demonstrated proficient knowledge in some nutrition benchmarks [18,19,41], their application in real-world dietary feedback scenarios without customization often produces responses that diverge significantly from those of human dietitians, thus impacting practicality. Our study emphasizes the importance of analyzing and integrating real-world data to address this challenge. Additionally, rather than developing a comprehensive nutrition-domain LLM—which involves substantial costs associated with data collection, processing domain-specific knowledge, and retraining [42]—we opted to create models tailored for specific tasks within the weight management process. Decomposing the doctor–patient communication of health management scenarios into well-defined tasks and integrating LLMs accordingly may offer greater flexibility and facilitate real-world implementation due to clearer application objectives.

Finally, the proposed HDI-SDG pipeline provides a practical approach to improving the quality of real-world doctor–patient communication data captured in mHealth workflows. By integrating expert insights into local data from the initial phase with LLM-generated synthetic data, it reduces the negative effects of long-tail distribution caused by healthcare providers’ interaction preferences in training data, while preserving local context. Future work should develop an iterative real-world data–LLM framework that actively involves healthcare providers, patients, and AI developers in the health management process. This would strengthen the LLMs’ situational awareness [40] of these workflows and accelerate the transformation of health management practices.

### 4.3. Limitations

Our study has several limitations. First, the data we utilized stems from an mHealth weight management program in urban areas of southeastern China. The specific nutritional guidance implemented by dietitians for overweight people may change due to differences in food cultures and levels of economic development across different regions [2,43]. Second, the dataset utilized for training our models was derived from interactions involving only 20 primary dietitians. While we collected data over a two-year period from 1103 users in diverse settings—including citizen centers, workplaces, and residential communities—the small number of experts remains a limitation. This poses a risk of the model LLMDF overfitting to the specific linguistic habits and communication styles of this localized group, rather than capturing the universal diversity of the dietetic profession. Obtaining more broadly applicable dietary feedback models in the future will depend on the long-term construction of large-scale, multi-center data infrastructures across various regions. Third, due to constraints in data size and computing resources, we employed an LLM with a scale of six billion parameters, using parameter-efficient fine-tuning for our experiments. According to scaling laws, training on larger datasets and employing models with a greater number of parameters could potentially yield additional insights [13,44]. Finally, our study evaluated the quality of AI-generated content from the perspective of healthcare providers using a limited sample size. The acceptance of AI technology may differ among various demographics. Future research should consider expanding the sample size and incorporating patient perspectives on the use of LLMs in real-world healthcare settings [45].

## 5. Conclusions

In conclusion, our work has three main contributions. First, we identified five topics from primary dietitians’ dietary feedback practices with the LDA topic modeling method. These five topics reflect three key strategies employed by primary dietitians to promote healthy eating behaviors among weight management participants: (1) Providing practical dietary suggestions. (2) Enhancing nutritional literacy. (3) Offering social support. These findings can inform the development of AI-driven weight management expert systems, potentially enhancing their effectiveness in supporting healthy eating behaviors. Next, our research identified three types of hallucination issues of LLMs when generating dietary feedback for real-world scenarios: (1) Knowledge errors. (2) Inconsistencies. (3) Misunderstandings. Furthermore, although generalized LLMs are good at generating comprehensive responses, these responses often show obvious traces of being machine-generated, which affects the willingness of dietitians to adopt them as templates to complete their work. Fine-tuning with real-world data can alleviate some scenario-related hallucination issues and generate responses in a similar voice to local dietitians, but due to the limitations of real-world data quality, the model’s ability to generate comprehensive information is compromised. Finally, our proposed HDI-SDG pipeline provides a practical approach to combining real-world data insights with LLMs. This method mitigates the negative effects of long-tail distributions caused by healthcare providers’ interaction preferences in the training data, which was captured from real-world mHealth communication scenarios. The resulting model, LLMDF-EXP, is capable of generating comprehensive, practical, and humanized dietary feedback with optimal situational adaptability. We have open-sourced the model weights (https://huggingface.co/Rx-ZJU/LLMDF-EXP, accessed on 9 April 2026) to facilitate further research and encourage the deployment of LLMs in weight management dietary feedback scenarios.

## Figures and Tables

**Table 1:** Topics of dietary feedback.

No.	Topic	Highly Correlated Words in Topic	Example Dietary Feedback
1	Optimize meat and vegetable combinations	Vegetable, proportion, protein, advise, add, intake, arrange, ensure, meat, not enough, regular meal	The combination of food for lunch was generally good, high quality protein (chicken, tofu), vegetables, and staple food, but there was a bit of an imbalance in the amount of food, mainly in the amount of protein, so I would recommend choosing chicken for lunch and soya products in the evening, and it would be better to swap the soya products for another serving of vegetables for lunch.
2	Analyze the properties of food	Fat, prefer, quantity contained, easily, energy, vitamins, fat-loss, fiber, ingredient list, dietary mineral	Fruits suggested can be adjusted to low glycemic fruits, such as apple, dragon fruit, prune, small tomato, cucumber, grapefruit and white golden melon. For biscuits, please look at the ingredient list and the nutrition facts table, and do not choose those with high sugar and oil.
3	Feedback on breakfast pairings	Breakfast, nutrient-dense, milk, bread, soy milk, a cup of, promote, sandwich, frying, improve, low sugar	Breakfast bread is recommended to choose low-sugar and low-fat, in addition to breakfast is recommended to give priority to ensure that the intake of high-quality protein foods, with a glass of milk is very recommended!
4	Encouragement to maintain healthy eating habits	Continue, the whole meal, maintain, overall amount, order of meals, standard, keep it up, satiety at 70%, healthy, nice	Dinner was very good, light and low in oil, with a good mix of meat and vegetables, and a relatively standard mix of nutrients, so keep it up!
5	Increase intake of coarse grains	Satiety sensation, coarse grains, refined foods, carbohydrate, greasy, digestion, too high, corn, glycemic index	Glutinous Rice Rolls with Sweet Bean Flour is high in calories and is recommended not to eat or eat less. For staple food, it is better to choose mixed grains, next time you can choose corn, sweet potatoes, wholemeal bread, etc.

**Table 2:** Performance of LLMs in automatic evaluation.

Model	BLEU-1	ROUGE-L	METEOR	$P_{\mathrm{BERT}}$	$R_{\mathrm{BERT}}$	$F_{\mathrm{BERT}}$
GPT-4	0.084	0.112	0.170	0.583	0.693	0.633
GPT-3.5	0.105	0.136	0.177	0.590	0.701	0.641
ChatGLM3-6B	0.092	0.115	0.170	0.587	0.688	0.633
LLMDF	0.074	0.158	0.076	**0.705**	0.619	0.658
LLMDF-EXP	**0.294**	**0.213**	**0.191**	0.682	**0.708**	**0.694**

The best evaluation scores are presented in bold.

## Data Availability

The dietary records utilized for training the models can be obtained from the corresponding author upon reasonable request. The evaluation dataset, along with experts’ comments during the evaluation, is included in the Supplementary Materials.

## Supplementary Materials

The following supporting information can be downloaded at: https://www.mdpi.com/article/10.3390/bioengineering13040468/s1, Figure S1: Selection of the number of topics in LDA topic modeling; Figure S2: Statistical overview of the selected dietary scenarios for evaluation; Figure S3: The process of human evaluation; Prompt S1: The prompt used in our HDI-SDG pipeline; Prompt S2: The prompt template for training LLMDF and LLMDF-EXP; Prompt S3: The prompt used for generating dietary feedback during evaluation; Table S1: Means and standard deviations for survey items using a 5-point Likert scale; Table S2: Results of the Dunn’s Test for multiple comparisons, empathy; Table S3: Results of the Dunn’s Test for multiple comparisons, completeness; Table S4: Results of the Dunn’s Test for multiple comparisons, professionalism; Table S5: Results of the Dunn’s Test for multiple comparisons, usefulness; Table S6: Dietary feedback evaluation dataset; Table S7: Distribution of the three types of hallucination across models in the human evaluation experiment; Table S8: Examples of the three types of hallucination.
