# Supplementary material for: Using Large Language Models to Generate Dietary Feedback Similar to Human Experts in Weight Management: Experiments on Real-World Scenario Data
Source: Bioengineering (Basel). 2026 Apr 16;13(4):468. doi: 10.3390/bioengineering13040468 (PMC13113237; doi:10.3390/bioengineering13040468)
Supplement: Supplementary file 1 [file bioengineering-13-00468-s001.zip › bioengineering-4222712-supplementary.pdf]

Supplementary Material Figure S1

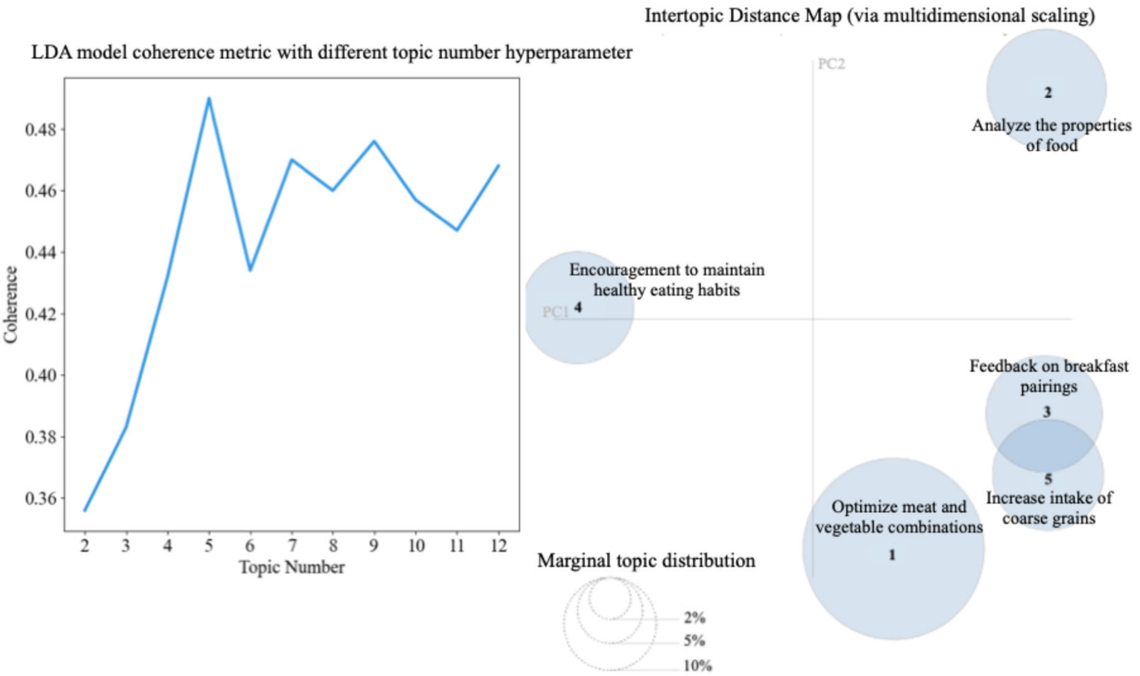

When implementing the LDA topic model, we calculated the coherence for different numbers of topics and used the LDAvis tool for topic visualization. We found that when the number of topics is five, the topic coherence is the highest, and LDAvis results in more dispersed and interpretable topics.

Supplementary Material Figure S2

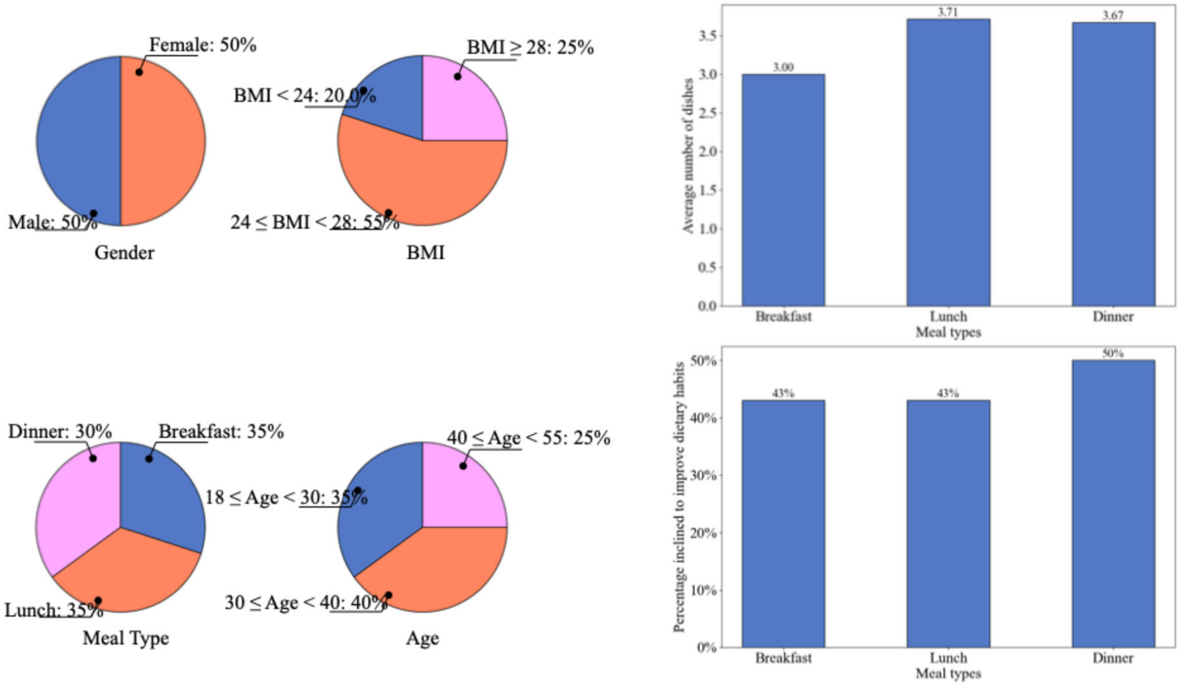

Statistical overview of the selected dietary scenarios for evaluation which covers the distribution of gender, overweight level, age, meal type and food combination preferences. Detailed information of the scenarios is presented in Supplementary Material Table S6.

Supplementary Material Figure S3

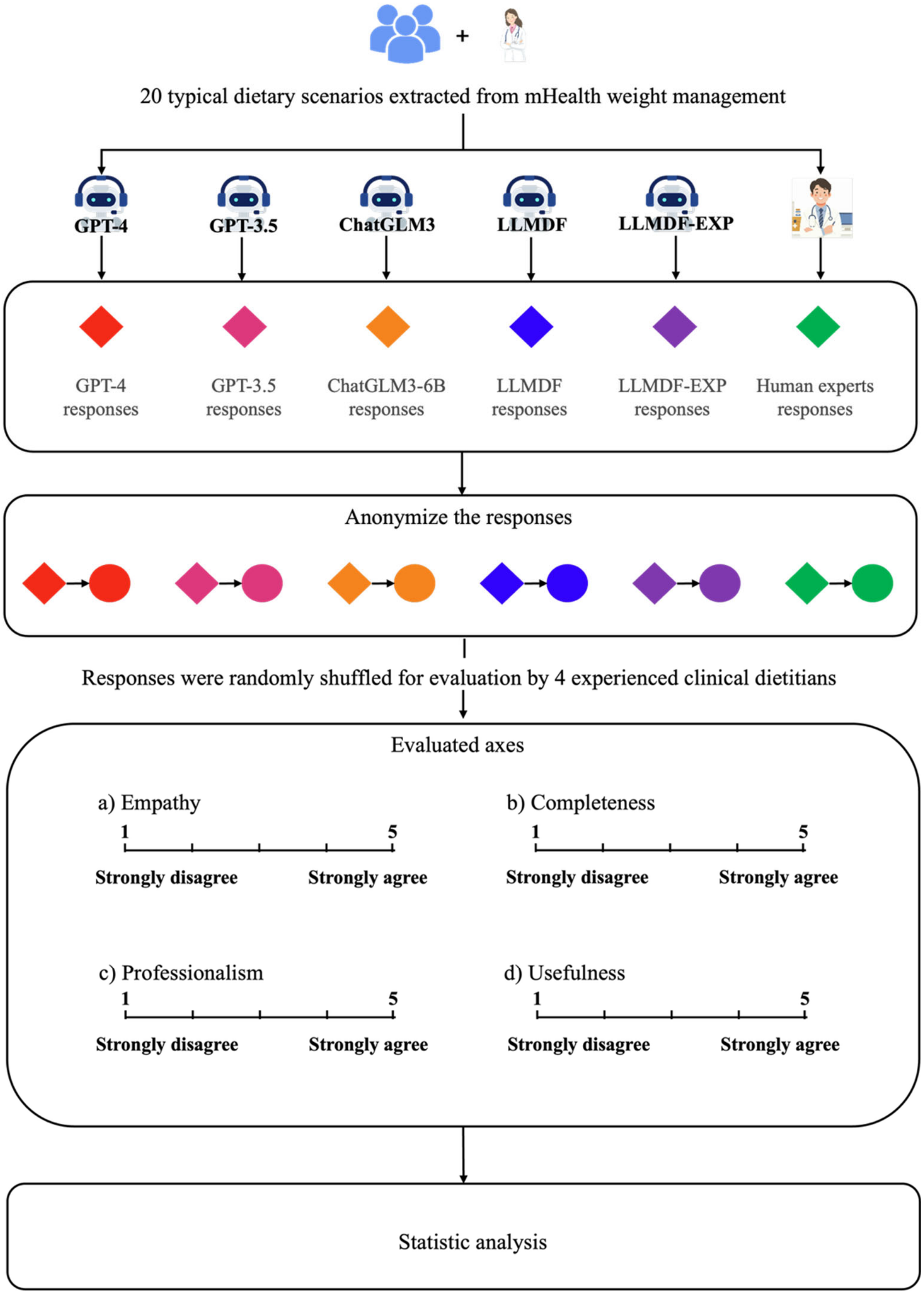

The process of human evaluation. We recruited four clinical dietitians with intermediate titles or higher. As shown in the picture, we mixed the responses generated by the five models to be evaluated with those generated by human experts. Then, we invited the dietitians to score the responses in four dimensions: empathy, completeness, professionalism, and usefulness, followed by statistical analysis. The statistic results are presented in Supplementary Material Tables S1-S5.

Supplementary Material Prompts.

Prompt S1:

Imagine you are a dietitian and you will receive dietary information from a client who is undergoing dietary management. Your task is to give the client feedback on the dietary information, and you need to provide insightful content to give the client dietary guidance to guide him towards a healthy lifestyle. \nYou can refer to organizing the dietary feedback from the following perspectives: analyzing the attributes of the food consumed by the client, providing healthy food choices, helping the client to develop a dietary pattern, encouraging the client to maintain healthy eating habits, and advising the client to modify his or her dietary habits.\n\nYou can learn expressions from some similar scenario examples below:\n\n<Example>[using input-output pairs to provide 5 shots in XML format]</Example>\n\nInput: [text of dietary and personal health information in XML format]

(e.g. 想象你是一位营养师，你将收到进行饮食管理的客户的饮食信息。你的任务是给客户提供饮食信息的反馈，你需要提供有见解性的内容给客户饮食指导，引导客户进行健康的生活方式。\n 你可以参考从以下角度组织你给客户的饮食反馈：分析客户摄入食物的属性、提供健康食物的选择、帮助客户制定饮食模式、鼓励客户维持健康的饮食习惯、建议客户修正饮食习惯。\n\n 你可以从下面的一些相似场景示例中学习表达方式：\n\n<Example>\nInput:<MealInformation><MealType>中餐</MealType><MealIntakeInformation>米饭，鸡腿，芹菜，内脏</MealIntakeInformation></MealInformation><PersonalHealthInformation><Gender>女</Gender><Age>24</Age><Height>165cm</Height><Weight>68.3kg</Weight><LaborIntensity>轻劳动强度</LaborIntensity></PersonalHealthInformation>\nOutput:<DietFeedback>午餐荤素搭配还不错，蔬菜比例低了些，建议以后蔬菜量可以有所增加。</DietFeedback>\n\nInput:<MealInformation><MealType>中餐</MealType><MealIntakeInformation>米饭，白菜，鸭肉，猪瘦肉</MealIntakeInformation></MealInformation><PersonalHealthInformation><Gender>女</Gender><Age>28</Age><Height>163cm</Height><Weight>68.9kg</Weight><LaborIntensity>中等劳动强度</LaborIntensity></PersonalHealthInformation>\nOutput:<DietFeedback>午餐很不错的，就是蔬菜可以再多一份，鸭肉记得去皮吃！</DietFeedback>\n\nInput:<MealInformation><MealType>晚餐</MealType><MealIntakeInformation>一份米饭，一份豆芽，一份玉米仔排</MealIntakeInformation></MealInformation><PersonalHealthInformation><Gender>男</Gender><Age>22</Age><Height>168cm</Height><Weight>

72.0kg</Weight><LaborIntensity>中等劳动强度</LaborIntensity></PersonalHealthInformation>\nOutput:<DietFeedback>晚餐饮食相对单一，建议午晚餐菜品多样化选择。</DietFeedback>\n\nInput:<MealInformation><MealType>晚餐</MealType><MealIntakeInformation>饺子 10 个，晚上再吃水果</MealIntakeInformation></MealInformation><PersonalHealthInformation><Gender>男</Gender><Age>50</Age><Height>166cm</Height><Weight>77.1kg</Weight><LaborIntensity>轻劳动强度</LaborIntensity></PersonalHealthInformation>\nOutput:<DietFeedback>有安排水果在餐后，这一点很棒，饺子以后还是建议能搭配蔬菜。</DietFeedback>\n\nInput:<MealInformation><MealType>晚餐</MealType><MealIntakeInformation>韭菜 南瓜 鱼 米饭</MealIntakeInformation></MealInformation><PersonalHealthInformation><Gender>女</Gender><Age>20</Age><Height>166cm</Height><Weight>67.3kg</Weight><LaborIntensity>中等劳动强度</LaborIntensity></PersonalHealthInformation>\nOutput:<DietFeedback>整体还行，南瓜建议算作主食，韭菜作为蔬菜可能量不是很够。</DietFeedback>\n</Example>\n\nInput: <MealInformation><MealType>晚餐</MealType><MealIntakeInformation>西红柿一个，生菜五片，黄瓜半个，荞麦面 1 包，鸡蛋 1 个，鸡肉肠 1 个</MealIntakeInformation></MealInformation><PersonalHealthInformation><Gender>男</Gender><Age>39</Age><Height>168cm</Height><Weight>75.3kg</Weight><LaborIntensity>轻劳动强度</LaborIntensity></PersonalHealthInformation>)

After receiving the synthetic dietary feedback, we generated a JSON file based on the following format: instruction: Imagine you are a dietitian and you will receive dietary information from a client who is undergoing dietary management. Your task is to give the client feedback on the dietary information, and you need to provide insightful content to give the client dietary guidance to guide him towards a healthy lifestyle. \nYou can refer to organizing the dietary feedback you give to your client from the following perspectives: analyzing the attributes of the food consumed by the client, providing healthy food choices, helping the client to develop a dietary pattern, encouraging the client to maintain healthy eating habits, and advising the client to modify his or her dietary habits; input: [text of dietary and personal health information in XML format]; output: [text of synthetic dietary feedback generated by GPT-3.5].

Prompt S2:

Instruction: Imagine you are a dietitian and you will receive dietary information from a client who is undergoing dietary management. Your task is to give the client feedback on the dietary information, and you need to provide

insightful content to give the client dietary guidance to guide him towards a healthy lifestyle. \nYou can refer to organizing the dietary feedback from the following perspectives: analyzing the attributes of the food consumed by the client, providing healthy food choices, helping the client to develop a dietary pattern, encouraging the client to maintain healthy eating habits, and advising the client to modify his or her dietary habits.

Input: [text of dietary and personal health information in XML format]

Output: [text of feedback for the dietary record in input using XML format]

Prompt S3:

Imagine you are a dietitian and you will receive dietary information from a client who is undergoing dietary management. Your task is to give the client feedback on the dietary information, and you need to provide insightful content to give the client dietary guidance to guide him towards a healthy lifestyle. \nYou can refer to organizing the dietary feedback from the following perspectives: analyzing the attributes of the food consumed by the client, providing healthy food choices, helping the client to develop a dietary pattern, encouraging the client to maintain healthy eating habits, and advising the client to modify his or her dietary habits. [text of dietary and personal health information in XML format]

(e.g. 想象你是一位营养师，你将收到进行饮食管理的客户的饮食信息。你的任务是给客户提供饮食信息的反馈，你需要提供有见解性的内容给客户饮食指导，引导客户进行健康的生活方式。\n 你可以参考从以下角度组织你给客户的饮食反馈：分析客户摄入食物的属性、提供健康食物的选择、帮助客户制定饮食模式、鼓励客户维持健康的饮食习惯、建议客户修正饮食习惯。<MealInformation><MealType>早餐</MealType><MealIntakeInformation>豆浆 1 碗;鸡蛋 1 中只;猪肉玉米胡萝卜糯米饭 200 克;</MealIntakeInformation></MealInformation><PersonalHealthInformation><Gender>男</Gender><Age>19</Age><Height>180cm</Height><Weight>84.0kg</Weight><Waistline>85cm</Waistline><LaborIntensity>中等劳动强度</LaborIntensity></PersonalHealthInformation>)

Supplementary Material Table S1

Means and standard deviations for survey items using a 5-point Likert scale (1-strongly disagree, 5-strongly agree).

	Experts	LLMDF	LLMDF-EXP	ChatGLM3-6B	GPT-3.5	GPT-4
**Empathy:** The feedback expresses appropriate empathy to the user.	3.63±0.51	3.73±0.45	3.79±0.44	3.79±0.41	3.85±0.36	3.90±0.30
**Completeness**: The feedback provides sufficiently comprehensive information.	3.81±0.50	3.05±0.79	3.99±0.43	4.11±0.63	4.22±0.45	4.26±0.49
**Professionalism**: The feedback is nutritionally accurate, tailored and soundly responses to the user.	3.93±0.77	2.86±0.83	3.83±0.57	3.41±0.72	3.76±0.58	3.63±0.66
**Usefulness**: I can use it as a template to write my feedback to this scene.	3.70±0.78	2.73±0.77	3.79±0.72	3.03±0.57	3.50±0.72	3.30±0.68

Supplementary Material TableS S2-S5

Results of the Dunn's Test for Multiple Comparisons.

Supplementary Material Table S2 Empathy.
KruskalResult (statistic=19.86, p-value = 0.001)

	Experts	LLMDF	LLMDF-EXP	ChatGLM3-6B	GPT-3.5	GPT-4
**Experts**	1	0.173	0.023	0.021	0.001	<0.001
**LLMDF**	0.173	1	0.366	0.345	0.059	0.008
**LLMDF-EXP**	0.023	0.366	1	0.967	0.324	0.082
**ChatGLM3-6B**	0.021	0.435	0.967	1	0.345	0.089
**GPT-3.5**	0.001	0.059	0.324	0.345	1	0.450
**GPT-4**	<0.001	0.008	0.082	0.089	0.450	1

Supplementary Material Table S3 Completeness.
KruskalResult (statistic=150.89, p-value < 0.001)

	Experts	LLMDF	LLMDF-EXP	ChatGLM3-6B	GPT-3.5	GPT-4
**Experts**	1	<0.001	0.084	0.003	<0.001	<0.001
**LLMDF**	<0.001	1	<0.001	<0.001	<0.001	<0.001
**LLMDF-EXP**	0.084	<0.001	1	0.218	0.017	0.005
**ChatGLM3-6B**	0.003	<0.001	0.218	1	0.244	0.118
**GPT-3.5**	<0.001	<0.001	0.017	0.244	1	0.691
**GPT-4**	<0.001	<0.001	0.005	0.118	0.691	1

Supplementary Material Table S4 Professionalism.
KruskalResult (statistic=97.99, p-value < 0.001)

	Experts	LLMDF	LLMDF-EXP	ChatGLM3-6B	GPT-3.5	GPT-4
**Experts**	1	<0.001	0.510	<0.001	0.441	0.032
**LLMDF**	<0.001	1	<0.001	<0.001	<0.001	<0.001
**LLMDF-EXP**	0.510	<0.001	1	<0.001	0.912	0.138
**ChatGLM3-6B**	<0.001	<0.001	<0.001	1	0.001	0.063
**GPT-3.5**	0.441	<0.001	0.912	0.001	1	0.170
**GPT-4**	0.032	<0.001	0.138	0.063	0.170	1

Supplementary Material Table S5 Usefulness.
KruskalResult (statistic=101.29, p-value < 0.001)

	Experts	LLMDF	LLMDF-EXP	ChatGLM3-6B	GPT-3.5	GPT-4
**Experts**	1	<0.001	0.498	<0.001	0.131	0.002
**LLMDF**	<0.001	1	<0.001	0.040	<0.001	<0.001
**LLMDF-EXP**	0.498	<0.001	1	<0.001	0.029	<0.001
**ChatGLM3-6B**	<0.001	0.040	<0.001	1	<0.001	0.016
**GPT-3.5**	0.131	<0.001	0.029	<0.001	1	0.126
**GPT-4**	0.002	<0.001	<0.001	0.016	0.126	1

Supplementary Material Table S6 Dietary Feedback Evaluation Dataset.

Model	Scene	Feedback	Raters' Comments
GPT-4	Personal Health Information<br><br>Gender: Male<br><br>Age: 24<br><br>Height: 173cm<br><br>Weight: 83.0kg<br><br>Labor intensity: Light labor intensity<br><br><br>Dietary Information<br><br>Meal type: Breakfast<br><br>Food intake: Spicy salty egg yolk flavored noodles imported from Vietnam 100g; Small dumplings 100g.	Dear Client, I have carefully read your dietary information and I am going to provide you with some personalized nutritional advice.<br><br>First of all, in terms of food intake for breakfast, spicy salted egg yolk flavored noodles and small wontons may meet your needs in terms of taste and convenience, but they do not have many core nutrients (e.g., proteins, etc.). Moreover, they are ready-to-eat foods with high sodium content, and prolonged consumption of these two food items may increase the risk of blood pressure and other risks.<br><br>For your age, height and weight, as well as your labor intensity, it is recommended that you try to consume more protein and complex carbohydrates in your breakfast, such as oatmeal, whole grain bread, cheese and eggs. These foods provide high quality protein and enough energy to keep you going throughout the day.<br><br>Additionally, I recommend developing a scientific eating pattern within your means. For example, try incorporating cheese and eggs into your breakfast, or opt for a green breakfast that includes fresh fruits and vegetables. Adhering to healthy eating habits will not only help you maintain a healthy body, but will also add variety and vitality to your life.<br><br>Overall, I hope you find these suggestions helpful in adjusting the composition of your breakfast, increasing your intake of high-quality proteins and complex carbohydrates, and making healthier food choices based on your specific situation to ensure that the quantity and quality of your life needs are as high as possible in the future. I look forward to working with you to change your eating habits and pursue a healthier lifestyle.	Rater1: Giving overly lengthy dietary feedback.<br><br>Rater2: Dietary feedback is too lengthy; suggestions could be more practical.<br><br>Rater3: None<br><br>Rater4: None
GPT-3.5	Personal Health Information<br><br>Gender: Male<br><br>Age: 24<br><br>Height: 173cm<br><br>Weight: 83.0kg	Based on the dietary and personal health information you have provided, I believe there are some problems with your breakfast intake. Spicy salted egg yolk flavored noodles and small wontons, although tasty, are high in calories, fats, and salt, and their long-term consumption may have adverse effects on your health.<br><br>To help you maintain a healthy lifestyle, I suggest you add some healthy food choices to your breakfast, such as fruits, whole grain breads, and vegetables. These foods are rich in fiber, vitamins,	Rater1: The dietary feedback is not friendly to understand.<br><br>Rater2: Dietary feedback is too lengthy.<br><br>Rater3: None<br><br>Rater4: None

	Labor intensity: Light labor intensity<br><br>Dietary Information<br><br>Meal type: Breakfast<br><br>Food intake: Spicy salty egg yolk flavored noodles imported from Vietnam 100g; Small dumplings 100g.	and minerals, which help boost satiety, manage weight, and provide well-rounded nutrition.<br><br>When developing your eating pattern, you may want to consider pairing each meal with a sensible mix of proteins, carbohydrates, and fats to maintain a proper calorie intake and avoid favoritism and overeating.<br><br>It is important to maintain a healthy diet. I encourage you to consume healthy foods such as fresh fruits and vegetables, lean meats, fish, etc. It is also important to reduce your intake of sugar, fats and salt, and to drink plenty of water.<br><br>Lastly, I recommend that you revise your dietary habits and gradually reduce your intake of high-calorie, high-fat and high-salt foods to guide yourself to develop healthy eating habits that will improve your health and enhance your quality of life. I hope my advice is helpful to you, and I wish you good health and a happy life! If you have any questions or need further guidance, please feel free to contact me.	
ChatGLM3-6B	Personal Health Information<br><br>Gender: Male<br><br>Age: 24<br><br>Height: 173cm<br><br>Weight: 83.0kg<br><br>Labor intensity: Light labor intensity<br><br>Dietary Information<br><br>Meal type: Breakfast<br><br>Food intake: Spicy salty egg yolk flavored noodles imported from Vietnam 100g; Small dumplings 100g.	Dear Client, Based on the dietary information you have provided, I have noticed that you consume mostly pasta and meat for breakfast, which tends to lead to blood sugar fluctuations and excessive calorie intake. To maintain a healthy diet, I recommend that you increase your intake of vegetables and fruits at breakfast to balance your nutrition and increase satiety.<br><br>When making healthy food choices, opt for foods that are high in fiber, low in sugar, and low in fat, such as whole grain bread, oatmeal, fresh fruits and vegetables. In addition, avoid excessive intake of processed foods, beverages high in sugar, and high-fat meats such as fried foods and fatty meats.<br><br>For your dietary pattern, it is recommended that you adopt the pattern of "rich breakfast, balanced lunch and small dinner". Breakfast is a good way to get enough nutrients to keep you energized, lunch is a good way to get the right amount of carbohydrates and proteins to provide you with enough energy, and dinner is a good way to cut down on your intake to avoid too many calories.<br><br>I encourage you to maintain a healthy diet, exercise regularly and keep a good routine to stay fit. In the meantime, please feel	Rater1: Feedback given is too lengthy and more like a bot-generated response.<br><br>Rater2: Dietary feedback is too lengthy.<br><br>Rater3: None<br><br>Rater4: None

		free to consult me if you have any questions or need further dietary advice.	
LLMDF	Personal Health Information<br><br>Gender: Male<br><br>Age: 24<br><br>Height: 173cm<br><br>Weight: 83.0kg<br><br>Labor intensity: Light labor intensity<br><br><br>Dietary Information<br><br>Meal type: Breakfast<br><br>Food intake: Spicy salty egg yolk flavored noodles imported from Vietnam 100g; Small dumplings 100g.	It's quite good to have noodles and wontons for breakfast, but there's no vegetable intake. It's recommended to add vegetables, which will make the nutrition more balanced.	Rater1: Too simple and not tailored.<br><br>Rater2: Too simple and not comprehensive<br><br>Rater3: None<br><br>Rater4: None
LLMDF-EXP	Personal Health Information<br><br>Gender: Male<br><br>Age: 24<br><br>Height: 173cm<br><br>Weight: 83.0kg<br><br>Labor intensity: Light labor intensity<br><br><br>Dietary Information<br><br>Meal type: Breakfast<br><br>Food intake: Spicy salty egg yolk flavored noodles imported from Vietnam 100g; Small dumplings 100g.	There is still room for improvement in breakfast combinations. Spicy salted egg yolk flavored noodles are high in salt and oil; it is recommended to reduce the intake or choose other low-salt, low-oil breakfast options. You can try adding a serving of fruit or vegetables to increase dietary fiber intake. Additionally, if you have a habit of eating noodles, consider making them yourself to control the amount of salt and oil used for a healthier breakfast.	Rater1: Dietary feedback given is professional.<br><br>Rater2: The advice given is relatively practical and professional.<br><br>Rater3: None<br><br>Rater4: None
Experts	Personal Health Information	The noodles for breakfast are high-calorie, high-carb, and high-fat foods, which are not conducive to weight control and are not	Rater1: Concise.

	Gender: Male<br><br>Age: 24<br><br>Height: 173cm<br><br>Weight: 83.0kg<br><br>Labor intensity: Light labor intensity<br><br><br>Dietary Information<br><br>Meal type: Breakfast<br><br>Food intake: Spicy salty egg yolk flavored noodles imported from Vietnam 100g; Small dumplings 100g.	recommended. If you do eat it, you should control the amount. For breakfast, it is recommended to follow the formula of low-oil staple food + high-quality protein + fruits and vegetables.	Rater2: The advice given was relatively practical and professional, but the suggestions could have been more specific.<br><br>Rater3: None<br><br>Rater4: None
GPT-4	Personal Health Information<br><br>Gender: Female<br><br>Age: 19<br><br>Height: 166cm<br><br>Weight: 71.5kg<br><br>Waistline: 87.0cm<br><br>Labor intensity: Medium labor intensity<br><br><br>Dietary Information<br><br>Meal type: Breakfast<br><br>Food intake: Millet pumpkin porridge, fired leek dumplings, egg	From the information you have provided, your breakfast choices are quite balanced. The millet and pumpkin porridge is a good source of carbohydrates and is also rich in vitamins and minerals, especially the pumpkin, which is rich in beta-carotene and dietary fiber, which are good for digestive health and antioxidants. Leek dumplings are a great source of protein and vegetables to go with them, as well as providing some fat. Eggs are an excellent source of protein, containing all the essential amino acids, as well as being rich in vitamins A, D, E and B vitamins.<br><br>However, I would caution you that foods containing too much fat such as leek boxes may increase total energy intake, especially if you are aiming to lose weight. Consider reducing it in moderation or cooking it by baking or steaming. You can also try adding some low-sugar fruits such as cherries or blueberries to increase your dietary fiber and nutrient intake.<br><br>Based on your height and weight, your BMI is 26, which is outside the healthy range. The standard BMI for Asians is 18.5-22.9 as normal. Your BMI is too high and you are overweight. This requires more attention to a balanced diet and increased exercise. Moderate labor intensity work also requires adequate energy support, but energy intake should be properly controlled to avoid excess.<br><br>You are encouraged to maintain this healthy diet but reduce your intake of fats and sugars to some extent and increase the	Rater1: None<br><br>Rater2: Dietary feedback is too lengthy.<br><br>Rater3: The BMI standard for Asians is 18.5-22.9, and the Chinese standard is 18.5-23.9.<br><br>Rater4: None

		proportion of vegetables and lean meat. If you can combine this with regular exercise and a moderate diet, I believe you can return to a healthy state very quickly.	
GPT-3.5	Personal Health Information<br><br>Gender: Female<br><br>Age: 19<br><br>Height: 166cm<br><br>Weight: 71.5kg<br><br>Waistline: 87.0cm<br><br>Labor intensity: Medium labor intensity<br><br><br>Dietary Information<br><br>Meal type: Breakfast<br><br>Food intake: Millet pumpkin porridge, fired leek dumplings, egg	Based on the information you provided about your breakfast, I can see that you have a relatively balanced breakfast mix of staples, vegetables and protein sources, but there is some room for improvement. Firstly, it is recommended that you increase your fruit or vegetable intake to ensure that you are getting enough vitamins and minerals. Secondly, millet pumpkin porridge and eggs are good choices, but leek dumplings may contain more fat and calories, so it is recommended to eat them in moderation or choose other healthier vegetable options. In addition, based on your personal health information, it is recommended to control the amount of food intake at each meal, especially for staple foods and fats. Try increasing the portion of fruits and vegetables, choosing low-fat and low-sugar foods, and maintaining a moderate amount of exercise and water intake to help control your weight and maintain good health. We hope that you will be able to maintain your health and energy levels by adjusting your diet and establishing a healthy lifestyle. Please feel free to contact me if you have any questions or need further guidance. I wish you good health!	Rater1: The dietary feedback is not friendly to understand.<br><br>Rater2: The advice given is relatively practical and professional, but could be expressed in a more humane way.<br><br>Rater3: None<br><br>Rater4: None
ChatGLM3-6B	Personal Health Information<br><br>Gender: Female<br><br>Age: 19<br><br>Height: 166cm<br><br>Weight: 71.5kg<br><br>Waistline: 87.0cm<br><br>Labor intensity: Medium labor intensity<br><br><br>Dietary Information<br><br>Meal type: Breakfast<br><br>Food intake: Millet pumpkin porridge, fired leek dumplings, egg	Dear Client, Based on the dietary information you have provided, I have noticed that you are consuming more high-calorie foods such as millet pumpkin porridge and leek dumplings for breakfast, as well as fewer protein sources such as eggs. This dietary pattern may not be meeting the nutritional requirements you need, especially protein and fiber.<br><br>To ensure your health, I recommend that you increase your protein and fiber intake at future breakfasts. You can choose whole wheat bread or whole wheat toast instead of leek dumplings, and fruit or nuts instead of millet pumpkin porridge. In addition, eggs are a good source of protein, but consider increasing your intake of other protein foods such as lean meat, fish and tofu.<br><br>In addition, I have noticed that your diet lacks sufficient vegetables and fruits, which may result in inadequate vitamin and mineral intake. Therefore, I recommend you to increase the intake of vegetables and fruits in your meals, such as you can include fresh fruits or vegetable juices in your breakfast, or	Rater1: Feedback given is too lengthy and more like a bot-generated response.<br><br>Rater2: Dietary feedback is too lengthy and should be more concise.<br><br>Rater3: There is a professional error: millet pumpkin porridge is not a high-calorie food. **(Knowledge error)**<br><br>Rater4: None

		consume more vegetable salads in your lunch and dinner.<br><br>Finally, I recommend that you maintain a well-balanced diet, avoid over-consumption of high-calorie foods, and maintain a moderate level of physical activity to help maintain your healthy weight. If you have any questions or need further dietary advice, please feel free to contact me.	
LLMDF	Personal Health Information<br><br>Gender: Female<br><br>Age: 19<br><br>Height: 166cm<br><br>Weight: 71.5kg<br><br>Waistline: 87.0cm<br><br>Labor intensity: Medium labor intensity<br><br>Dietary Information<br><br>Meal type: Breakfast<br><br>Food intake: Millet pumpkin porridge, fired leek dumplings, egg	You have eaten a very healthy meal, please keep it up!<br>The vegetable ratio could be increased a bit more.	Rater1: Too simple and not tailored.<br><br>Rater2: Too simple and not comprehensive<br><br>Rater3: The feedback is too simple and this breakfast has room for improvement for overweight people, not very healthy<br><br>Rater4: None
LLMDF-EXP	Personal Health Information<br><br>Gender: Female<br><br>Age: 19<br><br>Height: 166cm<br><br>Weight: 71.5kg<br><br>Waistline: 87.0cm<br><br>Labor intensity: Medium labor intensity<br><br>Dietary Information	Millet and pumpkin porridge for breakfast is a good choice as it is rich in fiber and vitamins, which helps digestion and increases satiety. Leek dumpling is a noodle dish, although it tastes good, it is high in calories, so it is recommended to reduce the intake or choose other low-calorie pasta as an alternative. Eggs are a good quality source of protein, rich in various vitamins and minerals, and can continue to be consumed. However, considering your weight and waistline, it is recommended that you control your overall calorie intake, moderately reduce the intake of staple food and pasta, and increase the intake of vegetables and fruits to help control your weight and waistline.	Rater1: Dietary feedback given is professional.<br><br>Rater2: The advice given is relatively practical and professional.<br><br>Rater3: None<br><br>Rater4: None

	Meal type: Breakfast<br><br>Food intake: Millet pumpkin porridge, fired leek dumplings, egg		
Experts	Personal Health Information<br><br>Gender: Female<br><br>Age: 19<br><br>Height: 166cm<br><br>Weight: 71.5kg<br><br>Waistline: 87.0cm<br><br>Labor intensity: Medium labor intensity<br><br>Dietary Information<br><br>Meal type: Breakfast<br><br>Food intake: Millet pumpkin porridge, fired leek dumplings, egg	The breakfast combination is basically reasonable, with staple food and high-quality protein, but the fired leek dumpling, has a relatively high fat content. Fried staple foods are not conducive to weight control, so it is recommended to reduce intake.	Rater1: Dietary feedback given is professional.<br><br>Rater2: The advice given was relatively practical and professional.<br><br>Rater3: None<br><br>Rater4: None
GPT-4	Personal Health Information<br><br>Gender: Male<br><br>Age: 29<br><br>Height: 174cm<br><br>Weight: 74.6kg<br><br>Labor intensity: Light labor intensity<br><br>Dietary Information<br><br>Meal type: Breakfast<br><br>Food intake: One egg, one vegetable bun, one soy milk (less sugar)	Based on the information you provided about your breakfast, your breakfast consisted of eggs, vegetable packets, and soymilk, constituting a more balanced intake of carbohydrates, proteins, and vitamins. Eggs are rich in high-quality protein, vegetable buns provide essential carbohydrates, and soy milk gives you plant-based protein and micronutrients.<br><br>However, the fat content of this breakfast meal may be on the low side. Given your age, gender, and weight, your basal metabolic rate is quite high, and it may be helpful to increase your intake of healthy fats, such as by adding some milk or whole wheat bread, which can add calories as well as keep you full.<br><br>Your work intensity is classified as light labor, so your calorie consumption will not be high. As breakfast is the start of the day, you can make small adjustments, such as replacing a vegetable bun with whole wheat bread and adding a fruit to supplement your vitamin intake.<br><br>Remember, a well-balanced, nutrient-dense diet and regular	Rater1: The feedback given is too long and more like a bot-generated response, with inconsistencies in presentation. **(Inconsistency)**<br><br>Rater2: None<br><br>Rater3: There are inconsistencies. **(Inconsistency)**<br><br>Rater4: None

		exercise are your keys to maintaining a healthy weight and boosting your body's immunity. If necessary, consider increasing your intake of fresh vegetables and fruits at different times of the day as appropriate. Maintaining a varied diet will help you consume a more complete range of nutrients. I hope these suggestions are helpful and look forward to better management of your weight and health!	
GPT-3.5	Personal Health Information<br><br>Gender: Male<br><br>Age: 29<br><br>Height: 174cm<br><br>Weight: 74.6kg<br><br>Labor intensity: Light labor intensity<br><br>Dietary Information<br><br>Meal type: Breakfast<br><br>Food intake: One egg, one vegetable bun, one soy milk (less sugar)	Based on the breakfast information you provided and your personal health information, I can give you some suggestions. First of all, your breakfast contains protein food (eggs), staple food (vegetable packet) and legumes (soy milk), which is a relatively balanced breakfast combination, but I would suggest that you could add some vegetables and fruits to increase your intake of dietary fiber and vitamins.<br><br>Given that you are a male with high muscle mass and light labor intensity, it is recommended that you increase your carbohydrate and fat intake moderately to meet your energy needs. You may want to consider adding some whole grains (e.g., whole wheat bread, oats, etc.) and healthy fats (e.g., nuts, avocados, etc.).<br><br>In addition, given your health condition, it is recommended that you maintain a regular meal schedule and a moderately varied diet that includes a full range of proteins, minerals and vitamins. It is also recommended that you engage in physical activity to stay in good physical condition.<br><br>Finally, to maintain healthy eating habits, you can consume less processed foods, sugar and salt in your daily diet, more fruits, vegetables, whole grains and other foods rich in fiber, fruit in the meal portion and variety and more rhythmic diet. We look forward to your ability to live a healthier life by adjusting your eating habits. Please feel free to contact me if you have additional questions or need more help. I wish you the best of health!	Rater1: Feedback given is too lengthy.<br><br>Rater2: Dietary feedback could be simplified a bit.<br><br>Rater3: Feedback given is too lengthy.<br><br>Rater4: None
ChatGLM3-6B	Personal Health Information<br><br>Gender: Male<br><br>Age: 29<br><br>Height: 174cm<br><br>Weight: 74.6kg<br><br>Labor intensity: Light labor intensity	Dear Client.<br><br>According to the dietary information you provided, your breakfast intake is mainly based on eggs and soy products, which are a good source of protein. However, the intake of vegetable buns and soymilk is high, which you are advised to reduce appropriately to maintain a balanced nutritional profile. In addition, your dietary intake of sugar is low, which is a positive sign.	Rater1: Feedback given is too lengthy.<br><br>Rater2: Misunderstanding of the content of dietary intake **(Misunderstanding)**<br><br>Rater3: None<br><br>Rater4: None

	Dietary Information<br><br>Meal type: Breakfast<br><br>Food intake: One egg, one vegetable bun, one soy milk (less sugar)	From a health perspective, it is recommended that you add some fruits and vegetables to your breakfast to boost your vitamin and mineral intake. For example, you could replace one serving of soymilk with one serving of soymilk with fruit, or add some fresh vegetables to your vegetable packet.<br><br>For your personal health information, your height and weight indicate that you have some muscle mass but are heavier. It is recommended that you maintain a balanced diet and increase your physical activity to achieve better health.<br><br>Overall, your diet is already good and you just need to make some appropriate adjustments to be healthier. I hope these suggestions are helpful and if you have any questions, please feel free to ask me.<br><br>Best wishes for your health!<br>Your Dietitian	
LLMDF	Personal Health Information<br><br>Gender: Male<br><br>Age: 29<br><br>Height: 174cm<br><br>Weight: 74.6kg<br><br>Labor intensity: Light labor intensity<br><br>Dietary Information<br><br>Meal type: Breakfast<br><br>Food intake: One egg, one vegetable bun, one soy milk (less sugar)	Breakfast was good overall, soy milk for milk would be more recommended.	Rater1: Not tailored.<br><br>Rater2: The recommendations are oversimplified and misguided. **(Knowledge error)**<br><br>Rater3: Milk and soya are in the same tier of food in the dietary pagoda and both are highly recommended, not more so milk.<br><br>Rater4: None
LLMDF-EXP	Personal Health Information<br><br>Gender: Male<br><br>Age: 29<br><br>Height: 174cm	The breakfast choices are not bad, with eggs and soymilk to provide protein and nutrients, and vegetable buns to provide some energy, but it is recommended to control the intake of oil and salt by choosing vegetable buns with less oil and salt.	Rater1: Dietary feedback given is professional.<br><br>Rater2: The advice given is relatively practical and professional.<br><br>Rater3: None

	Weight: 74.6kg<br><br>Labor intensity: Light labor intensity<br><br>Dietary Information<br><br>Meal type: Breakfast<br><br>Food intake: One egg, one vegetable bun, one soy milk (less sugar)		Rater4: None
Experts	Personal Health Information<br><br>Gender: Male<br><br>Age: 29<br><br>Height: 174cm<br><br>Weight: 74.6kg<br><br>Labor intensity: Light labor intensity<br><br>Dietary Information<br><br>Meal type: Breakfast<br><br>Food intake: One egg, one vegetable bun, one soy milk (less sugar)	Breakfast was well put together, with a balance of staples, vegetables, animal foods, and soy products, and the choice of beverages followed the principle of less sugar.	Rater1: Concise.<br><br>Rater2: None<br><br>Rater3: None<br><br>Rater4: None
GPT-4	Personal Health Information<br><br>Gender: Female<br><br>Age: 38<br><br>Height: 165cm<br><br>Weight: 76.8kg<br><br>Labor intensity: Medium labor intensity<br><br>Dietary Information	Based on what you provided for breakfast and your personal health information, I would give the following suggestions for optimizing your diet:<br><br>First of all, looking at your breakfast plate of scrambled eggs, scrambled eggs is a good place to start as it contains eggs from a protein source and rice from a carbohydrate source. However limiting the amount of fat in your meal is also necessary to avoid excessive calorie intake.<br><br>A healthier option may be to include a variety of colorful vegetables such as carrots, peas or red peppers. These can add dietary fiber for healthy digestion and provide a way to delay hunger pangs, making you less likely to feel hungry before lunch.	Rater1: Feedback given is too lengthy.<br><br>Rater2: Feedback given is too lengthy and more like a bot-generated response.<br><br>Rater3: It's already at the obese level and should be clearly stated that.<br><br>Rater4: None

	Meal type: Breakfast<br><br>Food intake: One plate of egg fried rice	In addition, we can also try to change or adjust your eating pattern, breakfast can be varied, for example, you can choose boiled eggs, whole grain bread with a cup of unsweetened yogurt or juice, to take in a diversity of nutrients.<br><br>Meanwhile, I hope you can maintain this healthy eating habit. It is important to ensure the quality of your breakfast, as it will keep you in good spirits in the early morning hours and provide you with the energy you need throughout the day.<br><br>Furthermore, due to your age, weight, height and work intensity, I realize that you may be slightly overweight. You may need to pay more attention to calorie control and physical activity. Eating a diet that is as low in sugar, salt and oil but rich in protein and dietary fiber as possible, combined with proper exercise, will help reduce your weight and is vital to your health.<br><br>Above, these are my dietary guidelines for you. Come along and make a nutritional change that will bring about a healthier life!	
GPT-3.5	Personal Health Information<br><br>Gender: Female<br><br>Age: 38<br><br>Height: 165cm<br><br>Weight: 76.8kg<br><br>Labor intensity: Medium labor intensity<br><br>Dietary Information<br><br>Meal type: Breakfast<br><br>Food intake: One plate of egg fried rice	Based on the breakfast information you provided, you ingested a plate of scrambled eggs. First, let's analyze the properties of this food. Egg fried rice is a food that contains protein and carbohydrates, with the right amount of protein helping to maintain muscle health and carbohydrates providing the main source of energy. However, this food can be overly greasy and contains high levels of calories and fats, which may lead to weight gain as well as health problems if consumed in excess over a long period of time.<br><br>For your personal health situation, as a woman, 38 years old and moderately labor-intensive, I would suggest that you make healthier food choices for breakfast. You may want to consider pairing a serving of fruit with whole grain bread to increase your dietary fiber intake and improve satiety. If you are looking to maintain or lose weight, you may choose to reduce your intake of foods that are higher in fat and increase your servings of vegetables and protein.<br><br>In addition, it is recommended that you organize your daily meals, including three meals and a moderate amount of snacks, to maintain a varied diet and ensure a balanced intake of nutrients. Moderate exercise is also important to help you maintain a healthy weight and physical condition.<br><br>Finally, I hope you realize how important your diet is to your	Rater1: The dietary feedback is not friendly to understand.<br><br>Rater2: Feedback given is too lengthy.<br><br>Rater3: Feedback given is too lengthy.<br><br>Rater4: None

		health, and that adhering to a healthy diet will contribute to your health and quality of life. If you have any questions about your diet or need further guidance, please feel free to contact me. Best wishes for your health!	
ChatGLM3-6B	Personal Health Information<br><br>Gender: Female<br><br>Age: 38<br><br>Height: 165cm<br><br>Weight: 76.8kg<br><br>Labor intensity: Medium labor intensity<br><br><br>Dietary Information<br><br>Meal type: Breakfast<br><br>Food intake: One plate of egg fried rice	Dear Customer.<br><br>Based on the dietary information you provided, you ingested a plate of scrambled eggs for breakfast, a food that is high in fat and salt and could potentially impact your health. As a dietitian, I recommend that you focus more on a healthy balance of nutrients in your future breakfast choices.<br><br>For starters, you can try increasing your intake of vegetables and fruits to boost your dietary fiber and vitamin intake. For example, you may choose to eat a serving of fruit or a vegetable salad to complement your breakfast. Next, you may want to consider choosing whole grains, such as whole wheat bread or brown rice, which provide a longer feeling of fullness and help control your appetite.<br><br>In addition, based on your personal health information, you have a moderate labor intensity, so you need more energy to support your daily activities. Choosing nutrient-dense foods can help provide you with enough energy while also helping to keep you healthy.<br><br>Finally, I recommend that you develop a healthy eating pattern to help you achieve a healthy lifestyle. You may consider following the "grains, meat, eggs, vegetables and fruits" diet to ensure that you have a balanced diet.<br><br>I hope you find these suggestions helpful and please feel free to contact me if you have any questions or need further guidance. I wish you good health!<br><br>Your Dietitian	Rater1: Feedback given is too lengthy and more like a bot-generated response.<br><br>Rater2: Feedback given is too lengthy.<br><br>Rater3: The dietary advice is not very practical, and the vegetable salad: raw vegetables may not be very appetizing for a middle-aged obese woman.<br><br>Rater4: None
LLMDF	Personal Health Information<br><br>Gender: Female<br><br>Age: 38<br><br>Height: 165cm	Scrambled eggs are a good choice, but it is recommended to add a serving of vegetables, such as cucumbers and carrots.	Rater1: None<br><br>Rater2: Recommendations are misguided. **(Knowledge error)**<br><br>Rater3: None<br><br>Rater4: None

	Weight: 76.8kg<br><br>Labor intensity: Medium labor intensity<br><br><br>Dietary Information<br><br>Meal type: Breakfast<br><br>Food intake: One plate of egg fried rice		
LLMDF-EXP	Personal Health Information<br><br>Gender: Female<br><br>Age: 38<br><br>Height: 165cm<br><br>Weight: 76.8kg<br><br>Labor intensity: Medium labor intensity<br><br><br>Dietary Information<br><br>Meal type: Breakfast<br><br>Food intake: One plate of egg fried rice	The egg scramble for breakfast consumed more fats and carbohydrates, so we suggest that next time you try to choose a healthier breakfast option, such as whole wheat bread with eggs and vegetables.	Rater1: None<br><br>Rater2: Stiff expression.<br><br>Rater3: The tone is a little stiff.<br><br>Rater4: None
Experts	Personal Health Information<br><br>Gender: Female<br><br>Age: 38<br><br>Height: 165cm<br><br>Weight: 76.8kg<br><br>Labor intensity: Medium labor intensity<br><br><br>Dietary Information<br><br>Meal type: Breakfast	Breakfast diet is mono-structured, with fried rice usually having more rice, more oil, and higher overall energy. Current weight is in the obese state. It is recommended to choose less egg fried rice or control the portion size, while increasing the proportion of vegetables in breakfast. In addition, you can also optimize the breakfast structure with soy milk and skimmed milk.	Rater1: Dietary feedback given is professional.<br><br>Rater2: The advice given is relatively practical and professional, but could be expressed in a more humane way<br><br>Rater3: None<br><br>Rater4: None

	Food intake: One plate of egg fried rice		
GPT-4	Personal Health Information<br><br>Gender: Female<br><br>Age: 37<br><br>Height: 160cm<br><br>Weight: 68.85kg<br><br>Labor intensity: Light labor intensity<br><br>Dietary Information<br><br>Meal type: Breakfast<br><br>Food intake: Half bowl of porridge, 1 egg, 1 cucumber, 2 pieces of watermelon, 250ml of skimmed milk	Based on the information you have provided, I have performed some nutritional analysis for you.<br><br>First of all, your breakfast food items are basically balanced, with protein-rich eggs, and vegetables and fruits containing minerals and dietary fiber, which have a positive impact on your health. However, you also need to consider adding some grains such as semolina and brown rice to your breakfast, which provide energy and help you stay energized early in the morning. Skimmed milk, on the other hand, is a good source of protein and calcium, which is suitable for keeping your bones healthy.<br><br>Immediately following this, I notice that your weight is slightly above the normal range for BMI. Although your labor intensity is in the light labor category, in order to better maintain your health and control your weight, I would recommend that you make more choices regarding the structure of your diet that is low in sugar and calories. Try choosing more vegetables, lean meats, and whole grains while reducing your sugar and fat intake.<br><br>Further, you can try eating regularly, which can help your body maintain a normal metabolism and reduce the likelihood of overeating.<br><br>Overall, my advice is to maintain a well-balanced diet with plenty of vegetables, lean meats and whole grains, while reducing sugar and fat intake. In addition, eating regularly is also beneficial to maintaining good health. The advice given above will hopefully help you maintain a healthier lifestyle.	Rater1: Feedback given is too lengthy.<br><br>Rater2: Feedback given is too lengthy.<br><br>Rater3: None<br><br>Rater4: None
GPT-3.5	Personal Health Information<br><br>Gender: Female<br><br>Age: 37<br><br>Height: 160cm<br><br>Weight: 68.85kg<br><br>Labor intensity: Light labor intensity<br><br>Dietary Information	Based on the breakfast information you provided and your personal health information, I will give you some suggestions.<br><br>First of all, you have a well-balanced breakfast with porridge, eggs, cucumber, watermelon and skimmed milk as healthy food choices. Porridge provides carbohydrates and protein, eggs are rich in protein and fat, cucumber and watermelon contain a lot of water and vitamins, and skimmed milk provides calcium and protein.<br><br>However, depending on your body's condition, I would recommend adding some vegetables or fruits to your breakfast for more fiber and vitamins. You can try adding some salad or fruits like oranges and apples. In addition, depending on your height	Rater1: Feedback given is too lengthy and more like a bot-generated response.<br><br>Rater2: The feedback is too lengthy and misguided. **(Knowledge error)**<br><br>Rater3: Not professional enough and too long. **(Knowledge error)**<br><br>Rater4: None

	Meal type: Breakfast<br><br>Food intake: Half bowl of porridge, 1 egg, 1 cucumber, 2 pieces of watermelon, 250ml of skimmed milk	and weight, you can reduce your porridge and egg intake moderately to control your calorie intake and help you maintain a healthy weight.<br><br>Apart from breakfast, I would also recommend you to choose more vegetables, fruits, whole grains and lean meats in your other meals to keep your diet varied and balanced. It is also recommended that you maintain adequate water intake and eat regularly throughout the day to avoid overeating and irregular meals.<br><br>Finally, adhering to moderate exercise and good lifestyle habits are also key to staying healthy. I wish you the best of luck in maintaining a healthy diet and lifestyle, and please feel free to contact me if you have any questions or need more help.	
ChatGLM3-6B	Personal Health Information<br><br>Gender: Female<br><br>Age: 37<br><br>Height: 160cm<br><br>Weight: 68.85kg<br><br>Labor intensity: Light labor intensity<br><br>Dietary Information<br><br>Meal type: Breakfast<br><br>Food intake: Half bowl of porridge, 1 egg, 1 cucumber, 2 pieces of watermelon, 250ml of skimmed milk	Hello, according to the dietary information you have provided, you have consumed some nutrient-rich foods for breakfast, such as eggs and milk, which are very beneficial for maintaining good health. However, the amount of porridge and watermelon you consumed was high and it is recommended to reduce it appropriately for a more balanced nutritional intake.<br><br>In addition, the information you have provided suggests that your intake of cucumber is high, which may be due to your dietary preference or misuse. It is recommended that you consume a high amount of cucumber while also ensuring that you consume adequate protein, fat and other nutrients to keep your body healthy.<br><br>In terms of formulating your dietary pattern, you are advised to allocate your food intake for each meal according to your height, weight and labor intensity. Generally speaking, breakfast should have a higher calorie intake to replenish the energy lost during night sleep; lunch should have a moderate calorie intake to fulfill the needs of daily work and life; and dinner should have a lower calorie intake to facilitate sleep.<br><br>In order to maintain a healthy diet, it is recommended that you follow the principle of "three lows and one high", i.e. low salt, low sugar, low fat and high fiber. In addition, you should avoid bad eating habits such as overeating and binge eating.<br><br>Finally, if you find yourself unable to stick to a healthy diet, you can seek the help of a professional dietitian to formulate a	Rater1: The dietary feedback is not friendly to understand.<br><br>Rater2: The feedback given is too lengthy.<br><br>Rater3: The readability is terrible.<br><br>Rater4: None

		personalized diet plan in order to achieve the goal of healthy eating.<br><br>I hope these suggestions are helpful, and please feel free to let me know if you have any other questions or need further assistance.	
LLMDF	Personal Health Information<br><br>Gender: Female<br><br>Age: 37<br><br>Height: 160cm<br><br>Weight: 68.85kg<br><br>Labor intensity: Light labor intensity<br><br>Dietary Information<br><br>Meal type: Breakfast<br><br>Food intake: Half bowl of porridge, 1 egg, 1 cucumber, 2 pieces of watermelon, 250ml of skimmed milk	You have eaten a very healthy meal, please keep it up!	Rater1: Too simple and not tailored.<br><br>Rater2: Too simple and not tailored.<br><br>Rater3: None<br><br>Rater4: None
LLMDF-EXP	Personal Health Information<br><br>Gender: Female<br><br>Age: 37<br><br>Height: 160cm<br><br>Weight: 68.85kg<br><br>Labor intensity: Light labor intensity<br><br>Dietary Information<br><br>Meal type: Breakfast<br><br>Food intake: Half bowl of porridge, 1 egg,	Breakfast came together nicely with porridge, eggs, cucumber, watermelon and skimmed milk. The porridge provided energy and satiety. Eggs provide high quality protein and cucumber and watermelon provide a rich source of vitamins and minerals. Skimmed milk provides calcium and vitamin D, which contribute to bone health. It is recommended to continue with this combination and try to choose more whole grains and vegetables and fruits to avoid excessive sugar and fat intake.	Rater1: None<br><br>Rater2: None<br><br>Rater3: None<br><br>Rater4: None

	1 cucumber, 2 pieces of watermelon, 250ml of skimmed milk		
Experts	Personal Health Information<br><br>Gender: Female<br><br>Age: 37<br><br>Height: 160cm<br><br>Weight: 68.85kg<br><br>Labor intensity: Light labor intensity<br><br>Dietary Information<br><br>Meal type: Breakfast<br><br>Food intake: Half bowl of porridge, 1 egg, 1 cucumber, 2 pieces of watermelon, 250ml of skimmed milk	The breakfast combination pattern is basically reasonable, covering staple food, eggs and milk, vegetables and fruits, with rich and diversified food types, and low oil, salt and fat. Skimmed milk is chosen for milk, which is good for weight control, please keep it up. Congee is recommended to choose mixed grain congee, or with boiled corn, steamed sweet potato, etc., to increase satiety.	Rater1: Dietary feedback given is professional.<br><br>Rater2: The advice given is relatively practical and professional.<br><br>Rater3: Concise and professional.<br><br>Rater4: None
GPT-4	Personal Health Information<br><br>Gender: Male<br><br>Age: 47<br><br>Height: 175cm<br><br>Weight: 77.9kg<br><br>Waistline: 86.0cm<br><br>Labor intensity: Light labor intensity<br><br>Dietary Information<br><br>Meal type: Breakfast<br><br>Food intake: 5 wontons with bok choy and tofu, oatmeal, yogurt	Based on the breakfast information and personal health information you provided, I have developed the following suggestions for consideration.<br><br>First, your breakfast choices are fairly balanced and healthy. The tofu ravioli and oatmeal provide protein and carbohydrates, both of which are important sources of energy to maintain morning vigor. Oats as well as tofu are also rich in fiber, which aids in digestion. Meanwhile, yogurt provides probiotics, which are also beneficial for gut health. The only slight caveat is that if your wonton soup base is too oily or overly salty, it is recommended to switch to a clear broth or homemade low-salt, low-sugar version.<br><br>Then, if appropriate, you can add some low-fat meat or fish as appropriate, which will provide more protein to help you maintain your daily stamina needs. You may also want to consider adding a fresh fruit, such as an apple or banana, to increase your vitamin intake.<br><br>In terms of personal health, your body mass index (BMI) and Waistline indicate that you are at risk of being overweight. Therefore, although you don't labor too hard, you need to be careful to control your diet and increase your exercise. Also, as	Rater1: Feedback given is too lengthy and more like a bot-generated response.<br><br>Rater2: The feedback given is too lengthy.<br><br>Rater3: The feedback is comprehensive.<br><br>Rater4: None

		you age, more attention should be paid to the management of blood pressure, lipids and blood sugar in your dietary planning.<br><br>Finally, I encourage you to maintain this healthy diet with some adjustments to fit your individual needs. It is also very important to walk and exercise daily to not only help burn some calories, but also to keep your body energized. I hope these tips have helped you.	
GPT-3.5	Personal Health Information<br><br>Gender: Male<br><br>Age: 47<br><br>Height: 175cm<br><br>Weight: 77.9kg<br><br>Waistline: 86.0cm<br><br>Labor intensity: Light labor intensity<br><br><br>Dietary Information<br><br>Meal type: Breakfast<br><br>Food intake: 5 wontons with bok choy and tofu, oatmeal, yogurt	Based on the information you provided about your breakfast, it looks like you've made some healthier choices such as bok choy and tofu ravioli, oatmeal, and yogurt. These foods provide protein, dietary fiber, and an abundance of vitamins and minerals that are beneficial to your health.<br><br>As a middle-aged man, your body needs more protein to maintain muscle mass and metabolism. I recommend adding protein-rich foods such as eggs, beans or lean meats to your breakfast to help you better meet your nutritional needs.<br><br>Also, depending on your height, weight and intensity of life, your daily calorie needs may range from 2,000-2,500 calories. Make sure your breakfast provides enough energy and keep your food intake moderate to maintain a healthy weight.<br><br>Lastly, it is recommended that you control the portion size of your food during meals and try to use more light, fresh, less oily and less salty ingredients to avoid excessive intake of salt, fats and sugar to maintain your body in a healthy state.<br><br>I hope the above suggestions are helpful to you and wish you good health and happiness!	Rater1: The dietary feedback is not friendly to understand.<br><br>Rater2: Some parts of the content are note friendly to understand.<br><br>Rater3: Some of the content is too theoretical.<br><br>Rater4: None
ChatGLM3-6B	Personal Health Information<br><br>Gender: Male<br><br>Age: 47<br><br>Height: 175cm<br><br>Weight: 77.9kg<br><br>Waistline: 86.0cm<br><br>Labor intensity: Light labor intensity	Hello, Based on the dietary information you have provided, I have noticed that you are consuming a high amount of high protein foods and fats for breakfast and a relatively low intake of vegetables and fiber. This dietary pattern, while providing the body with the nutrients it needs, may increase the risk of heart disease and diabetes in the long run.<br><br>I recommend that you try to maintain a balanced nutritional profile in your future diet by consuming more vegetables and fiber and choosing foods that are low in fat, salt and sugar. For breakfast, you may want to consider pairing bok choy and tofu wontons with oatmeal and some fresh fruit, which provides enough protein and carbohydrates while still being rich in fiber	Rater1: The feedback given is too lengthy.<br><br>Rater2: The feedback given is too long and is more like a robot-generated response, with misunderstandings and inconsistencies. **(Misunderstanding and inconsistency)**<br><br>Rater3: The feedback given is too lengthy.<br><br>Rater4: None

	Dietary Information<br><br>Meal type: Breakfast<br><br>Food intake: 5 wontons with bok choy and tofu, oatmeal, yogurt	and vitamins.<br><br>In addition, I note that you have a light labor-intensive body, so it would also be appropriate to add some energy intake to your diet to keep you healthy. Specifically, you can add some nuts, seeds, whole grains, and other foods rich in healthy fats and proteins.<br><br>Lastly, I recommend that you have regular medical checkups so that you can keep up with your health and adjust your diet based on the results. I hope these suggestions are helpful and I wish you good health!	
LLMDF	Personal Health Information<br><br>Gender: Male<br><br>Age: 47<br><br>Height: 175cm<br><br>Weight: 77.9kg<br><br>Waistline: 86.0cm<br><br>Labor intensity: Light labor intensity<br><br>Dietary Information<br><br>Meal type: Breakfast<br><br>Food intake: 5 wontons with bok choy and tofu, oatmeal, yogurt	Breakfast is still good, but it is recommended to pair it with a serving of eggs, such as hard-boiled, fried, or steamed eggs.	Rater1: Too simple and not tailored.<br><br>Rater2: Too simple.<br><br>Rater3: None<br><br>Rater4: None
LLMDF-EXP	Personal Health Information<br><br>Gender: Male<br><br>Age: 47<br><br>Height: 175cm<br><br>Weight: 77.9kg<br><br>Waistline: 86.0cm	Breakfast is a good combination, green vegetables and tofu wontons are a good choice, oatmeal and yogurt are also healthy. However, your weight and waistline are both slightly high. It is recommended that you control your food intake by moderately reducing the intake of staple foods and fats, increasing the intake of vegetables and fruits, and maintaining a balanced diet.	Rater1: None<br><br>Rater2: The advice given is relatively practical and professional.<br><br>Rater3: Clear and very hands-on.<br><br>Rater4: None

	Labor intensity: Light labor intensity<br><br>Dietary Information<br><br>Meal type: Breakfast<br><br>Food intake: 5 wontons with bok choy and tofu, oatmeal, yogurt		
Experts	Personal Health Information<br><br>Gender: Male<br><br>Age: 47<br><br>Height: 175cm<br><br>Weight: 77.9kg<br><br>Waistline: 86.0cm<br><br>Labor intensity: Light labor intensity<br><br>Dietary Information<br><br>Meal type: Breakfast<br><br>Food intake: 5 wontons with bok choy and tofu, oatmeal, yogurt	Breakfast covers staples, dairy, vegetables and soy products. The choice of oatmeal for the main course has achieved a good mix of roughage and fines, so please keep it up! Breakfast is recommended to add an egg to make the food variety richer and more diversified, while increasing high-quality protein. Yogurt is recommended to choose low-sugar hu or sugar-free yogurt, which is good for weight control.	Rater1: Dietary feedback given is professional.<br><br>Rater2: The advice given is relatively practical and professional.<br><br>Rater3: Clear and very hands-on.<br><br>Rater4: None
GPT-4	Personal Health Information<br><br>Gender: Male<br><br>Age: 52<br><br>Height: 166cm<br><br>Weight: 73.9kg<br><br>Labor intensity: Light labor intensity<br><br>Dietary Information	From the dietary information you have provided, it appears that the breakfast is comprehensive and includes protein (eggs), complex carbohydrates (corn and sweet potatoes), and calcium (milk). This is a great breakfast combination and is good for your body. However, the portion size aspect may need to be adjusted appropriately to ensure that the breakfast provides enough energy.<br><br>Considering that you are a 52-year-old male, 166cm tall, 73.9kg, with a light workload, your basal metabolic rate and energy consumption for physical activity are relatively low. We need to keep the dietary calories in the right range to avoid excessive energy intake that leads to weight gain and not too low that leads to decreased physical function.	Rater1: Feedback given is too lengthy and more like a bot-generated response.<br><br>Rater2: The advice given is relatively practical and professional, and it is suggested that it could be more streamlined.<br><br>Rater3: None<br><br>Rater4: None

	Meal type: Breakfast<br><br>Food intake: One egg, one corn, one small sweet potato, one cup of milk	I would suggest that you can try to balance each meal and keep an eye on your calorie intake at all times, try to focus on vegetables with the right amount of meat and grains. In addition, fruit is a great snack option. You can also consider increasing your exercise to improve your body's metabolism.<br><br>The most important principles of diet are still balance and moderation. Physical health is a long-term process that requires us to work on it day after day, year after year. I hope you will understand and follow this dietary pattern and gradually develop healthy eating habits.	
GPT-3.5	Personal Health Information<br><br>Gender: Male<br><br>Age: 52<br><br>Height: 166cm<br><br>Weight: 73.9kg<br><br>Labor intensity: Light labor intensity<br><br><br>Dietary Information<br><br>Meal type: Breakfast<br><br>Food intake: One egg, one corn, one small sweet potato, one cup of milk	Based on the breakfast information and personal health information you provided, I can give the following dietary feedback:<br><br>1. analyze the attributes of the food consumed by the client: you consumed some staple food groups and proteins in your breakfast, but I noticed that you lacked vegetables and fruits in your breakfast, which may have led to a less balanced dietary structure.<br><br>2. Provide healthy food choices: It is recommended that you increase the intake of vegetables and fruits in your breakfast, which will provide more vitamins and fiber to help you stay healthy.<br><br>3. help clients to develop dietary patterns: you can try adding vegetable salad or fruit salad to breakfast, or choosing a balanced and nutritious breakfast, such as whole wheat bread with tomatoes and eggs and a fruit salad.<br><br>4. Encourage clients to maintain healthy eating habits: It is very important to maintain a balanced diet. You can try to eat a variety of foods at each meal to maintain a balanced diet and avoid partiality or excessive intake of high-calorie foods.<br><br>5. Advise your clients to modify their dietary habits: Apart from breakfast, you need to pay attention to the choice of lunch, dinner and snacks to ensure that your dietary intake is healthy and balanced throughout the day.<br><br>I hope the above suggestions are helpful to you. If you have any questions or need further guidance, please feel free to contact me. Best of luck in maintaining a healthy lifestyle!	Rater1: The dietary feedback is not friendly to understand.<br><br>Rater2: Feedback given is too lengthy and more like a bot-generated response.<br><br>Rater3: The feedback given is too lengthy.<br><br>Rater4: None

ChatGLM3-6B	Personal Health Information<br><br>Gender: Male<br><br>Age: 52<br><br>Height: 166cm<br><br>Weight: 73.9kg<br><br>Labor intensity: Light labor intensity<br><br><br>Dietary Information<br><br>Meal type: Breakfast<br><br>Food intake: One egg, one corn, one small sweet potato, one cup of milk	Based on the dietary information you provided, I see that your breakfast intake is relatively well-balanced, containing protein, carbohydrates, and fat, as well as a moderate amount of fiber. This is a good start. However, I note that your breakfast intake of eggs and milk may be high, which may increase your daily calorie intake. It is recommended that you take care to control your calorie intake while maintaining a balanced nutritional profile.<br><br>In terms of food choices, you are advised to consume more vegetables and fruits to increase your intake of vitamins and minerals. For example, you can replace eggs and milk with a serving of fruit and a cup of low-fat yogurt. In addition, corn and small sweet potatoes are good sources of nutrients, but they are high in carbohydrates and it is recommended that you consume them in moderation during meals.<br><br>In terms of eating patterns, it is recommended that you try to maintain a regular pattern of three meals per day and avoid overeating or excessive hunger. This helps keep your blood sugar levels stable and helps control weight gain.<br><br>Lastly, I would recommend that you take care to modify your diet by, for example, cutting down on fatty and sugary foods and increasing your intake of vegetables and fruits. This will help improve your health and reduce the risk of disease.<br><br>This is my feedback on your dietary information and I hope it is helpful. If you have any further questions, please feel free to ask me.	Rater1: The feedback given is too lengthy.<br><br>Rater2: Misunderstanding of the content of dietary intake. **(Misunderstanding)**<br><br>Rater3: None<br><br>Rater4: None
LLMDF	Personal Health Information<br><br>Gender: Male<br><br>Age: 52<br><br>Height: 166cm<br><br>Weight: 73.9kg<br><br>Labor intensity: Light labor intensity<br><br><br>Dietary Information	Breakfast was overall good, keep it up.	Rater1: Too simple and not tailored.<br><br>Rater2: Too simple and not tailored.<br><br>Rater3: None<br><br>Rater4: None

	Meal type: Breakfast<br><br>Food intake: One egg, one corn, one small sweet potato, one cup of milk		
LLMDF-EXP	Personal Health Information<br><br>Gender: Male<br><br>Age: 52<br><br>Height: 166cm<br><br>Weight: 73.9kg<br><br>Labor intensity: Light labor intensity<br><br>Dietary Information<br><br>Meal type: Breakfast<br><br>Food intake: One egg, one corn, one small sweet potato, one cup of milk	The breakfast options are still good with a combination of proteins, carbohydrates and fats. However, it is recommended that you add some vegetables or fruits to get more fiber and vitamins. For example, you can add some tomatoes or cucumbers to your breakfast, which are rich in vitamin C and fiber, which are great for your health. Corn and small sweet potatoes provide dietary fiber and complex carbohydrates that help stabilize blood sugar and satiety. Milk is a good source of protein and calcium to maintain healthy bones. Finally, it is recommended that you continue to maintain healthy breakfast choices and be mindful of controlling your total calorie and fat intake.	Rater1: None<br><br>Rater2: The advice given is relatively practical and professional.<br><br>Rater3: None<br><br>Rater4: None
Experts	Personal Health Information<br><br>Gender: Male<br><br>Age: 52<br><br>Height: 166cm<br><br>Weight: 73.9kg<br><br>Labor intensity: Light labor intensity<br><br>Dietary Information<br><br>Meal type: Breakfast<br><br>Food intake: One egg, one corn, one small sweet potato, one cup of milk	Breakfast covers staple food and egg and milk, and the choice of staple food is corn, sweet potato and other high quality carbohydrate staple food rich in dietary fiber, please keep it up! Easy-to-digest staples such as steamed buns and porridge can be added appropriately to avoid indigestion. Milk is recommended to choose skimmed or low-fat milk, which is good for weight control. It is recommended to add another low-sugar fruit or a serving of vegetables for breakfast.	Rater1: Dietary feedback given is professional.<br><br>Rater2: The advice given is relatively practical and professional.<br><br>Rater3: None<br><br>Rater4: None

GPT-4	Personal Health Information<br><br>Gender: Male<br><br>Age: 22<br><br>Height: 169cm<br><br>Weight: 72.0kg<br><br>Labor intensity: Light labor intensity<br><br><br>Dietary Information<br><br>Meal type: Lunch<br><br>Food intake: Stir-fried beef 500g; Stir-fried celery 150g; Braised eggplant 400g; Rice (steamed) 200g.	From the dietary information you have provided, it seems that the types of food you consume in lunch, although including vegetables and meat, are nutritionally biased towards protein and fat, with relatively few grains and cereals. The way beef and eggplants are cooked has a greater impact on our health, especially if they are cooked with a lot of oil and salt, which may cause high blood cholesterol and other health problems.<br><br>Regarding healthier choices, you may want to consider making changes in the way your food is cooked, such as trying steaming, boiling, or roasting to reduce the amount of fat you consume. Also, include a wider variety of vegetables, such as broccoli and red peppers, which will not only provide you with the vitamins and minerals you need, but will also help you feel full.<br><br>In terms of dietary patterns, it is recommended that you increase your intake of grains and cereals, such as replacing some of your rice with whole grain bread or oatmeal, which hangs better and can provide richer nutrients. At the same time, try to keep every meal with meat and vegetables, balanced food intake can help your body to get the required nutrients.<br><br>Since everyone's physical condition and lifestyle are different, a good diet should be adjusted according to your actual situation. For your current situation, it is recommended that you try to eat your dinner two hours before every day and avoid eating immediately before bedtime, which is not only good for digestion, but also prevents excessive energy intake at night.<br><br>Overall, a healthy diet does not only mean eating healthy, but more importantly, establishing rational and healthy food choices and dining habits. I hope these tips will be helpful.	Rater1: The feedback given is too lengthy.<br><br>Rater2: The feedback given is too lengthy.<br><br>Rater3: None<br><br>Rater4: None
GPT-3.5	Personal Health Information<br><br>Gender: Male<br><br>Age: 22<br><br>Height: 169cm<br><br>Weight: 72.0kg<br><br>Labor intensity: Light labor intensity	Based on the dietary information you have provided, I can see that your lunch is more on the heavy side and high in calories. In order to maintain a healthy diet, I suggest you can take the following measures:<br><br>1. consume more vegetables and fruits: increase the intake of dietary fiber, which helps to promote intestinal peristalsis, slow down the rate of blood glucose increase and provide a sense of satiety.<br><br>2. moderate control of meat intake: you can reduce the intake of meat and fat, choose lean meat and less oil cooking methods to reduce calorie intake.	Rater1: The dietary feedback is not friendly to understand.<br><br>Rater2: None<br><br>Rater3: None<br><br>Rater4: None

	Dietary Information<br><br>Meal type: Lunch<br><br>Food intake: Stir-fried beef 500g; Stir-fried celery 150g; Braised eggplant 400g; Rice (steamed) 200g.	3. Reasonable matching of staple foods: rice can be reduced in moderation to increase the intake of grains, potatoes and other staple foods, in order to increase the intake of dietary fiber and multivitamins.<br><br>4. Focus on dietary diversity: Try to avoid the intake of single food, a diversified diet can provide a wider variety of nutrients, which can help maintain health.<br><br>Finally, it is recommended that you exercise in moderation and maintain good habits and a regular diet, which can better help you achieve a healthy lifestyle and ideal weight. Please feel free to contact me if you have any questions or need further guidance. I wish you good health!	
ChatGLM3-6B	Personal Health Information<br><br>Gender: Male<br><br>Age: 22<br><br>Height: 169cm<br><br>Weight: 72.0kg<br><br>Labor intensity: Light labor intensity<br><br>Dietary Information<br><br>Meal type: Lunch<br><br>Food intake: Stir-fried beef 500g; Stir-fried celery 150g; Braised eggplant 400g; Rice (steamed) 200g.	Hello, based on the dietary information you have provided, I have found that your calorie intake is high, while the ratio of protein, fat and carbohydrate intake is more balanced. For a 22 year old male light laborer, such a diet, while not directly leading to health problems, may have some potential risks.<br><br>First, your protein intake is more adequate, which is helpful for maintaining muscle and staying healthy. However, there may be a risk of obesity due to your higher calorie intake. In addition, the higher intake of rice may lead to fluctuations in blood sugar, which may be a concern for those who are controlling diabetes or high blood sugar.<br><br>To address this situation, I recommend that you control your calorie intake appropriately while ensuring balanced nutrition. You can try to reduce the intake of rice and increase the intake of vegetables and fruits to balance the diet structure. In addition, you can appropriately increase the amount of exercise to help burn excess calories and prevent obesity.<br><br>When formulating a diet plan, it should be based on your own physical condition and needs. If you have any questions or need further advice, please feel free to consult me.	Rater1: Feedback given is too lengthy and more like a bot-generated response.<br><br>Rater2: Some of the content is wrong. **(Knowledge error)**<br><br>Rater3: There is a professional error, 200 grams of steamed rice is not much, rather 500 grams of beef is too much. **(Knowledge error)**<br><br>Rater4: None
LLMDF	Personal Health Information<br><br>Gender: Male<br><br>Age: 22	Lunch choices are still very good, but the amount of meat is on the high side and the amount of rice is slightly too much, so it is recommended that the rice can be eaten in smaller portions, and the percentage of vegetables is recommended to be increased a bit more.	Rater1: None<br><br>Rater2: The feedback is not comprehensive.

	Height: 169cm<br><br>Weight: 72.0kg<br><br>Labor intensity: Light labor intensity<br><br>Dietary Information<br><br>Meal type: Lunch<br><br>Food intake: Stir-fried beef 500g; Stir-fried celery 150g; Braised eggplant 400g; Rice (steamed) 200g.		Rater3: There was a professional error, two veggies and one meat, with a good amount of veggies, was a matter of cooking style. **(Knowledge error)**<br><br>Rater4: None
LLMDF-EXP	Personal Health Information<br><br>Gender: Male<br><br>Age: 22<br><br>Height: 169cm<br><br>Weight: 72.0kg<br><br>Labor intensity: Light labor intensity<br><br>Dietary Information<br><br>Meal type: Lunch<br><br>Food intake: Stir-fried beef 500g; Stir-fried celery 150g; Braised eggplant 400g; Rice (steamed) 200g.	The combination of meat and vegetables in lunch is fine. It is recommended to reduce your rice intake slightly and increase the intake of vegetables, such as increasing the amount of fried celery. In addition, pay attention to control the intake of fat, you can choose less oil frying way, reduce the intake of fat.	Rater1: Dietary feedback is professional.<br><br>Rater2: The advice given is relatively practical and professional.<br><br>Rater3: None<br><br>Rater4: None
Experts	Personal Health Information<br><br>Gender: Male<br><br>Age: 22<br><br>Height: 169cm<br><br>Weight: 72.0kg<br><br>Labor intensity: Light labor intensity	Lunch in general, the food variety is still relatively rich, the current weight is overweight, need to further control weight, to choose light and less oil dishes, stewed eggplant and these high oil dishes are not recommended to choose.	Rater1: None<br><br>Rater2: None<br><br>Rater3: The feedback could be more comprehensive.<br><br>Rater4: None

	Dietary Information<br><br>Meal type: Lunch<br><br>Food intake: Stir-fried beef 500g; Stir-fried celery 150g; Braised eggplant 400g; Rice (steamed) 200g.		
GPT-4	Personal Health Information<br><br>Gender: Female<br><br>Age: 20<br><br>Height: 155cm<br><br>Weight: 66.0kg<br><br>Labor intensity: Light labor intensity<br><br>Dietary Information<br><br>Meal type: Lunch<br><br>Food intake: Stir-fried black fungus with baby bok choy 110g; White rice 100g; Steamed fish 110g; Tomato and egg soup 200g.	First of all, your lunch food choices are fantastic. Stir-fried black fungus with baby bok choy, steamed fish and tomato and egg soup are all excellent healthy foods, rich in fiber, protein and trace elements, and at the same time low in fat and sugar, which is very helpful for maintaining good health. However, I notice that you are consuming quite a lot of rice, so I would suggest that you reduce your intake of white rice moderately, or replace it with healthier grains and cereals, which will increase your fiber intake and help you feel full.<br><br>Next, let's talk about your dietary pattern. Based on your "light labor intensity" physical load and your age and weight in your 20s, you should have enough calories to support your daily life and activities. We can increase your protein source to increase satiety and reduce your intake of staple foods such as white rice or replace them with healthier complex carbohydrates such as oats and barley.<br><br>Finally, I hope you'll maintain healthy eating habits over time, such as keeping breakfast plentiful, as well as eating plenty of fruits and vegetables and high-quality protein foods. And don't forget that diet is just one part of a healthy lifestyle; remember to exercise regularly and maintain a good mindset.<br><br>Overall, your current diet is good overall, with just a few small tweaks needed to make your diet both healthy and varied.	Rater1: The feedback given is too lengthy.<br><br>Rater2: The feedback given is too lengthy and has and repetitive content.<br><br>Rater3: None<br><br>Rater4: None
GPT-3.5	Personal Health Information<br><br>Gender: Female<br><br>Age: 20<br><br>Height: 155cm<br><br>Weight: 66.0kg	Based on the dietary information you have provided, I notice that your lunch are relatively well-balanced, containing vegetables, fish and soups, which is very good for maintaining good health. However, I would also like to remind you of the following points:<br><br>1. Low vegetable intake: Although baby bok choy and black fungus are very healthy ingredients, a 110g serving is relatively small. It is recommended that you increase your vegetable intake by considering a wider variety of vegetables or increasing the portion size.	Rater1: None<br><br>Rater2: The advice given is relatively practical and professional, and it is suggested that it could be more streamlined.<br><br>Rater3: None<br><br>Rater4: None

	Labor intensity: Light labor intensity<br><br>Dietary Information<br><br>Meal type: Lunch<br><br>Food intake: Stir-fried black fungus with baby bok choy 110g; White rice 100g; Steamed fish 110g; Tomato and egg soup 200g.	2. Excessive intake of white rice: 100 grams of white rice has a high calorie content. For those who want to control their weight, it is recommended to reduce the amount of rice intake or choose healthier staple foods such as brown rice as a substitute.<br><br>3. It is recommended to increase protein intake in moderation: Steamed fish and tomato and egg soup provide a certain amount of protein, but for your physical condition, you may need more protein to meet your body's needs, so you may consider increasing sources of protein such as chicken, eggs and soy products.<br><br>On the whole, your diet is still relatively healthy, but there are some details that can be adjusted to better meet your body's needs. I hope you can adjust your diet appropriately according to my suggestions, and I also recommend maintaining a moderate amount of exercise, which can help you maintain better health. Please feel free to contact me if you have any questions or need more advice. I wish you good health!	
ChatGLM3-6B	Personal Health Information<br><br>Gender: Female<br><br>Age: 20<br><br>Height: 155cm<br><br>Weight: 66.0kg<br><br>Labor intensity: Light labor intensity<br><br>Dietary Information<br><br>Meal type: Lunch<br><br>Food intake: Stir-fried black fungus with baby bok choy 110g; White rice 100g; Steamed fish 110g; Tomato and egg soup 200g.	Hello, based on the dietary information you have provided, your intake food attributes are primarily based on rice and vegetables, which is a very healthy eating pattern that helps to keep you feeling full and maintain stable blood sugar levels. However, your protein intake is relatively low and may need to be supplemented by increasing the amount of meat, legumes or other protein sources.<br><br>In terms of food choices, you are advised to choose more fresh and natural ingredients and avoid excessive processed foods and sugary drinks. For vegetables, you can increase your intake of baby bok choy and black fungus, which are both rich in fiber and micronutrients that help maintain intestinal health and lower cholesterol. For meat, steamed fish is a better choice, which is low in fat and rich in nutrients. Meanwhile, tomato and egg soup also provides rich nutrients, but you are advised to reduce the intake of oil and salt.<br><br>In terms of dietary patterns, you are advised to keep three regular meals, with a rich breakfast, a full midday meal and a light dinner. Meanwhile, increasing the intake of fruits and nuts appropriately will help replenish vitamins and minerals.<br><br>Finally, it is recommended that you maintain good eating habits,	Rater1: Feedback given is too lengthy and more like a bot-generated response.<br><br>Rater2: The feedback given is too lengthy.<br><br>Rater3: The feedback given is too lengthy.<br><br>Rater4: None

		avoid overeating, avoid late-night snacking, and avoid eating too much high-calorie, high-fat, and high-sugar food. Also, maintaining good exercise habits will help you maintain a healthy weight and physical health.<br><br>I hope these suggestions are helpful and please feel free to let me know if you have any other questions or need further dietary guidance.	
LLMDF	Personal Health Information<br><br>Gender: Female<br><br>Age: 20<br><br>Height: 155cm<br><br>Weight: 66.0kg<br><br>Labor intensity: Light labor intensity<br><br><br>Dietary Information<br><br>Meal type: Lunch<br><br>Food intake: Stir-fried black fungus with baby bok choy 110g; White rice 100g; Steamed fish 110g; Tomato and egg soup 200g.	The choice of ingredients is still very good, it is the amount of vegetables is slightly less, it is recommended to increase the amount of vegetables more in the later dish selection.	Rater1: Too simple.<br><br>Rater2: Too simple.<br><br>Rater3: Concise.<br><br>Rater4: None
LLMDF-EXP	Personal Health Information<br><br>Gender: Female<br><br>Age: 20<br><br>Height: 155cm<br><br>Weight: 66.0kg<br><br>Labor intensity: Light labor intensity<br><br><br>Dietary Information<br><br>Meal type: Lunch	This is a great meal with a well-balanced mix of vegetables, protein, and entrees. The baby bok choy and black fungus are both low-calorie and high-fiber ingredients that are helpful for weight control and digestive health. The steamed fish provided a good source of protein, while the tomato and egg soup provided vitamins and minerals. It is recommended to continue with this balanced dietary pattern, moderately controlling the intake of staple foods and taking care to cook in a way that is as light and less oily and salty as possible to help maintain a healthy weight.	Rater1: Dietary feedback is professional.<br><br>Rater2: None<br><br>Rater3: None<br><br>Rater4: None

	Food intake: Stir-fried black fungus with baby bok choy 110g; White rice 100g; Steamed fish 110g; Tomato and egg soup 200g.		
Experts	Personal Health Information<br><br>Gender: Female<br><br>Age: 20<br><br>Height: 155cm<br><br>Weight: 66.0kg<br><br>Labor intensity: Light labor intensity<br><br>Dietary Information<br><br>Meal type: Lunch<br><br>Food intake: Stir-fried black fungus with baby bok choy 110g; White rice 100g; Steamed fish 110g; Tomato and egg soup 200g.	Lunch is excellent, following the formula of carb + protein + veggies, and choosing dishes that are light, with low-fat aquatic choices for meat, so please keep it up!	Rater1: Feedback could be more comprehensive.<br><br>Rater2: None<br><br>Rater3: None<br><br>Rater4: None
GPT-4	Personal Health Information<br><br>Gender: Female<br><br>Age: 31<br><br>Height: 156cm<br><br>Weight: 57.0kg<br><br>Labor intensity: Light labor intensity<br><br>Dietary Information<br><br>Meal type: Lunch<br><br>Food intake: Rice + eggplant + shrimp and green beans + tomato scrambled eggs	Based on the dietary information you have provided, you have a fairly rich allotment of cereals, a wide variety of vegetables, and high-quality protein sources in your lunch. Rice supplies most of the calories your body needs for daily exercise, and vegetables such as eggplant cover a rich source of vitamins and minerals, as well as antioxidants, which are good for maintaining body function and anti-aging. Shrimp and eggs are both sources of high-quality protein, which also contributes to bone and muscle health.<br><br>However, to better control your calorie intake and maintain good body condition, you may consider controlling your rice intake at each meal or try other grains such as brown rice, whole grain breads, etc. And, try to control the use of salt and fat when cooking vegetables and eggs.<br><br>Colorful vegetables and fruits can add to the appeal of your diet, such as red carrots or green peppers, and a variety of colors means a variety of nutrients. Also, try a variety of protein	Rater1: The feedback given is too lengthy.<br><br>Rater2: Content is too long, response could be more concise.<br><br>Rater3: The feedback is too long.<br><br>Rater4: None

		sources, such as tofu, chicken or fish.<br><br>Considering your age and weight, your activity intensity is low and you should be careful to control your calorie intake to avoid being overweight, while increasing your physical activity to help with calorie burning and to help maintain a healthy heart and blood vessels.<br><br>I suggest you try recording your diet on a regular basis to get a more accurate picture of what you are eating and to continue to maintain good eating habits to make a healthy lifestyle a daily routine.	
GPT-3.5	Personal Health Information<br><br>Gender: Female<br><br>Age: 31<br><br>Height: 156cm<br><br>Weight: 57.0kg<br><br>Labor intensity: Light labor intensity<br><br><br>Dietary Information<br><br>Meal type: Lunch<br><br>Food intake: Rice + eggplant + shrimp and green beans + tomato scrambled eggs	Based on the dietary information you provided, I noticed that your lunch pairing included rice, eggplant, shrimp and green beans, and scrambled eggs with tomatoes. This combination meal has a variety of food groups such as staples, vegetables, and proteins, and it is very important to have a varied diet.<br><br>It is recommended that you add more types of vegetables, such as spinach, broccoli, and carrots, to your lunch to ensure that you are getting enough fiber and vitamins. You may also want to add some beans, nuts or whole grains in moderation to increase your intake of dietary fiber and quality carbohydrates.<br><br>Based on your personal health information, I recommend you to have a balanced intake of all kinds of nutrients, control the way you cook your food, and avoid greasy and high-salt foods. In addition, you can increase your exercise according to your physical needs in order to maintain a healthy weight and fitness level.<br><br>Last but not least, diet is a habit. It is recommended that you develop good eating habits in your daily life, do not over-eat or overeat, and keep your diet stable and varied, which will help you maintain a healthy lifestyle. I hope my advice is helpful and I wish you good health and happiness!	Rater1: Feedback given is too lengthy and more like a bot-generated response.<br><br>Rater2: The advice given is relatively practical and professional and could be expressed in a more concise manner.<br><br>Rater3: Not professional enough to add beans when you've clearly eaten green beans. **(Inconsistency)**<br><br>Rater4: None
ChatGLM3-6B	Personal Health Information<br><br>Gender: Female<br><br>Age: 31<br><br>Height: 156cm<br><br>Weight: 57.0kg	Hello, according to the dietary information you provided, your intake of food attributes is mainly rice and meat, with a low intake of vegetables, and the scrambled eggs with tomato contains a high level of fat. This dietary pattern may have a nutritional imbalance. It is recommended that you focus on vegetable and fruit intake in your future diets to ensure that your body receives sufficient fiber and vitamins.	Rater1: The dietary feedback is not friendly to understand.<br><br>Rater2: The feedback given is too long and is more like a robot-generated response, with misunderstandings and inconsistencies. **(Misunderstanding and inconsistency)**

	Labor intensity: Light labor intensity<br><br>Dietary Information<br><br>Meal type: Lunch<br><br>Food intake: Rice + eggplant + shrimp and green beans + tomato scrambled eggs	In addition, according to your personal health information, you are a female, 31 years old, 156cm tall, weighing 57.0kg, and are classified as light labor intensive. While maintaining a normal diet, it is recommended that you increase your protein and fat intake to meet your body's needs, depending on your physical condition and labor demands.<br><br>For specific food choices, you can consider increasing the variety of vegetables, such as green vegetables, beans, roots and tubers, which are rich in vitamins, minerals and dietary fiber, and are good for the body. At the same time, you can appropriately reduce the intake of greasy foods, such as fried foods and deep-fried foods.<br><br>When developing a dietary pattern, you can consider adopting the "meal + fruit + two steps" pattern, i.e., eat a piece of fruit after each meal and take two steps between each meal to help digestion and prevent obesity.<br><br>Lastly, it is recommended that you maintain healthy eating habits, avoid over-eating, overeating and other bad habits, and maintain good living habits to achieve the goal of good health. If you have any questions, please feel free to ask me.	Rater3: None<br><br>Rater4: None
LLMDF	Personal Health Information<br><br>Gender: Female<br><br>Age: 31<br><br>Height: 156cm<br><br>Weight: 57.0kg<br><br>Labor intensity: Light labor intensity<br><br>Dietary Information<br><br>Meal type: Lunch<br><br>Food intake: Rice + eggplant + shrimp and green beans + tomato scrambled eggs	Lunch is a combination of meat and vegetables, the amount of vegetables is slightly on the low side, you can increase the amount of vegetables at a later stage.	Rater1: Too simple and not tailored.<br><br>Rater2: The feedback could be more comprehensive.<br><br>Rater3: None<br><br>Rater4: None
LLMDF-EXP	Personal Health Information<br><br>Gender: Female	Your lunch is more reasonably balanced with staple food, vegetables and protein intake. However, it is recommended to control the intake of rice and increase the portion of vegetables in	Rater1: None

	Age: 31<br><br>Height: 156cm<br><br>Weight: 57.0kg<br><br>Labor intensity: Light labor intensity<br><br><br>Dietary Information<br><br>Meal type: Lunch<br><br>Food intake: Rice + eggplant + shrimp and green beans + tomato scrambled eggs	moderation. You can eat more green leafy vegetables, such as spinach and bok choy, to increase the intake of dietary fiber. In addition, shrimp and green beans and tomato scrambled eggs are healthier sources of protein, but also pay attention to the cooking method, try to minimize deep-frying or over-fried, choose steamed, boiled or baked, in order to reduce the intake of fats and oils. Continuing to maintain this balanced diet and paying attention to the types of food and the way they are cooked will help maintain healthy eating habits.	Rater2: The advice given is relatively practical and professional.<br><br>Rater3: None<br><br>Rater4: None
Experts	Personal Health Information<br><br>Gender: Female<br><br>Age: 31<br><br>Height: 156cm<br><br>Weight: 57.0kg<br><br>Labor intensity: Light labor intensity<br><br><br>Dietary Information<br><br>Meal type: Lunch<br><br>Food intake: Rice + eggplant + shrimp and green beans + tomato scrambled eggs	There were six food groups for lunch, covering staples, vegetables, and quality protein sources. The overall variety is okay. However, the amount of oil in this meal is easily exceeded and needs attention. Eggplant and scrambled egg in the dish itself absorbs oil easily, so it is recommended to reduce the choices or opt for steaming.	Rater1: Dietary feedback is professional.<br><br>Rater2: None<br><br>Rater3: None<br><br>Rater4: None
GPT-4	Personal Health Information<br><br>Gender: Female<br><br>Age: 33<br><br>Height: 167cm<br><br>Weight: 87.0kg	From your dietary information, I would first focus on your lunch food choices. Curry Pork Chop Rice is a very tasty but also higher calorie meal which is high in fat and carbohydrates. However, a balanced and nutritious diet is key. Although your labor intensity is moderate, consuming high-calorie food for a long period of time may lead to weight gain. In addition to this, although coffee has a refreshing effect, excessive intake may also have health implications.<br><br>For you, it is recommended to replace a portion of your curry	Rater1: The dietary feedback is not friendly to understand.<br><br>Rater2: Content is too long, response could be more concise.<br><br>Rater3: None<br><br>Rater4: None

	Labor intensity: Medium labor intensity<br><br>Dietary Information<br><br>Meal type: Lunch<br><br>Food intake: Curry pork chop rice, coffee	pork chop rice with more vegetables and lean meat-based foods. Fiber-rich vegetables help you maintain a sense of fullness while reaping more micronutrients and vitamins, while lean meat provides the quality protein your body needs. If you need a pick-me-up, it's recommended to cut down on your coffee intake and drink more water or opt for green tea.<br><br>In addition, a meal with meat is not necessarily a healthy choice, and likewise, a meal without meat is unbalanced. To keep your diet varied, try a meal with fish and tofu for dinner.<br><br>In terms of dietary patterns, I suggest you try eating three meals a day at regular intervals to ensure that each meal is adequately nourished while maintaining good eating habits. Be sure not to skip meals, let alone starve or satiate, which not only tends to affect your health, but may also lead to weight fluctuations.<br><br>In the diet is a constant battle, you need to adhere to scientific and reasonable eating habits. The above suggestions are based on your current diet and health information, if there is any discomfort, or you have other dietary preferences, please feel free to provide the information again and I will provide you with more suitable dietary suggestions. I hope this helps you to carry on with a healthier lifestyle.	
GPT-3.5	Personal Health Information<br><br>Gender: Female<br><br>Age: 33<br><br>Height: 167cm<br><br>Weight: 87.0kg<br><br>Labor intensity: Medium labor intensity<br><br>Dietary Information<br><br>Meal type: Lunch<br><br>Food intake: Curry pork chop rice, coffee	Based on the dietary information you provided, I noticed that your lunch choices were mainly curry pork chop rice and coffee. Curry pork chop rice may contain a lot of fat and calories, while coffee, while providing some refreshment, may also lead to caffeine-induced sleep problems. As a dietitian, I recommend that you choose more vegetables and protein in your lunch, such as a vegetable salad with grilled chicken breast or salmon. This will increase satiety and provide more nutrients.<br><br>Also, based on your personal health information, you are heavier, so I recommend that you increase your exercise while controlling your calories in order to lose weight. You can choose moderate aerobic exercise such as jogging, swimming or yoga to help burn excess fat. It is also very important to maintain a regular schedule and get enough sleep to ensure good health and weight loss.<br><br>Overall, a healthy lifestyle is not only about dietary control, but also includes exercise and a good routine. I hope you can use my advice and guidance to adjust your eating habits and adopt a healthy lifestyle to stay healthy and energized! If you have any	Rater1: The feedback given is too lengthy.<br><br>Rater2: None<br><br>Rater3: None<br><br>Rater4: None

		questions or need further help, please feel free to contact me. I wish you good health and a happy life!	
ChatGLM3-6B	PPersonal Health Information<br><br>Gender: Female<br><br>Age: 33<br><br>Height: 167cm<br><br>Weight: 87.0kg<br><br>Labor intensity: Medium labor intensity<br><br><br>Dietary Information<br><br>Meal type: Lunch<br><br>Food intake: Curry pork chop rice, coffee	Dear Customer, according to the dietary information provided by you, the main food components of your intake of curry pork chop rice and coffee are based on meat and carbohydrates, which may lead to higher calorie intake and fat accumulation. According to your personal health information, your age, weight and labor intensity are at an average level. Therefore, you are advised to focus on the following points while maintaining a balanced diet:<br><br>1. Increase your intake of vegetables and fruits. Vegetables and fruits are rich in fiber and various vitamins that help maintain good health. You can choose to eat more green vegetables such as spinach and celery, as well as rich fruits such as apples and bananas.<br><br>2. Control the intake of carbohydrates. Although carbohydrates are an important source of energy for your body, excessive intake of carbohydrates can lead to weight gain. It is recommended that you moderate your intake of staple foods such as rice and noodles, and try to increase your intake of coarse grains such as brown rice and whole wheat bread.<br><br>3. Reduce the intake of greasy food. Curry Pork Chop Rice is high in fat, so you are advised to moderate your intake and try to choose healthier cooking methods, such as steaming and boiling.<br><br>4. Maintain a varied diet. Variety of food can help provide many nutrients<br>that your body needs. It is recommended that you try different foods and cooking methods to keep your diet varied.<br><br>We hope the above suggestions are helpful to you. If you have any questions or need further assistance, please feel free to contact us.	Rater1: Feedback given is too lengthy and more like a bot-generated response.<br><br>Rater2: The feedback given is too lengthy.<br><br>Rater3: None<br><br>Rater4: None
LLMDF	Personal Health Information<br><br>Gender: Female<br><br>Age: 33<br><br>Height: 167cm<br><br>Weight: 87.0kg	Lunch pairings are still fine, but adding a vegetable is recommended.	Rater1: Too simple and not tailored.<br><br>Rater2: Too simple and misguided. **(Knowledge error)**<br><br>Rater3: None<br><br>Rater4: None

	Labor intensity: Medium labor intensity<br><br>Dietary Information<br>Meal type: Lunch<br>Food intake: Curry pork chop rice, coffee		
LLMDF-EXP	Personal Health Information<br>Gender: Female<br>Age: 33<br>Height: 167cm<br>Weight: 87.0kg<br>Labor intensity: Medium labor intensity<br><br>Dietary Information<br>Meal type: Lunch<br>Food intake: Curry pork chop rice, coffee	Curry Pork Chop Rice is a high-calorie and high-fat food, which is not quite suitable for your health condition. It is recommended that you choose low-fat, high-fiber food, such as grilled chicken breast with vegetable salad or grilled fish with vegetables, which can provide more nutrition while controlling calorie intake. In addition, although coffee can be refreshing, excessive consumption can stimulate the nervous system and increase heart rate and blood pressure, so it is recommended to drink it in moderation.	Rater1: None<br>Rater2: Stiff expression.<br>Rater3: None<br>Rater4: None
Experts	Personal Health Information<br>Gender: Female<br>Age: 33<br>Height: 167cm<br>Weight: 87.0kg<br>Labor intensity: Medium labor intensity<br><br>Dietary Information<br>Meal type: Lunch<br>Food intake: Curry pork chop rice, coffee	Lunch is higher in energy and the meal is more homogenous. The amount of easy vegetables in the Curry Pork Chop Rice was on the low side. It is recommended to add some additional vegetables, especially increasing the proportion of dark vegetables (dark green, fuchsia, orange-yellow). Currently weight is in obesity, pork is high in energy, especially pork chops after frying higher energy, it is recommended to reduce consumption. Sources of high-quality protein are recommended poultry, aquatic products and soy products.	Rater1: The feedback is professional.<br>Rater2: None<br>Rater3: None<br>Rater4: None

GPT-4	Personal Health Information<br><br>Gender: Male<br><br>Age: 33<br><br>Height: 187cm<br><br>Weight: 90.0kg<br><br>Labor intensity: Light labor intensity<br><br><br>Dietary Information<br><br>Meal type: Lunch<br><br>Food intake: 16 leek and egg dumplings	Dear Client:<br><br>Based on the dietary information you have provided, I have made some preliminary assessments. First, from a recent lunch, your primary food intake was 16 leek and egg dumplings. This meal is relatively balanced in terms of nutrient distribution. Among other things, the eggs provide high-quality protein and essential fatty acids, which are good for the body to maintain basic metabolism and muscle growth, while the leeks provide abundant fiber and micronutrients, which are good for intestinal health and help provide essential vitamins.<br><br>However, flour products such as dumpling skins tend to overconsume carbohydrates and may increase your weight. As a 33-year-old, 187cm tall, 90.0kg light laborer, you should focus more on protein and fat intake, as well as reducing excessive carbohydrate intake.<br><br>The fact is that healthy eating habits are developed over time, and the possibility of bad ideas before has made it even more necessary for you to endure the challenges of trivial possibilities when switching to a better way of eating. In terms of tweaking your eating habits, I would suggest that you avoid overeating at breakfast by choosing more whole grains in your breakfast diet to increase your intake of fiber and complex carbohydrates. For lunch and dinner, you can moderately reduce the intake of cereals and increase the intake of vegetables and meat, so as to achieve the goal of balanced nutrition and dietary calorie control.<br><br>Eating seven to eight minutes of each meal is enough to avoid the appetite triggered by eating<br>too much at one time because you are too hungry, and that may lead to weight gain. A little change could be the start of a healthy life, and I believe you can do it. If there are any more detailed questions please feel free to ask me and I will be happy to help answer them. I hope you can maintain a healthy lifestyle and let's get into good eating habits together.<br><br>Good luck,<br>your dietitian.	Rater1: The feedback given is too lengthy.<br><br>Rater2: Feedback given is too lengthy with no practical advice.<br><br>Rater3: The feedback given is too lengthy.<br><br>Rater4: None
GPT-3.5	Personal Health Information<br><br>Gender: Male<br><br>Age: 33	Based on the dietary information you provided, I note that you consumed 16 leek and egg dumplings in your lunch. This food is high in starch and fat, and while they provide some energy, excessive intake may lead to weight gain and potential health problems. As a dietitian, I recommend that you include more	Rater1: The dietary feedback is not friendly to understand.

	Height: 187cm<br><br>Weight: 90.0kg<br><br>Labor intensity: Light labor intensity<br><br><br>Dietary Information<br><br>Meal type: Lunch<br><br>Food intake: 16 leek and egg dumplings	vegetables, fruits and protein sources in your diet to maintain a balanced nutrient intake for your body.<br><br>In response to your personal health information, you are a male, 33 years old, 187 cm in height, 90 kg in weight, and you work in a light labor-intensive job. In order to maintain a healthy lifestyle, I recommend that you develop a specific diet plan: increase your intake of vegetables and fruits, choose foods rich in protein and fiber, and moderate your intake of carbohydrates and fats. In addition, eating regularly, maintaining a varied diet and exercising moderately are also important factors in maintaining good health.<br><br>Finally, I encourage you to adopt healthy eating habits such as drinking water regularly, avoiding excessive salt, sugar and saturated fats, and eating regular meals. If you have any questions about your diet or need more help, please feel free to ask me. I hope you can achieve a healthier body and a healthier lifestyle through a sensible eating plan and lifestyle changes!	Rater2: Suggestions could be streamlined and recommendations made more specific.<br><br>Rater3: It's not practical.<br><br>Rater4: None
ChatGLM3-6B	Personal Health Information<br><br>Gender: Male<br><br>Age: 33<br><br>Height: 187cm<br><br>Weight: 90.0kg<br><br>Labor intensity: Light labor intensity<br><br><br>Dietary Information<br><br>Meal type: Lunch<br><br>Food intake: 16 leek and egg dumplings	Dear Client, according to the dietary information you provided, you consume mainly leek and egg dumplings, which is a nutrient-rich but also relatively high-calorie food. For your light labor intensity, you are advised to control your total calorie intake when consuming this type of food to avoid excessive intake leading to weight gain.<br><br>In terms of food choices, it is recommended that you increase your intake of vegetables and fruits to get more fiber and vitamins. For example, you can pair some green leafy vegetables such as spinach and lettuce with some fruits such as apples and bananas. This will ensure that you have a more balanced meal.<br><br>In addition, in order to maintain a healthy diet, it is recommended that you follow the principle of "three lows and one high", i.e. low salt, low sugar, low fat and high fiber. In your diet today, chive and egg dumplings are high in fat and salt, so it is recommended that you consume them in moderation and minimize the intake of other high-fat and high-salt foods.<br><br>Finally, it is recommended that you develop a sensible diet plan to determine your daily calorie intake and ensure that you are consuming adequate nutrients based on your labor intensity and age. If you have any questions, please feel free to ask me, I will be happy to provide you with professional nutritional guidance.	Rater1: Feedback given is too lengthy and more like a bot-generated response<br><br>Rater2: Like a bot-generated response.<br><br>Rater3: None<br><br>Rater4: None

		I wish you a healthy diet and a happy life!	
LLMDF	Personal Health Information<br><br>Gender: Male<br><br>Age: 33<br><br>Height: 187cm<br><br>Weight: 90.0kg<br><br>Labor intensity: Light labor intensity<br><br>Dietary Information<br><br>Meal type: Lunch<br><br>Food intake: 16 leek and egg dumplings	For lunch dumplings, the amount of vegetables is not enough, so it is recommended to increase the amount of vegetables, and the amount of vegetables is recommended to be 250 grams or more.	Rater1: The feedback is professional.<br><br>Rater2: None<br><br>Rater3: None<br><br>Rater4: None
LLMDF-EXP	Personal Health Information<br><br>Gender: Male<br><br>Age: 33<br><br>Height: 187cm<br><br>Weight: 90.0kg<br><br>Labor intensity: Light labor intensity<br><br>Dietary Information<br><br>Meal type: Lunch<br><br>Food intake: 16 leek and egg dumplings	The lunch only consisted of leek and egg dumplings, which was not nutritionally balanced. It is recommended to increase the intake of vegetables and proteins, such as with a vegetable salad and a serving of lean meat.	Rater1: Too simple and could be more comprehensive.<br><br>Rater2: Stiff expression.<br><br>Rater3: None<br><br>Rater4: None
Experts	Personal Health Information<br><br>Gender: Male<br><br>Age: 33	The variety of food for lunch is more limited. Vegetables in the range of 300-500 grams are recommended for the general population, and more vegetables are recommended for overweight people. The amount of vegetables in the dumplings is limited, so it is recommended to have another serving of vegetables with it later.	Rater1: None<br><br>Rater2: Stiff expression.<br><br>Rater3: None

	Height: 187cm<br><br>Weight: 90.0kg<br><br>Labor intensity: Light labor intensity<br><br><br>Dietary Information<br><br>Meal type: Lunch<br><br>Food intake: 16 leek and egg dumplings		Rater4: None
GPT-4	Personal Health Information<br><br>Gender: Male<br><br>Age: 43<br><br>Height: 163cm<br><br>Weight: 74.5kg<br><br>Labor intensity: Light labor intensity<br><br><br>Dietary Information<br><br>Meal type: Lunch<br><br>Food intake: Half a pancake + 1 corn + 3 small potatoes + chicken breast + sauteed lettuce + baby bok choy	Based on your lunch information, I see that you are making reasonable carbohydrate and protein choices, but note that some important dietary elements may have been omitted, including healthy fats and adequate fiber intake.<br><br>First, let's look at protein intake. You chose chicken breast for your lunch, which is a high-protein, low-fat source of food that has a positive effect on the body's ability to function properly. In addition, you chose pancakes, which provide a certain amount of carbohydrates that can be used as a source of energy.<br><br>However, the small potatoes and corn you consumed in your meal, while rich in carbohydrates, do not contain enough fiber to prevent you from feeling hungry or having blood sugar fluctuations. I would suggest that you try replacing or incorporating some whole grains, such as brown rice or oatmeal, to ensure a varied and nutritionally balanced diet.<br><br>Regarding vegetable choices, romaine lettuce and baby bok choy are both high in fiber and low in calories, and are good sources of increased satiety and vitamins and minerals. However, make sure that you allow vegetables to take up more than half of your meal plate at every meal, as this will help you meet your body's needs for a variety of nutrients.<br><br>Overall, your lunch food choices are relatively healthy, but there are small changes we can make to improve them further. For example, using healthy fats such as olive oil or walnut oil in your cooking, and eating as many colorful vegetables as possible to take in more nutrients. Keep your intake of these fats under strict control to avoid overdoing it.<br><br>Always remember that the goal of a diet is to fulfill the body's	Rater1: The feedback given is too lengthy.<br><br>Rater2: The feedback given is too lengthy and some of the content is wrong. **(Knowledge error)**<br><br>Rater3: None<br><br>Rater4: None

		needs, not just taste. However, it should also be an enjoyable experience, so remember to enjoy your food and just make more healthful decisions on choices and portion sizes.	
GPT-3.5	Personal Health Information<br><br>Gender: Male<br><br>Age: 43<br><br>Height: 163cm<br><br>Weight: 74.5kg<br><br>Labor intensity: Light labor intensity<br><br><br>Dietary Information<br><br>Meal type: Lunch<br><br>Food intake: Half a pancake + 1 corn + 3 small potatoes + chicken breast + sauteed lettuce + baby bok choy	Based on the information you provided about your lunch intake and your personal health information, I would like to give you some suggestions.<br><br>Firstly, your lunch meal contains some starchy foods such as pancakes and corn. It is recommended that you moderate your intake of these foods because too much starchy food tends to make your blood glucose fluctuate too much.<br><br>Secondly, your lunch intake has a single source of protein. It is recommended that you include other sources of protein in addition to chicken breast, such as tofu, fish or beans, to ensure that you get a balanced protein intake.<br><br>In addition, you should increase the variety and amount of vegetable foods in your meals, such as sautéed romaine lettuce and baby bok choy are great choices, but consider adding other colorful vegetables to get a wider variety of vitamins and minerals.<br><br>In addition, based on your personal health information, light labor-intensive work requires reasonable control of energy intake, plus moderate exercise to maintain a healthy weight and physical condition.<br><br>Finally, it is recommended that you minimize sugary and high-fat snacks and beverages, and drink plenty of water, tea, or fresh juices as beverages to maintain balance and variety in your diet in order to make it healthier. We hope you will take these tips and stick to your healthy eating habits to better protect and maintain your health. Please feel free to contact me if you have any questions or need further advice. I wish you good health!	Rater1: Feedback given is too lengthy and more like a bot-generated response.<br><br>Rater2: Responses could be more concise.<br><br>Rater3: The feedback given is too lengthy.<br><br>Rater4: None
ChatGLM3-6B	Personal Health Information<br><br>Gender: Male<br><br>Age: 43<br><br>Height: 163cm<br><br>Weight: 74.5kg	Hello, based on the dietary information you provided, your intake of half pancakes, corn, small potatoes, chicken breast, sautéed romaine lettuce, and baby bok choy, your diet contains some fats, carbohydrates, and proteins but lacks adequate dietary fiber and vitamins. I recommend that you increase your intake of vegetables and fruits in your next diet to help you achieve a balanced nutritional intake.<br><br>For your personal health information, you are a male, 43 years	Rater1: The feedback is not friendly to understand.<br><br>Rater2: Feedback given is too lengthy with no practical advice.<br><br>Rater3: None<br><br>Rater4: None

	Labor intensity: Light labor intensity<br><br>Dietary Information<br><br>Meal type: Lunch<br><br>Food intake: Half a pancake + 1 corn + 3 small potatoes + chicken breast + sauteed lettuce + baby bok choy	old, 163cm tall, 74.5kg, light labor. Based on this information, I recommend that you maintain a moderate diet and avoid excessive energy intake to maintain your healthy weight.<br><br>In developing your dietary pattern, it is recommended that you follow the dietary structure of "grains and cereals, vegetables and fruits, meat, eggs and milk, and soy and dairy products" to ensure that you are consuming all types of nutrients. In addition, it is recommended that you avoid excessive frying, stir-frying and other high-fat cooking methods, and choose low-fat cooking methods such as steaming, boiling and stewing.<br><br>In order to maintain healthy eating habits, I recommend you to set regular eating hours, avoid over-starvation or over-fullness, and maintain a regular meal schedule.<br><br>Lastly, I suggest you pay attention to dietary variety and avoid eating the same food all the time to ensure your body gets a variety of nutrients it needs. I hope my advice has been helpful and if you have any other questions, please feel free to ask me.	
LLMDF	Personal Health Information<br><br>Gender: Male<br><br>Age: 43<br><br>Height: 163cm<br><br>Weight: 74.5kg<br><br>Labor intensity: Light labor intensity<br><br>Dietary Information<br><br>Meal type: Lunch<br><br>Food intake: Half a pancake + 1 corn + 3 small potatoes + chicken breast + sauteed lettuce + baby bok choy	This is a great meal where you have a hearty meal with enough vegetables! Potatoes are a staple, and when you eat them, you can cut back on the staples and increase the veggies.	Rater1: None<br><br>Rater2: None<br><br>Rater3: None<br><br>Rater4: None
LLMDF-EXP	Personal Health Information<br><br>Gender: Male<br><br>Age: 43	The lunch was a good mix of vegetables and meat. Pancakes are high in calories and fat, so it is recommended to reduce intake or choose other low-calorie staples. Small potatoes are starchy foods and are recommended to be controlled in moderation. Chicken breasts are low-fat, high-protein meats that are great for your	Rater1: None<br><br>Rater2: None<br><br>Rater3: None

	Height: 163cm<br><br>Weight: 74.5kg<br><br>Labor intensity: Light labor intensity<br><br><br>Dietary Information<br><br>Meal type: Lunch<br><br>Food intake: Half a pancake + 1 corn + 3 small potatoes + chicken breast + sauteed lettuce + baby bok choy	light labor intensity. Sautéed romaine lettuce and baby bok choy are vegetables that are rich in fiber and vitamins and are good for your health. It is recommended that you maintain a balanced diet, increase the intake of vegetables and fruits, and control the intake of oil and starchy foods in moderation.	Rater4: None
Experts	Personal Health Information<br><br>Gender: Male<br><br>Age: 43<br><br>Height: 163cm<br><br>Weight: 74.5kg<br><br>Labor intensity: Light labor intensity<br><br><br>Dietary Information<br><br>Meal type: Lunch<br><br>Food intake: Half a pancake + 1 corn + 3 small potatoes + chicken breast + sauteed lettuce + baby bok choy	Lunch can be a good mix of food types, chicken breast is a good source of high-quality protein; vegetables are root vegetables and leafy vegetables, which are also very good; staple foods are coarse and fine, and the variety is relatively rich in terms of the angle, but the energy may be easy to overload, and it is necessary to pay attention to the amount of staple food.	Rater1: The feedback is professional.<br><br>Rater2: None<br><br>Rater3: None<br><br>Rater4: None
GPT-4	Personal Health Information<br><br>Gender: Female<br><br>Age: 54<br><br>Height: 156cm<br><br>Weight: 59.0kg	From the information you've provided, I can see that your concerns about your diet are centered on vegetable and quality protein intake, which is a very good place to start. Your choices, including a variety of vegetables, chicken muscle, and black rice are rich in a variety of micronutrients, vitamins, and proteins that are extremely beneficial to your health.<br><br>However, passion fruit soup can be sweet and it is recommended that you keep your sweet tooth in check to prevent blood sugar fluctuations and maintain good cardiovascular health. You can try	Rater1: The feedback is not friendly to understand.<br><br>Rater2: Recommendations are too lengthy and could be streamlined.<br><br>Rater3: The feedback given is too lengthy.<br><br>Rater4: None

	Labor intensity: Light labor intensity<br><br>Dietary Information<br><br>Meal type: Lunch<br><br>Food intake: Various vegetables, 1 serving of chicken breast, black rice, 1 bowl of passion fruit soup (tastes sweet)	using unsweetened yogurt or some lightly flavored fruits instead.<br><br>In addition, knowing that your activity intensity is light labor, depending on your age, gender and weight, I recommend that your daily calorie intake should be between 1,600 and 1,800 calories to maintain good health. In terms of dietary structure, you can focus on consuming enough vegetables and fruits, as well as appropriate intake of high-quality proteins such as fish, meat, eggs, milk and its products, coarse grains and nuts. At the same time, reduce the intake of oily and sugary foods.<br><br>Just as you choose chicken breast with a variety of vegetables, maintaining food variety is an important part of improving the quality of your diet. Each food has its own unique nutrient profile, and by choosing different types of foods, you can get a full and balanced range of nutrients.<br><br>In summary, your diet is already quite healthy, but there is still room for some minor adjustments. I encourage you to continue with this healthy diet and make minor improvements in areas such as sweets intake, dietary variety, and total calorie intake.	
GPT-3.5	Personal Health Information<br><br>Gender: Female<br><br>Age: 54<br><br>Height: 156cm<br><br>Weight: 59.0kg<br><br>Labor intensity: Light labor intensity<br><br>Dietary Information<br><br>Meal type: Lunch<br><br>Food intake: Various vegetables, 1 serving of chicken breast, black rice, 1 bowl of passion fruit soup (tastes sweet)	Based on the information you provided about your lunch, I can see that you are still eating a healthy mix of food. You consume a variety of vegetables, which is great because vegetables are rich in a variety of vitamins and fiber that help keep your body healthy. It is also good to choose chicken breast as a source of protein as it contains less fat than other meats. Black rice is a healthy grain that is rich in antioxidants and fiber, which helps to increase satiety. As for passion fruit soup, if you have a sweet tooth, you can consume it in moderation, but be careful to control your sugar intake.<br><br>Based on your personal health information, as a 54-year-old woman, your meals should contain enough protein and fruits and vegetables to fulfill your body's nutritional needs. Considering your light labor intensity, it is recommended that you moderate your carbohydrate intake and choose more proteins and fruits and vegetables that can help control your weight and maintain your health.<br><br>To maintain a healthy diet, it is recommended that you include protein, vegetables and grains in every meal and moderate your intake of oil, salt and sugar. It is also recommended that you drink plenty of water to keep your body well hydrated and promote metabolism and detoxification. Lastly, it is important to	Rater1: The feedback is too lengthy.<br><br>Rater2: The feedback could be streamlined<br><br>Rater3: The feedback given is too lengthy.<br><br>Rater4: None

		develop the habit of having regular meals, chewing slowly and not overeating, which will help control the amount of food you eat and maintain a healthy weight.<br><br>I hope the above suggestions are helpful to you, if you have any questions or need further guidance, please feel free to contact me. I wish you good health, a balanced diet and a happy life!	
ChatGLM3-6B	Personal Health Information<br><br>Gender: Female<br><br>Age: 54<br><br>Height: 156cm<br><br>Weight: 59.0kg<br><br>Labor intensity: Light labor intensity<br><br>Dietary Information<br><br>Meal type: Lunch<br><br>Food intake: Various vegetables, 1 serving of chicken breast, black rice, 1 bowl of passion fruit soup (tastes sweet)	Hello, based on the information you provided about your diet, I would like to make the following analysis and recommendations for you:<br><br>Your diet has a good intake of vegetables, which is a good start. However, your calorie intake comes mainly from chicken breast and passion fruit soup, which may lead to unbalanced nutrition. In order to stay healthy, it is recommended that you increase the proportion of vegetables, which can be multi-colored, to get more vitamins and minerals.<br><br>For meat, you consume one serving of chicken breast, which is a good source of protein. However, due to your high total calorie intake, it is recommended that you opt for leaner cuts of meat rather than too much fat.<br><br>In terms of staple food, black rice is a good choice as it is rich in fiber and helps in keeping you full. However, passion fruit soup is relatively high in calories and you are advised to consume it in moderation or opt for low-calorie soups such as tomato soup.<br><br>Your personal health information shows that you are in the normal range for your age and weight, but with low labor intensity. Therefore, in order to maintain good health, I recommend that you increase your daily activity level, such as walking or doing yoga, while maintaining a balanced and varied diet.<br><br>Overall, your diet is already good, but to maintain your health in a more holistic way, I would recommend increasing the proportion of vegetables in your diet, choosing leaner cuts of meat, moderating your intake of high-calorie soups, and increasing your daily activity level. I hope these suggestions are helpful to you.	Rater1: Feedback given is too lengthy and more like a bot-generated response.<br><br>Rater2: Like a bot-generated response and some of the content is wrong. **(Knowledge error)**<br><br>Rater3: There were professional errors. **(Knowledge error)**<br><br>Rater4: None
LLMDF	Personal Health Information<br><br>Gender: Female	Lunch was great, but would recommend switching to unsweetened soup would be better~	Rater1: Too simple and could be more comprehensive.

	Age: 54<br><br>Height: 156cm<br><br>Weight: 59.0kg<br><br>Labor intensity: Light labor intensity<br><br>Dietary Information<br><br>Meal type: Lunch<br><br>Food intake: Various vegetables, 1 serving of chicken breast, black rice, 1 bowl of passion fruit soup (tastes sweet)		Rater2: Too simple.<br><br>Rater3: Simple, clear, and very hands-on.<br><br>Rater4: None
LLMDF-EXP	Personal Health Information<br><br>Gender: Female<br><br>Age: 54<br><br>Height: 156cm<br><br>Weight: 59.0kg<br><br>Labor intensity: Light labor intensity<br><br>Dietary Information<br><br>Meal type: Lunch<br><br>Food intake: Various vegetables, 1 serving of chicken breast, black rice, 1 bowl of passion fruit soup (tastes sweet)	Vegetables and chicken breasts are good choices for lunch food intake, and black rice is also healthier. However, passion fruit soup is on the sweet side, so it is recommended to reduce the intake of sweets and consider choosing other low-sugar fruits as an alternative to the soup. In addition, you can add some high quality protein sources such as fish, beans or nuts to ensure a balanced diet and nutrient intake.	Rater1: None<br><br>Rater2: The advice given is relatively practical and professional, and the recommendations could be more comprehensive.<br><br>Rater3: None<br><br>Rater4: None
Experts	Personal Health Information<br><br>Gender: Female<br><br>Age: 54<br><br>Height: 156cm	The main combination of lunch is fine, with a choice of mixed grain rice for the main course and chicken breasts which are white meat, low in fat and a good source of high quality protein. Vegetables are currently not visible in terms of type and portion size, and it is recommended to choose more dark-colored vegetables. It is also recommended to reduce the consumption of sweet soups as the weight is currently high.	Rater1: None<br><br>Rater2: None<br><br>Rater3: None<br><br>Rater4: None

	Weight: 59.0kg<br><br>Labor intensity: Light labor intensity<br><br><br>Dietary Information<br><br>Meal type: Lunch<br><br>Food intake: Various vegetables, 1 serving of chicken breast, black rice, 1 bowl of passion fruit soup (tastes sweet)		
GPT-4	Personal Health Information<br><br>Gender: Male<br><br>Age: 20<br><br>Height: 181cm<br><br>Weight: 115.0kg<br><br>Waistline: 110.0cm<br><br>Labor intensity: Light labor intensity<br><br><br>Dietary Information<br><br>Meal type: Dinner<br><br>Food intake: Bok choy 70g; Rice 200g; Bok choy egg custard 60g; Chicken thigh 70g.	Based on the information you provided about your dinner diet and your personal health information, here's some of my professional feedback.<br><br>First of all, you did a great job of including vegetables, rice, eggs and chicken in your dinner, which is a well-balanced diet containing carbohydrates, vitamins, minerals and protein. The baby bok choy and bok choy egg custard provided you with plenty of vitamins and minerals, while the rice and chicken thighs gave you energy and protein.<br><br>However, given your weight and waistline, I would recommend that you keep an eye on your calorie intake and make sure that you don't take in more calories than you burn. Your workload is light and you don't need a lot of calories to burn. Rice intake may be slightly higher, with 200 grams of rice containing about 400 calories. Try reducing the portion size of rice to minimize your carbohydrate intake, and also try replacing it with lower sugar options such as brown rice or mixed grains.<br><br>Meanwhile, for protein intake, chicken thighs are rich in protein but also contain more fat, so it is recommended that you choose skinless or lean chicken as your protein source. Also, protein intake should be combined with workouts that practice muscle strength to help build and maintain muscle tissue.<br><br>In conclusion, although your diet contains a variety of essential nutrients, you need to focus on your carbohydrate and fat intake and adjust your diet to suit your health needs and lifestyle. Increasing your exercise and lowering your dietary intake of high-fat, high-carbohydrate foods will help control your weight and reduce your risk of developing chronic diseases.	Rater1: The feedback is too lengthy.<br><br>Rater2: The feedback is too lengthy.<br><br>Rater3: None<br><br>Rater4: None

GPT-3.5	Personal Health Information<br><br>Gender: Male<br><br>Age: 20<br><br>Height: 181cm<br><br>Weight: 115.0kg<br><br>Waistline: 110.0cm<br><br>Labor intensity: Light labor intensity<br><br><br>Dietary Information<br><br>Meal type: Dinner<br><br>Food intake: Bok choy 70g; Rice 200g; Bok choy egg custard 60g; Chicken thigh 70g.	Based on the information you provided about your dinner diet and your personal health information, I will give you some suggestions and guidance.<br><br>First of all, you are not eating a lot of protein at dinner, so I would suggest you to add some other sources of protein, such as beans, nuts, or fish, to make sure that you have a variety of proteins. In addition, your intake of fruits and vegetables is low. You may want to increase the amount of vegetables you eat, such as increasing the amount of greens or adding other types of vegetables to get more vitamins and minerals.<br><br>Considering your heavier body weight, it is recommended to control your intake of staple foods by reducing the portion of rice or choosing whole grains instead of white rice to lower your calorie intake. In addition, it is recommended that you maintain regular and moderate exercise to help reduce weight and increase your body's metabolic rate.<br><br>Lastly, depending on your labor intensity, you should be careful not to overdo it with your dinner intake. Moderation is enough to avoid energy accumulation and body burden. I hope the above suggestions are helpful to you, and I wish you good health and a happy life! If you need more help or have other questions, feel free to contact me.	Rater1: Feedback given is too lengthy and more like a bot-generated response.<br><br>Rater2: The advice given is relatively practical and professional, and it is suggested that it could be streamlined a bit.<br><br>Rater3: None<br><br>Rater4: None
ChatGLM3-6B	Personal Health Information<br><br>Gender: Male<br><br>Age: 20<br><br>Height: 181cm<br><br>Weight: 115.0kg<br><br>Waistline: 110.0cm<br><br>Labor intensity: Light labor intensity<br><br><br>Dietary Information<br><br>Meal type: Dinner<br><br>Food intake: Bok choy 70g; Rice 200g;	Dear Client, Based on the dietary information you have provided, it is a very good sign that your dinner intake is mainly based on vegetables and grains. Vegetables such as chard and bok choy are rich in vitamins, minerals and dietary fiber, which can help you maintain a healthy digestive and immune system. Rice, on the other hand, as a staple food, provides you with enough energy to maintain good stamina.<br><br>However, your protein intake is relatively low. It is recommended that you increase your intake of some high-quality proteins, such as lean meat, fish, beans, etc.,<br>in your next dinner. In addition, eggs in egg custard are also a good source of protein.<br><br>Considering your age, height and weight, I suggest you keep your calorie intake between 1500-1800 kcal per meal to maintain a healthy weight. For specific food choices, you can choose low-calorie, high-nutrient foods such as lean meat, fish, tofu and vegetables.	Rater1: The feedback is not friendly to understand.<br><br>Rater2: The feedback is too lengthy.<br><br>Rater3: None<br><br>Rater4: None

	Bok choy egg custard 60g; Chicken thigh 70g.	In addition, in order to maintain a healthy diet, it is recommended that you avoid too much greasy food and high-sugar food, as well as drinking too much alcohol. It is also very important to drink enough water every day.<br><br>I hope these suggestions are helpful, and if you have any questions or need further guidance, please feel free to ask me. I wish you good health!	
LLMDF	Personal Health Information<br><br>Gender: Male<br><br>Age: 20<br><br>Height: 181cm<br><br>Weight: 115.0kg<br><br>Waistline: 110.0cm<br><br>Labor intensity: Light labor intensity<br><br>Dietary Information<br><br>Meal type: Dinner<br><br>Food intake: Bok choy 70g; Rice 200g; Bok choy egg custard 60g; Chicken thigh 70g.	The overall dinner is fine, but be careful not to consume too much staple food (rice, noodles, etc.) at a later stage, as too much staple food can easily lead to nutritional imbalance and weight gain.	Rater1: Not tailored.<br><br>Rater2: Too simple and should be more comprehensive.<br><br>Rater3: None<br><br>Rater4: None
LLMDF-EXP	Personal Health Information<br><br>Gender: Male<br><br>Age: 20<br><br>Height: 181cm<br><br>Weight: 115.0kg<br><br>Waistline: 110.0cm<br><br>Labor intensity: Light labor intensity	The combination of dishes for dinner is okay, but it is recommended to reduce the intake of rice and increase the intake of vegetables. In addition, chicken thighs are high in fat, so it is recommended to reduce the intake or choose skinless chicken thigh meat. In addition, in addition to pay attention to control the portion of rice, you can try to choose brown rice to increase satiety and dietary fiber intake.	Rater1: The feedback is professional.<br><br>Rater2: None<br><br>Rater3: None<br><br>Rater4: None

	Dietary Information<br><br>Meal type: Dinner<br><br>Food intake: Bok choy 70g; Rice 200g; Bok choy egg custard 60g; Chicken thigh 70g.		
Experts	Personal Health Information<br><br>Gender: Male<br><br>Age: 20<br><br>Height: 181cm<br><br>Weight: 115.0kg<br><br>Waistline: 110.0cm<br><br>Labor intensity: Light labor intensity<br><br>Dietary Information<br><br>Meal type: Dinner<br><br>Food intake: Bok choy 70g; Rice 200g; Bok choy egg custard 60g; Chicken thigh 70g.	Dinner with follow the formula of carbohydrate + protein + vegetables, dishes are also relatively light, the variety of ingredients can be more rich, dinner energy should be lower, you can replace the chicken legs with lower energy and fat fish and shrimp.	Rater1: None<br><br>Rater2: Suggestions could be more comprehensive.<br><br>Rater3: None<br><br>Rater4: None
GPT-4	Personal Health Information<br><br>Gender: Female<br><br>Age: 19<br><br>Height: 167cm<br><br>Weight: 75.0kg<br><br>Labor intensity: Light labor intensity<br><br>Dietary Information	Dear Customer.<br><br>Thank you for sharing your dietary information and personal health information with us. First, I will provide some feedback and suggestions for your food intake information.<br><br>Judging from your dinner, your diet is relatively balanced. Papaya is a nutritious fruit with high levels of vitamin C and antioxidants, which are very beneficial to your health. However, sausages are usually high in fat and sodium, and although the serving size is only 25 grams, long-term consumption may still affect your health. Wheat flour is a great source of carbohydrates and a major supplier of our daily energy.<br><br>In terms of nutritional intake, your meals need to have more	Rater1: The feedback is too lengthy.<br><br>Rater2: The feedback is lengthy, like bot-generated response.<br><br>Rater3: The diet of the case was clearly very unhealthy, but this feedback says: a balanced diet. **(Knowledge error)**<br><br>Rater4: None

	Meal type: Dinner<br><br>Food intake: Papaya (Senna) 150g; Sausage 25g; Wheat flour 200g.	protein and fiber. Including foods such as tofu, low-fat dairy products, lean meats and fish in your diet will provide high-quality protein, while increasing your intake of whole grains, vegetables and fruits will effectively supply fiber. These are all important components of a healthy diet.<br><br>For your dietary pattern, I would recommend that you continue to maintain your current healthy eating habits while appropriately reducing your intake of processed foods. For your height and weight, you are on the high side, which may increase the risk of developing cardiovascular diseases. Therefore, I recommend you to control your calorie intake and increase your physical activity as appropriate to promote healthy weight management.<br><br>Overall, a healthy diet should focus on balanced nutrition and eating in moderation, not only the type of food but also the amount of food. Your diet improvement and management is a long-term process, and I look forward to us working together on this process. You are always welcome to ask questions or seek further advice. We are always here to support your health.<br><br>Wishing you good health.<br><br>Your Dietitian	
GPT-3.5	Personal Health Information<br><br>Gender: Female<br><br>Age: 19<br><br>Height: 167cm<br><br>Weight: 75.0kg<br><br>Labor intensity: Light labor intensity<br><br><br>Dietary Information<br><br>Meal type: Dinner<br><br>Food intake: Papaya (Senna) 150g; Sausage 25g; Wheat flour 200g.	Based on the information you provided about your dinner intake and your personal health information, I'd like to give you some suggestions. First of all, papaya is a fruit rich in vitamin C and fiber, which can help boost immunity and promote digestive health. Sausage is tasty but high in saturated fat and salt, so it is recommended to reduce it in moderation or choose a healthy protein source instead. Wheat flour is a staple food, but you need to be careful about the amount you consume, and it is recommended to keep it in moderation with a balanced mix of vegetables, protein foods and other nutrients.<br><br>Based on your gender, age, height, weight and labor intensity, I recommend that you take a balanced intake of vegetables, fruits, whole grains, and protein foods at each meal, control the intake of fats and sugars, ensure that the total calories per day are controlled within the appropriate range, and pay attention to maintaining an appropriate amount of water intake and physical activity.<br><br>It is recommended that you develop a healthy dietary pattern, have three meals a day at regular intervals, diversified and	Rater1: None<br><br>Rater2: Stiff expression.<br><br>Rater3: None<br><br>Rater4: None

		colorful, avoid overeating and high-sugar and high-fat eating habits, maintain a moderate amount of exercise, develop a good lifestyle, gradually adjust the dietary structure, and adhere to it, you can see the changes in your body and the enhancement of your health. I hope you can continue to maintain healthy eating habits, not only is it good for your health, but it will also strengthen your resistance and improve your quality of life. I wish you good health and a happy life!	
ChatGLM3-6B	Personal Health Information<br><br>Gender: Female<br><br>Age: 19<br><br>Height: 167cm<br><br>Weight: 75.0kg<br><br>Labor intensity: Light labor intensity<br><br><br>Dietary Information<br><br>Meal type: Dinner<br><br>Food intake: Papaya (Senna) 150g; Sausage 25g; Wheat flour 200g.	Dear Client, According to the dietary information you have provided, you have consumed some papaya, sausage and wheat flour for your dinner. From these foods, papaya is rich in vitamin C and fiber and can be a good source in your diet. However, your dinner intake is high in sausage and wheat flour, which may provide too many calories and fat.<br><br>In order to maintain a healthy diet, I suggest you pay attention to the following points in your upcoming dinners:<br><br>1. Increase your vegetable intake: Vegetables are a great source of fiber and vitamins. You can try to increase your intake of vegetables such as green leafy vegetables, carrots, cucumbers, etc.<br><br>2. Reduce meat intake: Meat foods such as sausages are high in fat and cholesterol, which may affect your health. It is recommended to reduce the intake of meat, especially meat with high saturated fat, such as beef and pork.<br><br>3. Increase your intake of whole grains: Whole grains such as wheat flour can provide a longer-lasting feeling of satiety, which can help control your weight. You can increase your intake of whole grains in your future diet.<br><br>4. Maintain a varied diet: A varied diet helps you to consume a variety of nutrients. We recommend that you try to eat a variety of foods, such as fruits, nuts, beans, etc., at your next dinner.<br><br>We hope these suggestions will help you with your diet. Please feel free to let me know if you have any questions or need further guidance. Best wishes for your health!	Rater1: Feedback given is too lengthy and more like a bot-generated response.<br><br>Rater2: The feedback could be more concise.<br><br>Rater3: Incorrect advice on meat. **(Knowledge error and misunderstanding)**<br><br>Rater4: None
LLMDF	Personal Health Information<br><br>Gender: Female	The amount of vegetables at dinner is low, and sausages are processed foods, so it is recommended to eat them sparingly.	Rater1: Feedback could be more comprehensive.<br><br>Rater2: None

	Age: 19<br><br>Height: 167cm<br><br>Weight: 75.0kg<br><br>Labor intensity: Light labor intensity<br><br>Dietary Information<br><br>Meal type: Dinner<br><br>Food intake: Papaya (Senna) 150g; Sausage 25g; Wheat flour 200g.		Rater3: None<br><br>Rater4: None
LLMDF-EXP	Personal Health Information<br><br>Gender: Female<br><br>Age: 19<br><br>Height: 167cm<br><br>Weight: 75.0kg<br><br>Labor intensity: Light labor intensity<br><br>Dietary Information<br><br>Meal type: Dinner<br><br>Food intake: Papaya (Senna) 150g; Sausage 25g; Wheat flour 200g.	The dinner choices were a little less sensible. Sausage is a processed meat product that is high in fat and salt and is recommended to be eaten in moderation. Wheat flour is a starchy food, but excessive intake can lead to blood sugar fluctuation and it is recommended to control the intake. To better meet nutritional requirements, it is recommended to increase the intake of vegetables and proteins, such as adding a vegetable salad, fish or chicken.	Rater1: The dietary feedback is professional.<br><br>Rater2: None<br><br>Rater3: None<br><br>Rater4: None
Experts	Personal Health Information<br><br>Gender: Female<br><br>Age: 19<br><br>Height: 167cm<br><br>Weight: 75.0kg<br><br>Labor intensity: Light labor intensity	The variety of ingredients for dinner is low, and it is recommended to have at least 6 or more items in a meal to ensure a balanced intake of nutrients.	Rater1: The feedback could be more tailored.<br><br>Rater2: The feedback should be more comprehensive.<br><br>Rater3: None<br><br>Rater4: None

	Dietary Information<br><br>Meal type: Dinner<br><br>Food intake: Papaya (Senna) 150g; Sausage 25g; Wheat flour 200g.		
GPT-4	Personal Health Information<br><br>Gender: Male<br><br>Age: 34<br><br>Height: 174cm<br><br>Weight: 79.0kg<br><br>Labor intensity: Light labor intensity<br><br>Dietary Information<br><br>Meal type: Dinner<br><br>Food intake: A bowl of rice, fish head soup, beef, leafy greens, tomato scrambled eggs.	First of all, the information on your dinner intake shows that you have a relatively balanced diet. Rice provides the main carbohydrate energy source, beef and fish head provide high quality protein, and bok choy and tomato scrambled eggs provide essential vegetables and vitamins. However, one thing you need to be aware of is that beef is high in fat, so if your body is trying to lose weight or your cholesterol level is high, try to control your meat intake by opting for chicken breast or fish instead.<br><br>With this in mind, I would recommend that you add some fruits and vegetables, such as oranges, apples, lemons, etc., to boost your Vitamin C intake and boost your immune system. Meanwhile, in order to reduce fat intake, terrible replace beef with chicken breast or fish.<br><br>In terms of dietary pattern formulation, it is recommended that you maintain the habit of having sufficient breakfast, moderate lunch and less dinner, which is conducive to maintaining gastrointestinal health and also helps to lose weight. Moreover, your work intensity is relatively low, and you need to pay attention to moderate exercise after meals, such as walking or doing exercises, to help the digestion of food.<br><br>You are encouraged to maintain your current healthy eating habits, but please also take care to eliminate unhealthy foods, such as fried foods and sugary drinks, as much as possible. Based on your height and weight, you are on the heavier side, so reducing staple foods, increasing fruits and vegetables, and exercising in moderation can be very helpful to your health.	Rater1: The feedback is too lengthy.<br><br>Rater2: The feedback should be more concise.<br><br>Rater3: None<br><br>Rater4: None
GPT-3.5	Personal Health Information<br><br>Gender: Male<br><br>Age: 34<br><br>Height: 174cm	Based on the dinner information and personal health information you provided, I have analyzed your diet. You chose a bowl of rice, fish head soup, beef, bok choy, and scrambled eggs with tomatoes for dinner, which is a well-balanced mix of staples, proteins, and vegetables, but there is still some room for improvement.<br><br>First, you are consuming a moderate amount of staples for	Rater1: None<br><br>Rater2: None<br><br>Rater3: None<br><br>Rater4: None

	Weight: 79.0kg<br><br>Labor intensity: Light labor intensity<br><br><br>Dietary Information<br><br>Meal type: Dinner<br><br>Food intake: A bowl of rice, fish head soup, beef, leafy greens, tomato scrambled eggs.	dinner, but consider opting for whole grain rice to increase your fiber and vitamin intake. Additionally, the fish head soup and beef provide quality protein, but other protein sources such as seafood could be added for variety. Bok choy and scrambled eggs with tomatoes provide plenty of vitamins and fiber, but fruit intake can be increased to provide more vitamins and antioxidants.<br><br>Based on your personal health information, I recommend that you maintain this eating pattern, but need to be careful to control your fat and sugar intake and avoid excess processed foods and sweets. Also, depending on your light labor intensity, consider adding moderate exercise to strengthen your body and boost your metabolism.<br><br>Lastly, we hope you can maintain a healthy diet, choose more natural ingredients, have a balanced diet, make reasonable combinations, eat regularly and in moderation, avoid overeating, maintaining good eating habits is very important to your health. If you have any questions or need further guidance, please feel free to contact me. I wish you good health and a happy life!	
ChatGLM3-6B	Personal Health Information<br><br>Gender: Male<br><br>Age: 34<br><br>Height: 174cm<br><br>Weight: 79.0kg<br><br>Labor intensity: Light labor intensity<br><br><br>Dietary Information<br><br>Meal type: Dinner<br><br>Food intake: A bowl of rice, fish head soup, beef, leafy greens, tomato scrambled eggs.	Dear Client, according to the dietary information you provided, you consumed more carbohydrates and proteins at dinner and relatively less vegetables and fruits. This dietary structure may lead to unbalanced nutrition. You are advised to pay attention to the following points in your future diet:<br><br>1. Optimize food choices: While ensuring balanced nutrition, you can appropriately increase the intake of vegetables and fruits, such as diversifying the types of vegetables and choosing brightly colored vegetables to obtain more vitamins and minerals.<br><br>2. Control the intake of staple food: Rice is the main source of staple food, but excessive intake of rice will lead to fluctuation of blood sugar. It is recommended that you reduce your rice intake appropriately by pairing it with some coarse grains, such as red beans, mung beans, millet, etc., to provide a more stable blood glucose supply.<br><br>3. Increase protein intake: Foods such as fish head soup and beef are important sources of protein, but too much protein intake may lead to increased kidney burden. It is recommended that you control your protein intake, especially animal protein, and consider increasing the intake of plant-based proteins such as	Rater1: Feedback given is too lengthy and more like a bot-generated response.<br><br>Rater2: Some of the content is wrong. **(Knowledge error)**<br><br>Rater3: None<br><br>Rater4: None

		beans and eggs.<br><br>4. Maintain dietary diversity: It is recommended that you try to maintain a variety of foods in each meal to meet your body's needs for various nutrients. You can appropriately increase the intake of foods such as nuts and whole grains to provide more healthy fats and dietary fiber.<br><br>5. Pay attention to the dynamic balance of your diet: Do not neglect between-meal snacks and additional meals. Appropriate intake of healthy snacks, such as nuts and fruits, can help replenish energy and keep blood sugar stable.<br><br>I hope these suggestions will help you with your diet and wish you a healthy lifestyle!	
LLMDF	Personal Health Information<br><br>Gender: Male<br><br>Age: 34<br><br>Height: 174cm<br><br>Weight: 79.0kg<br><br>Labor intensity: Light labor intensity<br><br>Dietary Information<br><br>Meal type: Dinner<br><br>Food intake: A bowl of rice, fish head soup, beef, leafy greens, tomato scrambled eggs.	Meat and veggies for dinner was very good, keep it up!	Rater1: Too simple and not tailored.<br><br>Rater2: Too simple and not tailored.<br><br>Rater3: None<br><br>Rater4: None
LLMDF-EXP	Personal Health Information<br><br>Gender: Male<br><br>Age: 34<br><br>Height: 174cm<br><br>Weight: 79.0kg	The dinner was well balanced with a good mix of food, including a main course, meat, vegetables and soup. It is recommended to control the amount of rice and try to limit it to about half a bowl to control calorie intake. It is also recommended to increase the variety and quantity of vegetables. Try adding other green leafy vegetables such as spinach and lettuce to increase fiber and vitamin intake. It is also recommended to exercise moderately after dinner to help digestion and burn calories. Keep up the good eating habits, cheers!	Rater1: The feedback is not friendly to understand.<br><br>Rater2: None<br><br>Rater3: None<br><br>Rater4: None

	Labor intensity: Light labor intensity<br><br>Dietary Information<br><br>Meal type: Dinner<br><br>Food intake: A bowl of rice, fish head soup, beef, leafy greens, tomato scrambled eggs.		
Experts	Personal Health Information<br><br>Gender: Male<br><br>Age: 34<br><br>Height: 174cm<br><br>Weight: 79.0kg<br><br>Labor intensity: Light labor intensity<br><br>Dietary Information<br><br>Meal type: Dinner<br><br>Food intake: A bowl of rice, fish head soup, beef, leafy greens, tomato scrambled eggs.	Dinner has done a good job of combining meat and vegetables, so please keep it up! For the main course, it is recommended to add coarse grains for dietary fiber, which will increase satiety and help to reduce the amount of food. Scrambled eggs with tomatoes are cooked with a high level of oil, so it is recommended to replace them with a more low-oil dish. In order to control weight, the amount of vegetables to ensure that half of a meal, a meal to achieve 2-3 kinds of vegetables, not only conducive to energy control, but also can supplement dietary fiber and a variety of vitamins and minerals.	Rater1: The feedback is professional.<br><br>Rater2: None<br><br>Rater3: None<br><br>Rater4: None
GPT-4	Personal Health Information<br><br>Gender: Female<br><br>Age: 37<br><br>Height: 160cm<br><br>Weight: 60.9kg<br><br>Labor intensity: Light labor intensity<br><br>Dietary Information	Dear Client, With the information you have provided, I have analyzed your nutritional intake as follows:<br><br>In your dinner, I see that you are not only consuming enough carbohydrates (rice), but also a rich source of proteins (scrambled eggs with shrimp), as well as minerals and vitamins (bok choy and potatoes). Meanwhile, ribs and rib soup are a good source of quality minerals (such as calcium and phosphorus). This is a relatively healthy and balanced dietary choice.<br><br>However, you may need to keep an eye on your food intake. The amount of rice in your dinner is more than sufficient. If you are trying to control your weight and cope with a lighter labor-intensive lifestyle, you may want to consider reducing your carbohydrate intake slightly or adding some low-fat protein foods	Rater1: Feedback given is too lengthy and more like a bot-generated response.<br><br>Rater2: None<br><br>Rater3: None<br><br>Rater4: None

	Meal type: Dinner<br><br>Food intake: A bowl of rice, one bowl of green vegetables, half bowl of shrimp scrambled eggs, half bowl of potato ribs, 2 bowls of rib soup.	and high-fiber foods such as vegetables and fruits to enhance satiety and prevent over-starvation in the evening.<br><br>We recommend that you change your dinner to ½ bowl of rice, 1 bowl of greens, ½ bowl of shrimp scrambled eggs, ½ bowl of potato ribs and 1 bowl of rib soup. Also, try to make sure that at least 1-2 meals in a day are varied to ensure that your body is getting different types of nutrients. Consistently maintaining a healthy diet is what promotes good health.<br><br>Also, remember to maintain proper exercise on top of your diet to improve your metabolism and cardiorespiratory fitness. Your health depends not only on what you eat, but also on your lifestyle.	
GPT-3.5	Personal Health Information<br><br>Gender: Female<br><br>Age: 37<br><br>Height: 160cm<br><br>Weight: 60.9kg<br><br>Labor intensity: Light labor intensity<br><br>Dietary Information<br><br>Meal type: Dinner<br><br>Food intake: A bowl of rice, one bowl of green vegetables, half bowl of shrimp scrambled eggs, half bowl of potato ribs, 2 bowls of rib soup.	Based on the information you provided regarding your dinner intake, I note that your dinner was relatively well-balanced and included staples, protein, and vegetables, which is good to see. However, I note that the variety of foods in your dinner is slightly more homogenous and recommend that you add a wider variety of vegetables and protein sources to your daily diet to ensure that you are getting a more well-rounded nutritional profile.<br><br>In response to your personal health information, as a 37-year-old woman, you are well within the normal range for height and weight. With light labor intensity, your caloric needs will not be too high, but you still need to make sure that you are consuming enough protein, vegetables, and fruits to maintain your body's proper function and metabolism.<br><br>To help you develop a healthy eating pattern, I recommend that you diversify your food choices to include whole grains, protein foods (meats, beans, nuts), vegetables and fruits. Reasonable control of your intake of staple foods and meats, as well as an abundance of vegetables and fruits, will increase satiety and maintain a balanced nutrient intake for your body.<br><br>In addition, I encourage you to maintain healthy eating habits and recommend that you undergo regular dietary assessment and adjustment to rationalize your diet according to your physical condition and personal goals. It is also recommended that you maintain a moderate amount of exercise to boost your metabolism and improve your body's immunity, which will help keep you healthy.<br><br>Anyway, I hope my suggestions are helpful to you, and if you	Rater1: None<br><br>Rater2: None<br><br>Rater3: None<br><br>Rater4: None

		have any questions or need more advice, please feel free to contact me. I wish you a healthy and happy life!	
ChatGLM3-6B	Personal Health Information<br><br>Gender: Female<br><br>Age: 37<br><br>Height: 160cm<br><br>Weight: 60.9kg<br><br>Labor intensity: Light labor intensity<br><br><br>Dietary Information<br><br>Meal type: Dinner<br><br>Food intake: A bowl of rice, one bowl of green vegetables, half bowl of shrimp scrambled eggs, half bowl of potato ribs, 2 bowls of rib soup.	Dear Client, According to the dietary information you provided, your dinner intake is mainly composed of rice, green vegetables, shrimp scrambled eggs, potato ribs and rib soup. From the perspective of healthy eating, this dinner has some nutritional balance, but there are areas for improvement.<br><br>First of all, rice is a staple food and consuming too much of it will lead to excessive intake of carbohydrates and sugar, which is not good for your health. It is recommended that you reduce your rice intake appropriately or choose healthier alternatives such as brown rice or whole wheat bread.<br><br>Secondly, bok choy and potato ribs are a good source of vegetables and protein, respectively, with a higher intake. However, scrambled eggs with shrimp are high in fat, so it is recommended that you reduce your intake of such foods in moderation to avoid consuming too much fat.<br><br>In addition, rib soup is a good soup choice to provide your body with the water and nutrients it needs. However, it should be noted that the soup may be high in fats and sodium, so it is recommended that you reduce your consumption to avoid excessive fats and sodium.<br><br>Finally, your personal health information shows that you are female, 37 years old, 160cm tall, 60.9kg in weight, and your labor intensity is light. Based on this information, it is recommended that you pay attention to the following points in your diet:<br><br>1. maintain a varied diet with more vegetables, fruits, whole grains and proteins, and less oily, high-sugar and high-salt foods.<br>2. maintain an appropriate amount of food and drink, and avoid over-starvation or over-fullness.<br>3. maintain a good daily routine, avoid staying up late and get enough sleep.<br>4. maintain a proper amount of activity, according to your physical condition and needs, moderate exercise, in order to maintain good health.<br><br>I hope the above suggestions are helpful to you, if you have	Rater1: The feedback is too lengthy.<br><br>Rater2: Lengthy replies and some of the content is wrong. **(Knowledge error)**<br><br>Rater3: None<br><br>Rater4: None

		other questions or need further guidance, please feel free to consult me. I wish you good health and happiness!	
LLMDF	Personal Health Information<br><br>Gender: Female<br><br>Age: 37<br><br>Height: 160cm<br><br>Weight: 60.9kg<br><br>Labor intensity: Light labor intensity<br><br>Dietary Information<br><br>Meal type: Dinner<br><br>Food intake: A bowl of rice, one bowl of green vegetables, half bowl of shrimp scrambled eggs, half bowl of potato ribs, 2 bowls of rib soup.	You have eaten a healthy meal, so please keep it up! However, potatoes are a staple and it is recommended that you occasionally replace them with other vegetables such as carrots, cucumbers and tomatoes.	Rater1: Too simple and the feedback should be more comprehensive.<br><br>Rater2: The feedback could be more comprehensive.<br><br>Rater3: None<br><br>Rater4: None
LLMDF-EXP	Personal Health Information<br><br>Gender: Female<br><br>Age: 37<br><br>Height: 160cm<br><br>Weight: 60.9kg<br><br>Labor intensity: Light labor intensity<br><br>Dietary Information<br><br>Meal type: Dinner<br><br>Food intake: A bowl of rice, one bowl of green vegetables, half bowl of shrimp scrambled eggs, half bowl of potato ribs, 2 bowls of rib soup.	The dinner is a good mix of meat and vegetables, but the portion of rice is slightly too much, so it is recommended to reduce it to half a bowl. Also, the pork chop soup is high in fat, so you can reduce it moderately or choose clear soup instead. Continuing to maintain a meat-vegetable combination and being mindful of meal portion control will help maintain a healthy weight.	Rater1: The feedback is not friendly to understand.<br><br>Rater2: None<br><br>Rater3: None<br><br>Rater4: None

Experts	Personal Health Information<br><br>Gender: Female<br><br>Age: 37<br><br>Height: 160cm<br><br>Weight: 60.9kg<br><br>Labor intensity: Light labor intensity<br><br><br>Dietary Information<br><br>Meal type: Dinner<br><br>Food intake: A bowl of rice, one bowl of green vegetables, half bowl of shrimp scrambled eggs, half bowl of potato ribs, 2 bowls of rib soup.	Dinner has both meat and vegetables, but the amount of animal food is high and the amount of vegetables is low, so it is recommended to increase the amount of vegetables and reduce the amount of meat. Vegetables should account for half of a meal, at least 2-3 kinds of dark green, red-orange, purple and other dark-colored vegetables. Meat to ensure that the amount of the palm of your hand can be, at the same time, meat should be less to choose ribs, ribs higher fat content, it is recommended that more low-fat high-protein fish, shrimp, shellfish and other aquatic products. Eggs less choose scrambled eggs, high oil content, can choose to steam eggs.	Rater1: The feedback is professional.<br><br>Rater2: None<br><br>Rater3: None<br><br>Rater4: None
GPT-4	Personal Health Information<br><br>Gender: Male<br><br>Age: 34<br><br>Height: 172cm<br><br>Weight: 86.2kg<br><br>Labor intensity: Medium labor intensity<br><br><br>Dietary Information<br><br>Meal type: Dinner<br><br>Food intake: A bowl of rice, braised pork with preserved vegetables, seaweed and egg drop soup.	Thank you for the dietary information. After analyzing the information, I found out that your dinner consists of rice, buckwheat with preserved vegetables and seaweed and egg soup. Dunked Pork with Preserved Vegetables is a nutritious dish that contains a high amount of protein and calories, making it suitable for a moderately labor-intensive job. However, it should be noted that the fat content in the Duckling Pork with Preserved Vegetables is also relatively high, which may lead to excessive calorie intake. In addition, Nori Egg Drop Soup is a nutritionally balanced meal that has both protein and minerals.<br><br>Healthy dietary choices can include more vegetables and legumes. For example, you can try pairing it with some leafy greens and tofu. Such a meal is not only nutritionally balanced but also provides enough satiety.<br><br>For the rice portion, if you need to manage your weight, you may consider reducing the portion of rice in moderation or replacing it with whole grains such as brown rice or oats.<br><br>With reference to your age, height and weight, I would suggest you to engage in regular weight management so that you can avoid excessive weight gain. At the same time, try to do as much exercise as possible to increase your body's metabolism and help	Rater1: Feedback given is too lengthy.<br><br>Rater2: Feedback given is too lengthy and more like a bot-generated response.<br><br>Rater3: None<br><br>Rater4: None

		burn off excess energy.<br><br>Set a clear dietary pattern, such as consuming three meals a day at regular intervals and try to get enough vegetables daily. Adhering to and adjusting your eating habits over time is excellent for both weight management and overall health. All changes take time and there is no need to rush them, take your time and you will get there.	
GPT-3.5	Personal Health Information<br><br>Gender: Male<br><br>Age: 34<br><br>Height: 172cm<br><br>Weight: 86.2kg<br><br>Labor intensity: Medium labor intensity<br><br>Dietary Information<br><br>Meal type: Dinner<br><br>Food intake: A bowl of rice, braised pork with preserved vegetables, seaweed and egg drop soup.	Based on the information you provided about your dinner, I can see that your diet lacks vegetable and fruit intake. Though tasty, the pork with preserved vegetables is high in fat and calories, so I suggest you control your consumption in moderation.<br><br>In order to maintain a healthy lifestyle, I recommend you to increase your intake of vegetables and fruits by choosing brightly colored vegetables and fruits such as red peppers, broccoli and strawberries, which can provide more vitamins and fiber. It is also recommended that you reduce your intake of fatty foods by choosing low-fat cooking methods such as baking, steaming and stewing to reduce unnecessary calorie intake.<br><br>For your gender, age, weight and labor intensity, I recommend you to maintain a moderate dietary calorie intake, control the portion of staple foods and meat, increase the proportion of vegetables and fruits and whole grains, and balance the mix of protein sources, such as fish and beans. At the same time, rationalize your dietary structure and time, make sure your meals are regular and stay away from high-sugar, high-salt and high-oil foods.<br><br>We hope that the above suggestions can help you adjust your dietary structure and develop good eating habits, which will in turn promote your health and enhance your quality of life. If you have any questions or need more guidance, please feel free to contact me. I wish you good health and happy eating!	Rater1: None<br><br>Rater2: None<br><br>Rater3: None<br><br>Rater4: None
ChatGLM3-6B	Personal Health Information<br><br>Gender: Male<br><br>Age: 34<br><br>Height: 172cm<br><br>Weight: 86.2kg	Dear Client, according to the dietary information you provided, your dinner intake is mainly based on rice, meat and soup, and this dietary pattern lacks intake of vegetables and fruits, which may lead to unbalanced nutrition. You are advised to increase your intake of vegetables and fruits in your future dinners to improve your dietary fiber intake, which is good for digestion and maintaining good health.<br><br>In addition, you mentioned that your labor intensity is moderate,	Rater1: Feedback given is too lengthy and more like a bot-generated response.<br><br>Rater2: Stiff expression.<br><br>Rater3: None<br><br>Rater4: None

	Labor intensity: Medium labor intensity<br><br>Dietary Information<br><br>Meal type: Dinner<br><br>Food intake: A bowl of rice, braised pork with preserved vegetables, seaweed and egg drop soup.	so it is recommended that you increase your protein intake in your diet, such as fish, chicken, and tofu, in order to meet your body's amino acid and protein needs and maintain muscle health.<br><br>In addition, according to your personal health information, you seem to have a slight obesity problem. It is recommended that you reduce your intake of high-calorie foods, such as desserts and high-fat meats, as well as increase aerobic exercise, such as running and swimming, to help reduce your weight and maintain a healthy body shape.<br><br>Lastly, please pay attention to maintaining a good diet, avoid over-eating and maintain a moderate food intake, as well as maintaining a good work schedule and getting enough sleep, which is very important for good health. I hope the above suggestions are helpful to you, if you have any other questions or need further assistance, please feel free to contact me.	
LLMDF	Personal Health Information<br><br>Gender: Male<br><br>Age: 34<br><br>Height: 172cm<br><br>Weight: 86.2kg<br><br>Labor intensity: Medium labor intensity<br><br>Dietary Information<br><br>Meal type: Dinner<br><br>Food intake: A bowl of rice, braised pork with preserved vegetables, seaweed and egg drop soup.	Today's meal was generally good, it's the amount of vegetables that is still on the low side, note for later in the day, vegetables still need to be secured.	Rater1: None<br><br>Rater2: Feedback could be more comprehensive.<br><br>Rater3: None<br><br>Rater4: None
LLMDF-EXP	Personal Health Information<br><br>Gender: Male<br><br>Age: 34<br><br>Height: 172cm	The buckwheat with preserved vegetables in dinner is a high-calorie and high-fat food, so it is recommended to reduce the intake or choose other low-fat meat food instead. Seaweed and eggdrop soup is a good choice, but it is recommended to increase the intake of vegetables, you can choose to add more bok choy or other green leafy vegetables to enrich the nutrition of the meal. In addition, pay attention to the portion of rice and try to keep it within one bowl to avoid excessive intake of carbohydrates.	Rater1: None<br><br>Rater2: The tone is a little stiff.<br><br>Rater3: None<br><br>Rate4: None

	Weight: 86.2kg<br><br>Labor intensity: Medium labor intensity<br><br><br>Dietary Information<br><br>Meal type: Dinner<br><br>Food intake: A bowl of rice, braised pork with preserved vegetables, seaweed and egg drop soup.		
Experts	Personal Health Information<br><br>Gender: Male<br><br>Age: 34<br><br>Height: 172cm<br><br>Weight: 86.2kg<br><br>Labor intensity: Medium labor intensity<br><br><br>Dietary Information<br><br>Meal type: Dinner<br><br>Food intake: A bowl of rice, braised pork with preserved vegetables, seaweed and egg drop soup.	Dinner is relatively simple with few ingredients. It is recommended to enrich the variety of ingredients, with at least 6 or more types in a meal. The main food should be a combination of coarse and fine, and vegetables should be half of the meal, with at least 2-3 kinds. Dumplings with preserved vegetables are high in fat, so it is not recommended to choose them. For meat, choose low-fat, high-protein fish, shrimp and aquatic products, as well as lean beef, pork, chicken, and so on.	Rater1: The feedback is professional.<br><br>Rater2: None<br><br>Rater3: None<br><br>Rater4: None
GPT-4	Personal Health Information<br><br>Gender: Female<br><br>Age: 42<br><br>Height: 160cm<br><br>Weight: 61.0kg<br><br>Labor intensity: Light labor intensity	Dear Client.<br><br>First of all, I want to thank you for the dinner meal and personal health information. Let me provide you with some professional feedback on your diet.<br><br>Your dinner choices indicate that you are already trying to maintain a balanced diet. Beef is an excellent source of protein, which is essential for maintaining good muscle and immune function. Broccoli, on the other hand, is rich in fiber, which aids in digestion, and also provides plenty of vitamins, including vitamins C and K, as well as minerals.	Rater1: Feedback given is too lengthy and more like a bot-generated response.<br><br>Rater2: The feedback is too lengthy.<br><br>Rater3: The feedback is too lengthy.<br><br>Rater4: None

	Dietary Information<br><br>Meal type: Dinner<br><br>Food intake: Beef, broccoli	Referring to your personal health information, you are a 42-year-old woman with a height of 160cm and a weight of 61.0kg. Based on your information, it is recommended that your daily caloric intake energy is at approximately 1,800-2,000 kcal to meet the energy requirements for light labor intensity.<br><br>However, as there is only beef and broccoli for dinner, it may be slightly monotonous from a nutritional point of view. To be more nutritionally complete, it is recommended that you add some whole grains and legumes, which provide the complex carbohydrates your body needs. Additionally, a moderate increase in food sources of unsaturated fats such as seafood, nuts and seeds will also be beneficial.<br><br>In conclusion, I encourage you to maintain this attitude of realizing the importance of a healthy diet and suggest that along with maintaining such a diet, you also engage in regular moderate exercise for better health.<br><br>Remember, it is important to find a balanced eating pattern that you can maintain, rather than a strict diet for a short period of time. I look forward to chatting with you more in depth about your eating habits and health goals in future chats.<br><br>With best wishes for good health!<br><br>Your Nutritionist.	
GPT-3.5	Personal Health Information<br><br>Gender: Female<br><br>Age: 42<br><br>Height: 160cm<br><br>Weight: 61.0kg<br><br>Labor intensity: Light labor intensity<br><br>Dietary Information<br><br>Meal type: Dinner<br><br>Food intake: Beef, broccoli	Based on the information you provided about your dinner, I can see that your dietary choices are relatively simple, but both beef and broccoli are very healthy ingredients. Beef is rich in protein and iron, which help boost immunity and promote muscle growth, while broccoli is rich in vitamin C and folate, which help maintain healthy skin and prevent anemia.<br><br>As a dietitian, I recommend that you include more vegetables and grains in your dinner to ensure a balanced nutritional intake. You may consider adding colorful fruits and vegetables such as carrots, tomatoes, and strawberries to increase your intake of dietary fiber and antioxidants. Also, choosing whole grains, such as brown rice and whole wheat bread, will help provide long-lasting energy and help control blood sugar levels.<br><br>In addition, based on your personal health information, I recommend that you control the amount of fats and sugar intake in your diet and avoid excessive salt and processed foods. Try to	Rater1: The feedback is too lengthy.<br><br>Rater2: The tone is a little stiff.<br><br>Rater3: The feedback is too lengthy.<br><br>Rater4: None

		choose foods with simple and nutritious cooking methods, such as steaming, boiling and baking, to minimize unnecessary calorie intake.<br><br>Lastly, I encourage you to maintain a healthy diet, drink plenty of water, exercise moderately, and keep a good routine to achieve a healthy lifestyle. Feel free to contact me if you need more dietary advice or guidance. I wish you good health!	
ChatGLM3-6B	Personal Health Information<br><br>Gender: Female<br><br>Age: 42<br><br>Height: 160cm<br><br>Weight: 61.0kg<br><br>Labor intensity: Light labor intensity<br><br>Dietary Information<br><br>Meal type: Dinner<br><br>Food intake: Beef, broccoli	Client's dietary feedback:<br><br>Analyzing the attributes of the client's food intake: Your dinner intake of protein and vegetables is a good start. However, care needs to be taken to ensure that the source of your protein intake is not only meat, but also other protein sources such as legumes and fish. Vegetable intake is also important as they provide vitamins, minerals and fiber.<br><br>Provide healthy food choices: for meat, you might consider choosing leaner cuts of meat such as chicken, turkey or fish over beef. For vegetables, you may want to try more greens such as spinach, lettuce and beets.<br><br>Help your client develop a dietary pattern: It is recommended that you adopt a balanced dietary pattern that includes grains and cereals, protein sources, vegetables and fruits. In addition, you need to be careful to control food intake to maintain a healthy weight.<br><br>Encourage your clients to maintain healthy eating habits: In order to stay healthy, you need to maintain a consistent diet and avoid over-consumption of foods that are high in calories, fat and sugar. You should also exercise regularly to help burn off excess calories.<br><br>Advising clients to modify their diet: You may want to consider increasing your intake of fruit and fiber to help improve gut health and keep blood sugar levels stable. In addition, you may need to reduce your salt and sugar intake to help prevent conditions such as high blood pressure and diabetes.<br><br>To summarize: You are consuming some healthy foods for dinner, but need to improve further. You are advised to adopt a balanced dietary pattern and increase your intake of fruits and	Rater1: The feedback is too lengthy.<br><br>Rater2: None<br><br>Rater3: None<br><br>Rater4: None

		vegetables. Please also take care to control your food intake and maintain a stable diet.	
LLMDF	Personal Health Information<br><br>Gender: Female<br><br>Age: 42<br><br>Height: 160cm<br><br>Weight: 61.0kg<br><br>Labor intensity: Light labor intensity<br><br>Dietary Information<br><br>Meal type: Dinner<br><br>Food intake: Beef, broccoli	The dinner pairing is good, but there is not enough staple food, not enough rice. It is recommended to have about 100g of rice for the main meal.	Rater1: The feedback could be more comprehensive.<br><br>Rater2: None<br><br>Rater3: None<br><br>Rater4: None
LLMDF-EXP	Personal Health Information<br><br>Gender: Female<br><br>Age: 42<br><br>Height: 160cm<br><br>Weight: 61.0kg<br><br>Labor intensity: Light labor intensity<br><br>Dietary Information<br><br>Meal type: Dinner<br><br>Food intake: Beef, broccoli	It is good to choose beef and broccoli for dinner. Beef is rich in protein and iron, and broccoli is rich in vitamin C and fiber, which are very important for maintaining good health. It is recommended to add some staple food to your dinner, such as rice or noodles, to provide enough energy. Also, consider adding some other vegetables such as carrots or tomatoes to increase your vitamin and mineral intake. Maintaining this healthy diet, along with a moderate amount of exercise, can help you maintain a healthy lifestyle.	Rater1: None<br><br>Rater2: None<br><br>Rater3: None<br><br>Rater4: None
Experts	Personal Health Information<br><br>Gender: Female<br><br>Age: 42	Dinner is relatively simple, with only 2 kinds of ingredients, only beef and broccoli, and lack of staple food. It is recommended to enrich the variety of ingredients, at least 6 kinds or more in a meal. Following the formula of carbohydrates + protein + vegetables, the staple food should be a combination of coarse and fine, vegetables should be half of the meal, and at least 2-3 kinds	Rater1: The feedback is professional.<br><br>Rater2: None<br><br>Rater3: None

	Height: 160cm<br><br>Weight: 61.0kg<br><br>Labor intensity: Light labor intensity<br><br><br>Dietary Information<br><br>Meal type: Dinner<br><br>Food intake: Beef, broccoli	of meat should be chosen, including low-fat and high-protein fish, shrimp and aquatic products, as well as lean beef, pork and chicken.	Rater4: None

Supplementary Material Table S7

Distribution of the three types of hallucination across models in the human evaluation experiment

	ChatGLM3-6B	GPT-3.5	GPT-4	LLMDF	LLMDF-EXP
**Knowledge error:** The feedback has nutrition factual mistakes.	6	1	2	4	0
**Inconsistency**: LLMs produce contradictory information when handling complex dietary situations.	1	1	1	0	0
**Misunderstanding**: LLMs misinterpret scenario details provided in prompts.	5	0	0	0	0

Note: No comments revealed any hallucination issues in the feedback from LLMDF-EXP and human experts.

Supplementary Material Table S8

Examples of the three types of hallucination, knowledge error part in red color, inconsistency part in purple and misunderstanding part in yellow color

Hallucination Type	Model	Scene	Feedback
Knowledge error	ChatGLM3-6B	Personal Health Information<br><br>Gender: Female<br><br>Age: 19<br><br>Height: 166cm<br><br>Weight: 71.5kg<br><br>Waistline: 87.0cm<br><br>Labor intensity: Medium labor intensity<br><br>Dietary Information<br><br>Meal type: Breakfast<br><br>Food intake: Millet pumpkin porridge, fired leek dumplings, egg	Dear Client, Based on the dietary information you have provided, I have noticed that you are consuming more high-calorie foods such as millet pumpkin porridge and leek dumplings for breakfast, as well as fewer protein sources such as eggs. This dietary pattern may not be meeting the nutritional requirements you need, especially protein and fiber.<br><br>To ensure your health, I recommend that you increase your protein and fiber intake at future breakfasts. You can choose whole wheat bread or whole wheat toast instead of leek dumplings, and fruit or nuts instead of millet pumpkin porridge. In addition, eggs are a good source of protein, but consider increasing your intake of other protein foods such as lean meat, fish and tofu.<br><br>In addition, I have noticed that your diet lacks sufficient vegetables and fruits, which may result in inadequate vitamin and mineral intake. Therefore, I recommend you to increase the intake of vegetables and fruits in your meals, such as you can include fresh fruits or vegetable juices in your breakfast, or consume more vegetable salads in your lunch and dinner.<br><br>Finally, I recommend that you maintain a well-balanced diet, avoid over-consumption of high-calorie foods, and maintain a moderate level of physical activity to help maintain your healthy weight. If you have any questions or need further dietary advice, please feel free to contact me.
Knowledge error	LLMDF	Personal Health Information<br><br>Gender: Female<br><br>Age: 38<br><br>Height: 165cm<br><br>Weight: 76.8kg<br><br>Labor intensity: Medium labor intensity	Scrambled eggs are a good choice, but it is recommended to add a serving of vegetables, such as cucumbers and carrots.

		Dietary Information<br><br>Meal type: Breakfast<br><br>Food intake: One plate of egg fried rice	
Inconsistency	GPT-4	Personal Health Information<br><br>Gender: Male<br><br>Age: 29<br><br>Height: 174cm<br><br>Weight: 74.6kg<br><br>Labor intensity: Light labor intensity<br><br>Dietary Information<br><br>Meal type: Breakfast<br><br>Food intake: One egg, one vegetable bun, one soy milk (less sugar)	Based on the information you provided about your breakfast, your breakfast consisted of eggs, vegetable packets, and soymilk, constituting a more balanced intake of carbohydrates, proteins, and vitamins. Eggs are rich in high-quality protein, vegetable buns provide essential carbohydrates, and soy milk gives you plant-based protein and micronutrients.<br><br>However, the fat content of this breakfast meal may be on the low side. Given your age, gender, and weight, your basal metabolic rate is quite high, and it may be helpful to increase your intake of healthy fats, such as by adding some milk or whole wheat bread, which can add calories as well as keep you full.<br><br>Your work intensity is classified as light labor, so your calorie consumption will not be high. As breakfast is the start of the day, you can make small adjustments, such as replacing a vegetable bun with whole wheat bread and adding a fruit to supplement your vitamin intake.<br><br>Remember, a well-balanced, nutrient-dense diet and regular exercise are your keys to maintaining a healthy weight and boosting your body's immunity. If necessary, consider increasing your intake of fresh vegetables and fruits at different times of the day as appropriate. Maintaining a varied diet will help you consume a more complete range of nutrients. I hope these suggestions are helpful and look forward to better management of your weight and health!
Misunderstanding	ChatGLM3-6B	Personal Health Information<br><br>Gender: Female<br><br>Age: 19<br><br>Height: 167cm<br><br>Weight: 75.0kg	Dear Client, According to the dietary information you have provided, you have consumed some papaya, sausage and wheat flour for your dinner. From these foods, papaya is rich in vitamin C and fiber and can be a good source in your diet. However, your dinner intake is high in sausage and wheat flour, which may provide too many calories and fat.<br><br>In order to maintain a healthy diet, I suggest you pay attention to the following points in your upcoming dinners:

		Labor intensity: Light labor intensity<br><br>Dietary Information<br><br>Meal type: Dinner<br><br>Food intake: Papaya (Senna) 150g; Sausage 25g; Wheat flour 200g.	1. Increase your vegetable intake: Vegetables are a great source of fiber and vitamins. You can try to increase your intake of vegetables such as green leafy vegetables, carrots, cucumbers, etc.<br><br>2. Reduce meat intake: Meat foods such as sausages are high in fat and cholesterol, which may affect your health. It is recommended to reduce the intake of meat, especially meat with high saturated fat, such as beef and pork.<br><br>3. Increase your intake of whole grains: Whole grains such as wheat flour can provide a longer-lasting feeling of satiety, which can help control your weight. You can increase your intake of whole grains in your future diet.<br><br>4. Maintain a varied diet: A varied diet helps you to consume a variety of nutrients. We recommend that you try to eat a variety of foods, such as fruits, nuts, beans, etc., at your next dinner.<br><br>We hope these suggestions will help you with your diet. Please feel free to let me know if you have any questions or need further guidance. Best wishes for your health!
